# Improved Combustion Enthalpy Predictions Obtained by DFT-Based Methods through Empirical Correction: A Study of Cycloparaffins for Sustainable Aviation Fuel

**DOI:** 10.1021/acsomega.6c08844

**Published:** 2026-10-22

**Authors:** Thiago Soares Silva, Luiz Fernando de Araujo Ferrão, Douglas Henrique Pereira

**Affiliations:** † Postgraduate Program in Chemistry, Department of Chemistry, 74360 Federal University of Tocantins, Campus Gurupi-Badejós, P.O. Box 66, Gurupi, Tocantins 77 402-970, Brazil; ‡ Department of Chemistry, Aeronautics Institute of Technology, Praça Marechal Eduardo Gomes, 50, Vila das Acácias, São José dos Campos, São Paulo 12228-900, Brazil

## Abstract

The growing demand for aviation fuels and the progressive depletion of petroleum resources pose challenges related to transportation logistics, pollution, and technological development, making the advancement of sustainable aviation fuels (SAFs) imperative. In this context, computational research plays a critical role in optimizing production methodologies and predicting compounds with physicochemical properties comparable to those of conventional kerosenes. In this context, this study evaluated the performance of different DFT approximations (B3LYP, M06-2X, and M08-HX) and composite methodologies (G4 and G4-MP2) in predicting combustion enthalpies of organic molecules with high potential as SAF candidates. Initially, the experimental enthalpies of combustion were analyzed to assess the accuracy of each theoretical approach. The analysis was extended to cycloparaffins synthesized, which are promising SAF candidates due to their high energy content and suitable flash and freezing points. The results showed that the relative error tended to stabilize for molecules with higher combustion energies. The enthalpy of combustion calculated by the M08-HX method presented errors comparable to those of G4-MP2. The DFT methods demonstrated greater accuracy when employing the combustion energy correction proposed in this study, highlighting the viability of density functional theory (DFT) approximations. The analysis of complex structures at a significantly lower computational cost than composite methodological approaches indicates that the careful selection and empirical refinement of theoretical methods allow for a more accurate prediction of the combustion enthalpies.

## Graphical abstract

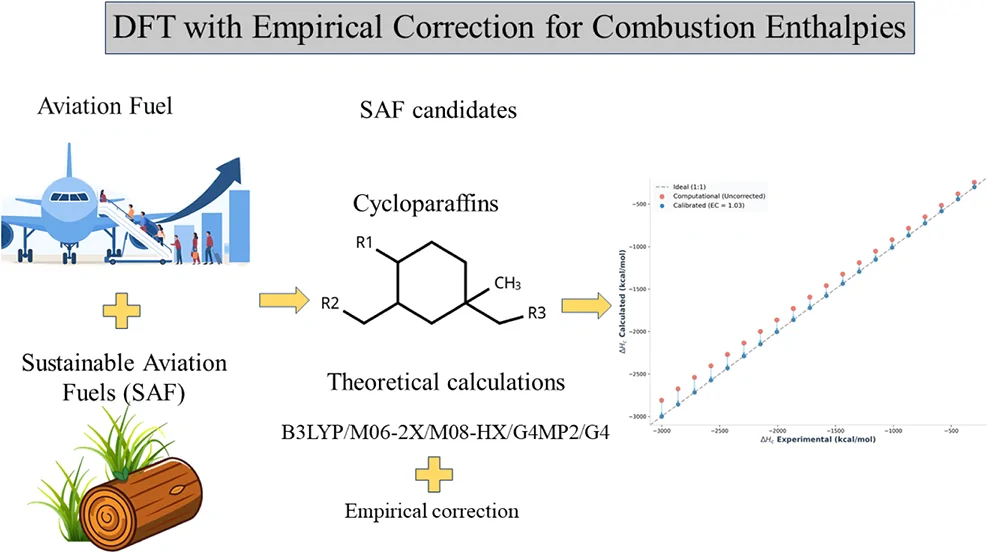

## 1 Introduction

Global demand for energy carriers increases annually, driven by industrial activity, freight and passenger transportation, and electricity generation.^1,2^ Currently, these compounds are predominantly derived from fossil resources (oil, natural gas, and coal), and their combustion emits various air pollutants, including carbon dioxide (CO_2_), carbon monoxide (CO), unburned hydrocarbons (HC), nitrogen oxides (NO_*x*_), sulfur dioxide (SO_2_), and particulate matter (PM).^2,3^

Air transportation has expanded steadily due to its logistical efficiency, increasing demand for aviation kerosene.^1,4^ These fuels are primarily produced from petroleum, and in response to the environmental impact of their emissions, the International Civil Aviation Organization (ICAO) has promoted multilateral agreements through national and international protocols aimed at progressively reducing greenhouse gas emissions from civil aviation.^5^

One strategy to mitigate emissions in the aviation sector is the adoption of sustainable aviation fuels (SAFs).^4,5^ SAFs are liquid aviation fuels produced from renewable or low-carbon sources and are intended to partially or fully replace fossil-derived aviation kerosene^6^ while maintaining compatibility with existing aircraft, engines, and infrastructure. Its main molecular components can be obtained through different SAF production routes involving physical processes (chipping, grinding, and milling), chemical processes (pyrolysis, catalytic cracking, hydrotreatment, and Fischer–Tropsch), and biological approaches (microorganisms, microalgae, and others).^4,7^

Aviation kerosenes such as JET-A and RP3 obtained in petroleum refineries are composed of molecules that may exhibit different degrees of branching in their molecular structures, including high quantities of paraffins, cycloparaffins, aromatics, and olefins.^8–10^ To optimize the physicochemical properties of SAF, blends containing different classes of synthetic molecules can be formulated, particularly cycloparaffins and aromatics, which play an important role in aviation fuel performance and compatibility requirements.^2^ These blends can be further improved with additives such as antifreezes, anticorrosion agents, and oxidative stabilizers, while preserving the safety and performance requirements of jet engines.^10,11^ Sacia et al.^12^ proposed a methodology to obtain cycloparaffin and aromatic structures (Figure 1) with different branchings to be used in SAFs.

**Figure 1:**
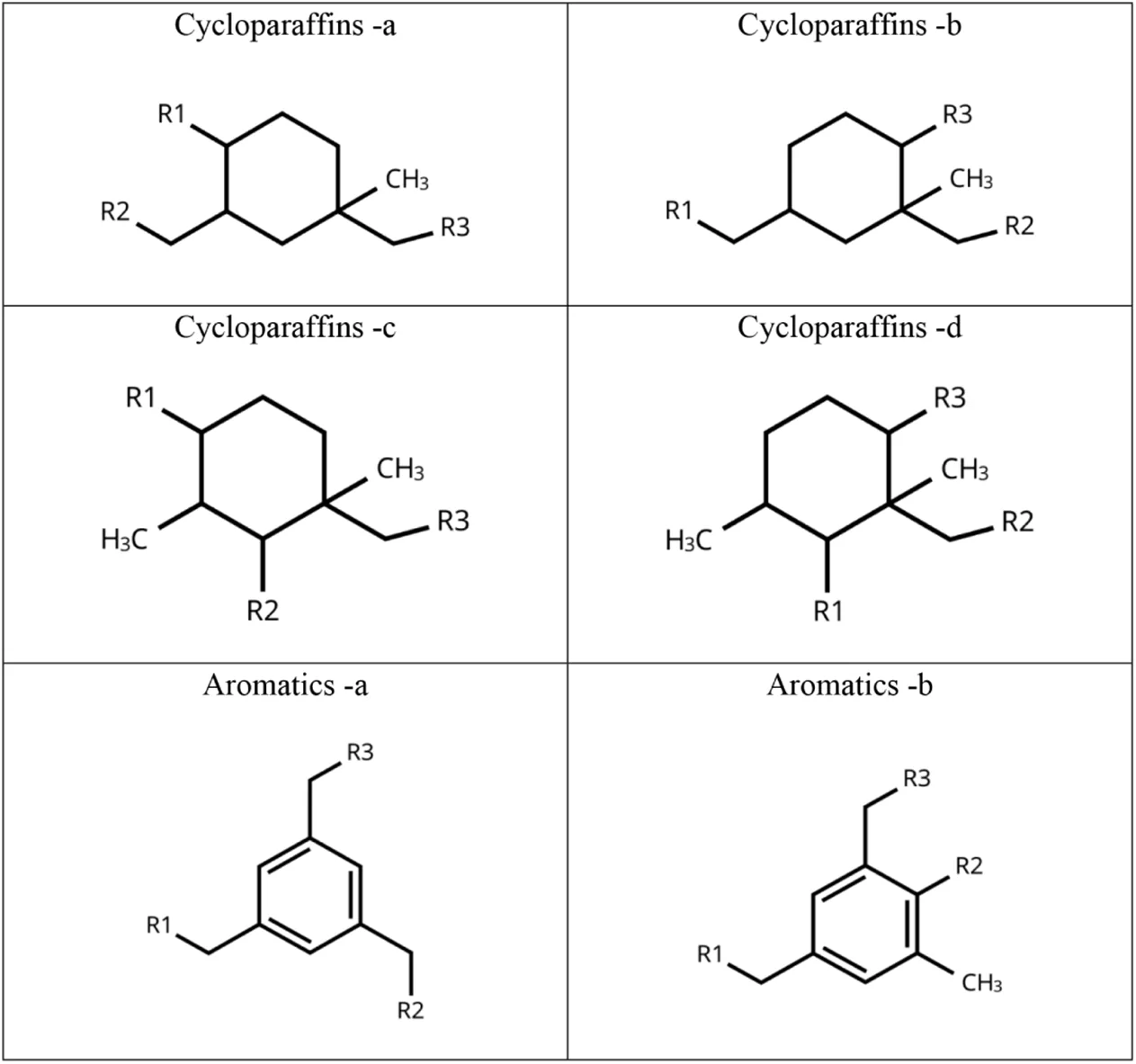
Different molecular structures of cycloparaffins and aromatics. Rn branches (R1, R2, R3) can be occupied by methyl (−CH_3_), ethyl (−C_1_H_5_), propyl (−C_3_H_7_), or butyl (−C_4_H_9_). Adapted from Sacia et al.^12^

Currently, numerous experimental studies on energetic molecules have been reported in the literature, motivated by the ongoing search for improved efficiency and performance in aviation fuels.^13^ Nonetheless, thermochemical analyses cover a wide range of structures, making them expensive compared to computational predictive analysis methods. Empirical approaches^14^ and composite methods (aLL5 protocol)^15–18^ were used to evaluate the liquid and gaseous enthalpy of formation of aliphatic cyclic compounds and their alkyl derivatives present in aviation fuels. The results from quantum methods indicate deviations of up to 1.6 kJ mol^–1^, which are values close to the uncertainty of the experimental data.^16^ Other studies are focused on obtaining sets of molecules for the development of databases containing theoretical calculations obtained by quantum methods.^13^ On the other hand, the methods based on DFT approximations^19–25^ have been widely applied due to their reliable accuracy in molecular geometry calculations,^13^ and with new functionals^24,25^ improving accuracy for enthalpies, reaction energies, kinetics, and noncovalent interactions. Thus, computational theoretical chemistry enables the prediction of physicochemical properties with accuracy approaching experimental values, providing valuable information for aviation fuel applications.^16,26^ In this context, the present study obtained the enthalpy of combustion through different hybrid methods based on DFT with empirical corrections, to be subsequently applied in the analysis of molecules intended for sustainable fuels.

## 2 Methodology

The experimental enthalpy of formation (Δ_f_H_gas,exp_) values for H_2_O, O_2_, CO_2_ and 89 organic molecules in the gaseous state were collected from the literature,^27^ including 20 linear *n*-alkanes, 32 cycloalkanes, 18 alcohols, 13 aldehydes, and 6 ketones. The stoichiometry of the combustion reaction for the studied species was obtained from eq 1, yielding the lower heating value (LHV), or the lower combustion enthalpy (Δ_c_H° = −LHV), where all products are gaseous, thereby not taking into account the latent heat of water vaporization:

1 $${C_{a}H_{b}O_{c}}_{\left(g\right)}+\left(a+\frac{b}{4}-\frac{c}{2}\right){O_{2}}_{\left(g\right)}\to aCO_{2\left(g\right)}+\frac{b}{2}H_{2}O_{\left(g\right)}$$

where “*a*”, “*b*”, and “*c*” represent the numbers of carbons (C), hydrogens (H), and oxygens (O) present in the molecule, and *n* is the number of molecules.

The eq 2 was used to obtain the experimental combustion enthalpies (Δ_c_H°_exp_ = −LHV):

2 $$\Delta_{c}{H^{{}^{\circ}}}_{\exp}=\left(X_{CO_{2}}\Delta_{f}{H^{{}^{\circ}}}_{{CO}_{2}(g)}\right)+\left(X_{H_{2}O}\Delta_{f}{H^{{}^{\circ}}}_{H_{2}O_{\left(g\right)}}\right)-\left(\Delta_{f}{H^{{}^{\circ}}}_{C_{a}H_{b}O_{c_{\left(g\right)}}}\right)-\left(X_{O_{2}}\Delta_{f}{H^{{}^{\circ}}}_{{O_{2}}_{\left(g\right)}}\right)$$

For theoretical calculations, all molecular geometries were constructed using the GaussView program.^28^ Electronic structure calculations, including energy minimization and vibrational frequency analysis, were performed using the Gaussian 16 software package.^29^ No negative frequencies were found, indicating that the optimized geometries are true minima on the potential-energy surface. Density functional theory (DFT) methods, including B3LYP, M06-2X, and M08-HX, were employed with a 6-311++G­(d,p) basis set. The UltraFine integration grid that is the default in Gaussian 16 was used in all calculations. No dispersion effects were used in the simulations.

The composite methods G4 and G4MP2 were also applied due to their high accuracy.^30^ The energy of the G4 method can be determined by eq 3.^31^

3 $$E_{G4}=E\left[\mathrm{MP4}/\mathrm{6‐31G}\left(d\right)\right]+{\Delta E}_{+}+{\Delta E}_{\mathrm{2df},p}+{\Delta E}_{\mathrm{CCSD}(T)}+{\Delta E}_{\mathrm{G3largeXP}}+{\Delta E}_{\mathrm{HF}}+E_{\mathrm{SO}}+E_{\mathrm{ZPE}}+E_{\mathrm{HLC}}$$

G4 energy features corrections for: diffuse functions (Δ*E*_+_), polarization functions (Δ*E*_2df,p_), an electron correlation correction superior to fourth order (Δ*E*_CCSD(T)_), a basis set correction not considered in the previous separated improvements associated with diffuse and polarization functions (Δ*E*_G3largeXP_), the Hartree–Fock limit, the spin–orbit correction (*E*_SO_), *E*_ZPE_ consisting of the zero-point energy and other thermal effects (*E*_ZPE_), and a high level correction (Δ*E*_HLC_). A simplified version of the G4 method was developed by Curtiss et al.^32^ with lower-order perturbation theory, producing G4MP2, eq 4

4 $$E_{\mathrm{G4MP2}}=\mathrm{CCSD}\left(\mathrm{FC},T\right)/\mathrm{6‐31G}\left(d\right)+{\Delta E}_{\mathrm{MP2}}+{\Delta E}_{\mathrm{HF}}+E_{\mathrm{SO}}+E_{\mathrm{HLC}}+E_{\mathrm{ZPE}}$$

The theoretical enthalpies of combustion (Δ_c_H°_theo_) in the gas phase were obtained from eq 5, following the practical manual of thermochemical data by Gaussian:^33^

5 $$\Delta_{c}{H^{{}^{\circ}}}_{\mathrm{theo}}=\underset{\mathrm{products}}{\sum }\left(\varepsilon_{0}+H_{\mathrm{corr}}\right)-\underset{\mathrm{reagents}}{\sum }\left(\varepsilon_{0}+H_{\mathrm{corr}}\right)$$

where the sum of the electronic energies (ε_0_) with thermal enthalpies (*H*_corr_) of the products for complete combustion is written in eq 6:

6 $$\underset{\mathrm{products}}{\sum }\left(\varepsilon_{0}+H_{\mathrm{corr}}\right)=A_{n}\left(\varepsilon_{0}\left(CO_{2}\right)+H_{\mathrm{corr}}\left(CO_{2}\right)\right)+B_{n}\left(\varepsilon_{0}\left(H_{2}O\right)+H_{\mathrm{corr}}\left(H_{2}O\right)\right)$$

The sum of the electronic energies (ε_0_) with thermal enthalpies (*H*_corr_) of the reactants (C_*a*_H_*b*_O_*c*_ and O_2_) is written in eq 7:

7 $$\underset{\mathrm{reagents}}{\sum }\left(\varepsilon_{0}+H_{\mathrm{corr}}\right)=\left(\varepsilon_{0}\left(C_{a}H_{b}O_{c}\right)+H_{\mathrm{corr}}\left(C_{a}H_{b}O\right)\right)+D_{n}\left(\varepsilon_{0}\left(O_{2}\right)+H_{\mathrm{corr}}\left(O_{2}\right)\right)$$

Values *A*_*n*_, *B*_*n*_, and *D*_*n*_ correspond to the number of moles obtained in the stoichiometry of complete combustion. *H*_corr_ is the thermal correction energy for enthalpy (298.15 K).

## 3 Results and Discussion

Initially, to evaluate the performance of the theoretical methods and thus an accurate theoretical level for the calculations of theoretical enthalpies of combustion, a test set with 89 organic molecules^27^ (20 linear *n*-alkanes, 32 cycloalkanes, 18 alcohols, 13 aldehydes, and 6 ketones) was evaluated (Table 1).

**Table 1:** Combustion Enthalpies Calculated in the Present Work and Experimental Data Available in the Literature

Molecules	Exp.^a^ (kcal mol^–1^)	B3LYP (kcal mol^–1^)	M06-2X (kcal mol^–1^)	M08-HX (kcal mol^–1^)	G4-MP2 (kcal mol^–1^)	G4 (kcal mol^–1^)
Methane	–191.7	–179.6	–188.1	–190.1	–194.6	–192.3
Ethane	–341.2	–322.5	–334.6	–337.2	–346.5	–342.9
Propane	–488.5	–463.8	–478.5	–481.9	–495.6	–490.7
Butane	–635.0	–605.2	–622.4	–626.6	–644.6	–638.3
Pentane	–782.0	–746.6	–766.4	–771.4	–793.6	–786.0
Hexane	–928.9	–888.0	–910.4	–916.1	–942.6	–933.6
Heptane	–1075.8	–1029.5	–1054.5	–1061.0	–1091.5	–1081.2
Octane	–1222.7	–1170.9	–1198.5	–1205.7	–1240.5	–1228.8
Nonane	–1369.6	–1312.3	–1342.5	–1350.5	–1389.4	–1376.4
Decane	–1516.5	–1453.8	–1486.5	–1495.3	–1538.4	–1523.9
Undecane	–1663.4	–1595.2	–1630.5	–1640.0	–1687.3	–1671.5
Dodecane	–1810.4	–1736.6	–1774.5	–1784.8	–1836.3	–1819.1
Tridecane	–1957.3	–1878.0	–1918.5	–1929.6	—	—
Tetradecane	–2104.2	–2019.5	–2062.5	–2074.4	—	—
Pentadecane	–2251.1	–2160.9	–2206.5	–2219.1	—	—
Hexadecane	–2398.0	–2302.3	–2350.5	–2363.9	—	—
Heptadecane	–2544.9	–2443.7	–2494.5	–2508.7	—	—
Octadecane	–2691.8	–2585.2	–2638.5	–2653.4	—	—
Nonadecane	–2838.8	–2726.6	–2782.5	–2798.2	—	—
Eicosane	–2985.7	–2868.0	–2926.5	–2943.0	—	—
Cyclopropane	–468.3	–448.3	–457.8	–461.3	–475.2	–471.0
Cyclobutane	–614.1	–588.9	–603.6	–606.7	–624.0	–618.6
Cyclopentane	–740.9	–712.0	–727.9	–731.2	–752.8	–746.1
Cyclohexane	–881.5	–848.1	–865.2	–869.3	–895.4	–887.2
Cycloheptane	–1034.7	–996.3	–1015.0	–1019.8	–1050.6	–1041.2
Cyclooctane	–1182.6	–1150.4	–1171.5	–1176.3	–1211.9	–1201.2
Cyclodecane	–1478.4	–1430.3	–1455.6	–1461.4	–1504.0	–1490.6
Methylcyclopropane	–614.3	–587.7	–600.0	–604.1	–622.3	–616.7
Methylcyclobutane	–754.7	–727.7	–744.7	–748.6	–769.1	–762.3
Methylcyclopentane	–882.1	–852.0	–869.9	–873.3	–899.4	–891.2
Ethylcyclopentane	–1028.8	–995.6	–1015.0	–1019.3	–1049.9	–1040.4
n-Propylcyclopentane	–1174.8	–1137.2	–1159.0	–1164.2	–1198.9	–1188.0
Isopropylcyclopentane	–1173.6	–1137.0	–1157.7	–1162.9	–1196.9	–1186.0
Methylcyclohexane	–1025.9	–990.5	–1008.6	–1013.3	–1043.6	–1034.1
Ethylcyclohexane	–1172.4	–1133.0	–1153.1	–1158.3	–1192.9	–1182.1
n-Propylcyclohexane	–1318.2	–1274.5	–1297.1	–1303.0	–1341.8	–1329.6
Isopropylcyclohexane	–1317.0	–1275.9	–1296.5	–1302.2	–1340.9	–1328.7
*cis*-1,2-Dimethylcyclohexane	–1170.9	–1132.0	–1151.1	–1156.3	–1190.6	–1179.8
*trans*-1,2-Dimethylcyclohexane	–1171.4	–1133.1	–1152.1	–1157.2	–1191.5	–1180.7
*cis*-1,3-Dimethylcyclohexane	–1170.7	–1134.5	–1153.9	–1159.2	–1193.5	–1182.7
*trans*-1,3-Dimethylcyclohexane	–1171.2	–1130.7	–1150.4	–1155.7	–1189.8	–1180.9
*cis*-1,4-Dimethylcyclohexane	–1170.6	–1130.6	–1150.3	–1155.7	–1189.8	–1179.0
*trans*-1,4-Dimethylcyclohexane	–1171.2	–1133.0	–1152.2	–1157.3	–1191.8	–1180.9
1-Ethyl-3-methylcyclohexane	–1321.9	–1273.1	–1294.9	–1300.8	–1339.1	–1326.9
1,2,3-Trimethylcyclohexane	–1318.0	–1274.8	–1294.8	–1300.4	–1338.8	–1326.6
1,2,4-Trimethylcyclohexane	–1318.3	–1273.2	–1293.9	–1299.6	–1337.8	–1325.5
1,3,5-Trimethylcyclohexane	–1318.8	–1270.9	–1292.2	–1298.2	–1336.0	–1323.8
Butylcyclohexane	–1471.3	–1416.8	–1441.4	–1448.0	–1491.2	–1477.6
Bicyclo[2,2,2]octane	–1140.3	–1098.2	–1114.4	–1117.9	–1151.3	–1141.2
Adamantane	–1382.3	–1332.4	–1347.4	–1350.6	–1390.1	–1378.0
Decalin (*cis*)	–1423.5	–1380.6	–1400.0	–1404.8	–1447.0	–1434.2
Decalin (*trans*)	–1423.1	–1371.0	–1391.2	–1396.7	–1437.9	–1425.1
Propanone	–403.5	–384.7	–395.6	–397.9	–410.2	–406.2
2-Butanone	–550.4	–527.3	–540.4	–543.3	–560.3	–555.0
2-Pentanone	–697.4	–668.6	–684.4	–688.1	–708.2	–701.5
2-Hexanone	–844.2	–809.9	–828.3	–832.7	–857.2	–849.1
2-Heptanone	–991.1	–951.4	–972.3	–977.5	–1006.1	–996.7
2-Octanone	–1137.9	–1092.8	–1116.3	–1122.3	–1155.0	–1144.3
Methanol	–161.6	–153.0	–157.0	–159.0	–163.8	–162.0
Ethanol	–305.4	–290.7	–297.8	–300.5	–310.0	–306.7
Propanol	–451.8	–432.2	–441.9	–445.3	–459.3	–454.7
Butanol	–599.6	–573.7	–586.0	–590.2	–608.4	–602.5
Pentanol	–744.7	–715.1	–729.9	–734.9	–757.4	–750.1
Hexanol	–892.4	–856.5	–874.0	–879.7	–906.3	–897.7
Heptanol	–1040.6	–997.9	–1017.9	–1024.4	–1055.3	–1045.3
Octanol	–1187.2	–1139.4	–1161.9	–1169.2	–1204.2	–1192.9
Nonanol	–1331.9	–1280.8	–1305.9	–1314.0	–1353.2	–1340.4
Decanol	–1479.8	–1422.2	–1449.9	–1458.7	–1502.1	–1488.0
Undecanol	–1627.1	–1563.6	–1593.9	–1603.5	–1651.1	—
Dodecanol	–1774.0	–1705.1	–1737.9	–1748.3	–1800.0	—
Tridecanol	–1920.9	–1846.5	–1881.9	–1893.1	–1949.0	—
Tetradecanol	–2067.9	–1987.9	–2025.9	–2037.8	–2097.9	—
Pentadecanol	–2214.8	–2129.3	–2169.9	–2182.6	—	—
Hexadecanol	–2361.7	–2270.7	–2313.6	–2326.8	—	—
Heptadecanol	–2508.6	–2412.2	–2457.9	–2472.2	—	—
Octadecanol	–2655.5	–2553.6	–2601.9	–2616.9	—	—
Methanal	–124.1	–119.3	–123.8	–124.4	–126.6	–125.8
Ethanal	–263.9	–250.7	–259.0	–260.4	–267.9	–265.5
Propanal	–409.6	–392.7	–403.7	–405.8	–417.8	–414.0
Butanal	–558.4	–534.0	–547.7	–550.6	–566.9	–561.7
Pentanal	–704.8	–675.3	–691.6	–695.3	–715.7	–709.2
Hexanal	–851.7	–816.8	–835.6	–840.1	–864.7	–856.8
Heptanal	–999.8	–958.2	–979.6	–984.8	–1013.7	–1004.4
Octanal	–1145.5	–1099.6	–1123.6	–1129.6	–1162.6	–1152.0
Nonanal	–1292.4	–1241.0	–1267.6	–1274.4	–1311.5	–1299.6
Decanal	–1439.3	–1382.4	–1411.6	–1419.2	–1460.5	–1447.1
Undecanal	–1586.4	–1523.9	–1555.6	–1563.9	–1609.4	–1594.7
Dodecanal	–1732.6	–1665.3	–1699.6	–1708.7	–1758.4	—
Tridecanal	–1880.2	–1806.7	–1843.6	–1853.5	–1907.4	—
MRPE		3.95%	1.96%	1.39%	1.52%	0.59%
MAE		47.78	23.72	17.17	16.30	6.31
Std		5.04	13.37	9.91	6.99	3.71

a Ref 27.

In conjunction with the functional B3LYP, M06-2X, and M08-HX methods, the G4 and G4MP2 methods were also employed for the study. Composite methods present high computational costs and should be used according to processing capacity.^13^ In this study, G4 and G4MP2 calculations were only possible for structures containing up to 10 carbon atoms; consequently, certain systems were not included in the results because their molecular size prevented the convergence of the calculations.

To analyze the quality of the results obtained, the following were used:

Relative percent error (RPE) is calculated using eq 8:

8 $$RPE=\frac{\Delta_{c}H_{\mathrm{theo}}-\Delta_{c}H_{\exp}}{\Delta_{c}H_{\exp}}\times 100\%$$

Mean relative percent error (MRPE) corresponds to the average of the unsigned RPE values (eq 9):

9 $$MRPE=\frac{1}{N}\overset{N}{\underset{i=1}{\sum }}\left|RPE_{i}\right|$$

Mean absolute error (MAE) (eq 10):

10 $$MAE=\frac{1}{N}\overset{N}{\underset{i=1}{\sum }}\left|\Delta_{c}H_{\mathrm{theo}}-\Delta_{c}H_{\exp}\right|$$

Standard deviation of the errors (Std) (eq 11):

11 $$\mathrm{Std}=\sqrt{\frac{1}{N-1}\overset{N}{\underset{i=1}{\sum }}{\left(\Delta_{c}H_{\mathrm{theo}}-\Delta_{c}H_{\exp}\right)}^{2}}$$

The enthalpy of combustion values for the B3LYP, M06-2X, M08-HX, G4, and G4-MP2 methods are presented in Table 1.

Analysis of the results in Table 1 indicates that the composite method followed the trends of the theoretical levels^13^ with an MRPE of 0.59% for G4 and 1.52% for G4-MP2. Among the DFT methods, M08-HX (MRPE = 1.39%) and M06-2X (MRPE = 1.96%) demonstrated greater accuracy than B3LYP (MRPE = 3.95%). The M06-2X and M08-HX methods have comparable processing times, rendering M08-HX a viable contender for calculations that involve organic fuel molecules.

The mean absolute error (MAE = 6.31) and the lowest standard deviation of errors (Std = 3.71) indicate not only high average accuracy for the G4 method but also remarkable consistency in predictions, with errors homogeneously distributed. In contrast, the B3LYP functional exhibits the poorest performance (MAE = 47.78; Std = 25.04), reflecting its simpler class of functional (H-GGA) and lower Hartree–Fock exchange (25%) when compared to the M06-2X and M08-HX (Hm-GGA with >50% HF exchange), which represent progressive improvements, with M08-HX (MAE = 17.17; Std = 9.91) standing out. The G4-MP2 method, in turn, presents an MAE of 16.30 and the second lowest Std (6.99), evidencing concentrated and predictable errors. The Std/MAE ratio for G4, M08-HX, and M06-2X ranges between 0.58 and 0.59, suggesting symmetric error distributions without long tails, while for G4-MP2 this ratio is 0.43, reflecting a greater concentration associated with the negative bias. In summary, the data demonstrate that G4 is the most accurate and precise method for the studied data set, outperforming all others across every relevant metric, while M08-HX emerges as the best option among DFT functionals and G4-MP2 as a viable intermediate alternative, provided its bias is properly accounted for in future applications. Another point worth noting is that an increase in the number of atoms in the molecules increases the error associated with the calculation of the enthalpy of combustion and these results are more significant for the evaluated DFT methods (B3LYP, M06-2X, and M08-HX).

Figure 2 presents the relative percentage error (RPE) values for all of the calculated molecules. The composite methods underestimate the energy values, exhibiting predominantly positive errors with dispersion ranges of 1.5% and 1.9% for G4 and G4MP2, respectively. The DFT-based methods mostly resulted in negative errors, with dispersion ranges of 2.5%, 2.5%, and 3.6% for M08-HX, M06-2X, and B3LYP, respectively.

**Figure 2:**
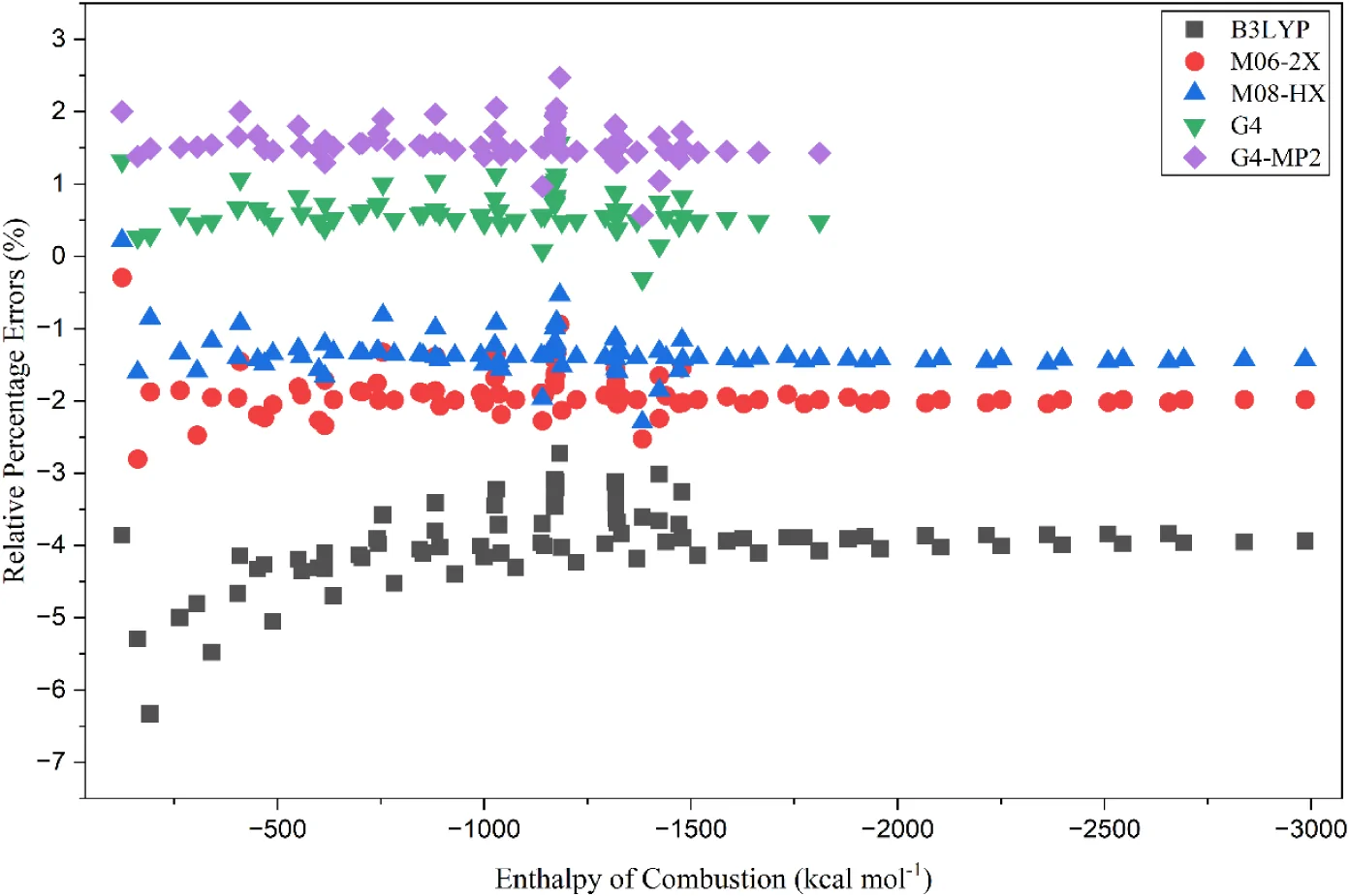
Relative percentage errors points for all molecules calculated.

When the molecular ensemble is analyzed, the errors follow a practically linear trend.

### 3.1 Effect of Empirical Correction on the Combustion Enthalpy

On the basis of the results presented for the test group (Table 1), an empirical correction can be employed to improve the computed combustion enthalpy of other groups of molecules for which experimental data are not available.

The empirical correction factor (EC) was obtained by dividing the experimental enthalpy of combustion by the corresponding theoretical value. For structurally similar organic molecules, these ratios are comparable; therefore, their average (eq 12) was calculated and applied as a multiplicative correction to the individual combustion enthalpy predicted by each method.

12 $$EC=\frac{1}{N}\overset{N}{\underset{i=1}{\sum }}\frac{\Delta_{c}H_{\exp,i}}{\Delta_{c}H_{\mathrm{theo},i}}$$

Table 2 presents the empirical correction factor, the relative percent error (RPE), and the |RPE| percentiles for all methods after applying the correction, demonstrating an improved accuracy.

**Table 2:** Relative Percent Error and Percentile Values for the Methods with Empirical Correction

Method	B3LYP	M06-2X	M08-HX	G4-MP2	G4
EC	1.0411	1.0200	1.0141	0.9851	0.9942
MRPE	0.24	0.08	0.06	0.08	0.12
Smaller |RPE|	0.00	0.00	0.00	0.00	0.00
10° Percentile	0.03	0.02	0.01	0.02	0.01
20° Percentile	0.09	0.05	0.03	0.05	0.04
30° Percentile	0.15	0.08	0.04	0.09	0.06
40° Percentile	0.20	0.10	0.06	0.13	0.07
50° Percentile	0.28	0.13	0.07	0.16	0.10
60° Percentile	0.41	0.18	0.10	0.21	0.17
70° Percentile	0.48	0.26	0.15	0.27	0.21
80° Percentile	0.63	0.34	0.22	0.42	0.27
90° Percentile	0.87	0.48	0.40	0.48	0.46
Larger |RPE|	2.56	1.64	1.62	0.95	0.98

Because the RPE values exhibit low dispersion, particularly for molecules with higher combustion energies, the application of EC (Figure 3) further reduces deviations, bringing the errors closer to zero.

**Figure 3:**
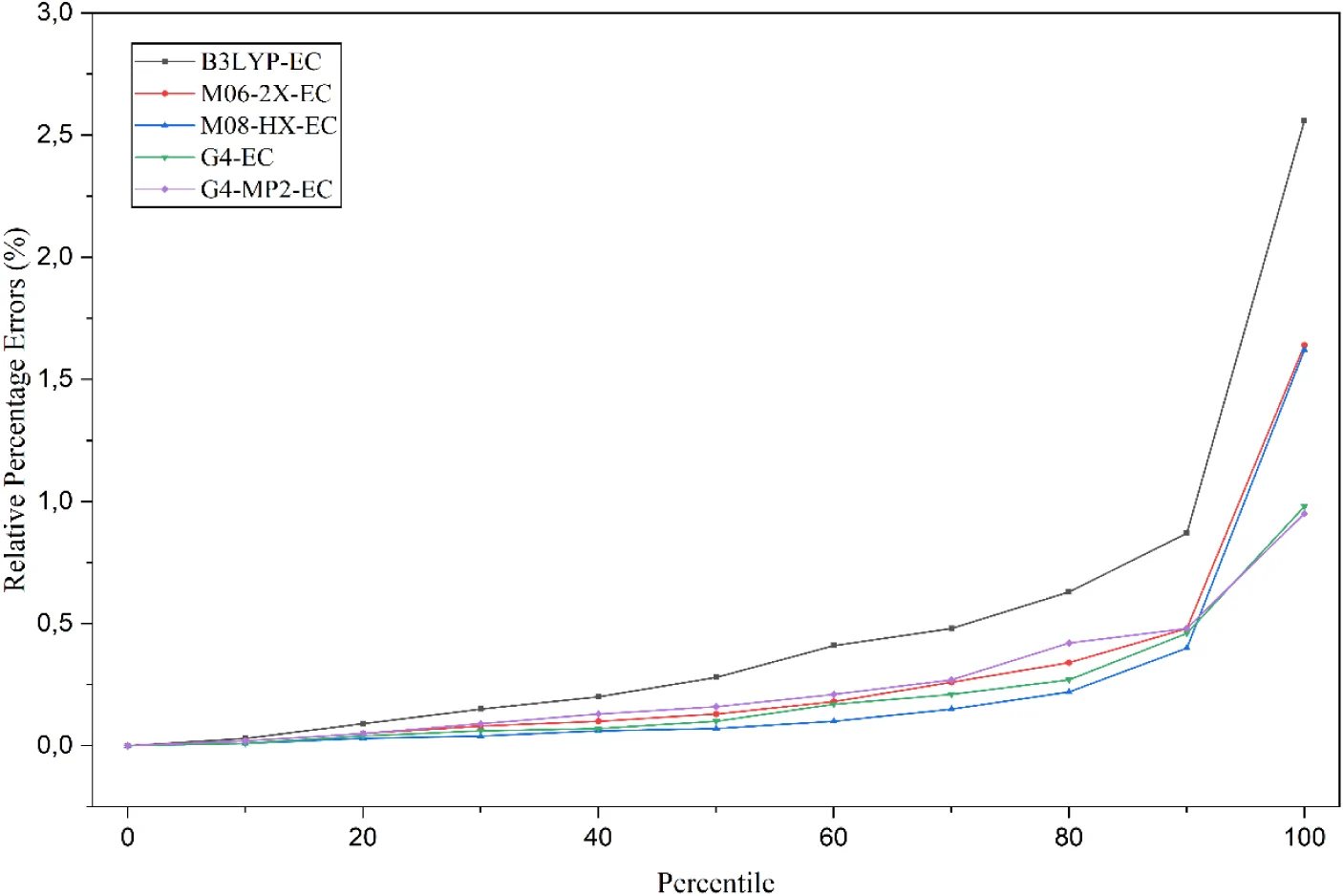
Relative percentage errors for all calculated molecules in the test group after empirical correction.

As shown in Table 2, all methods exhibited a reduction in MRPE after correction. The M08-HX-EC method achieved the lowest MRPE (0.06%) and a 90th percentile RPE of 0.40% (Table 2), outperforming G4-EC (MRPE = 0.12%).

Comparable improvements were observed for M06-2X-EC (MRPE = 0.08%) and G4-MP2-EC (MRPE = 0.08%), whereas B3LYP-EC showed the highest MRPE value (0.24%). These results demonstrate that implementing empirical corrections based on experimental data significantly enhances the predictive accuracy of the evaluated theoretical methods.

Studies in the literature highlight alternatives for accurately estimating enthalpies of formation using highly correlated methods such as CCSD­(T), G4, and G4MP2 and also employ empirical parameters to correct and improve the results.^15–17,34,35^

The RPE obtained for the test group was also analyzed by the size of the molecule (number of carbon atoms) and the class of the molecule (paraffins and cycloparaffins) in the group. Figure 4 presents the RPE values as a function of carbon number for the combustion enthalpies of 20 paraffins and 32 cycloparaffins.

**Figure 4:**
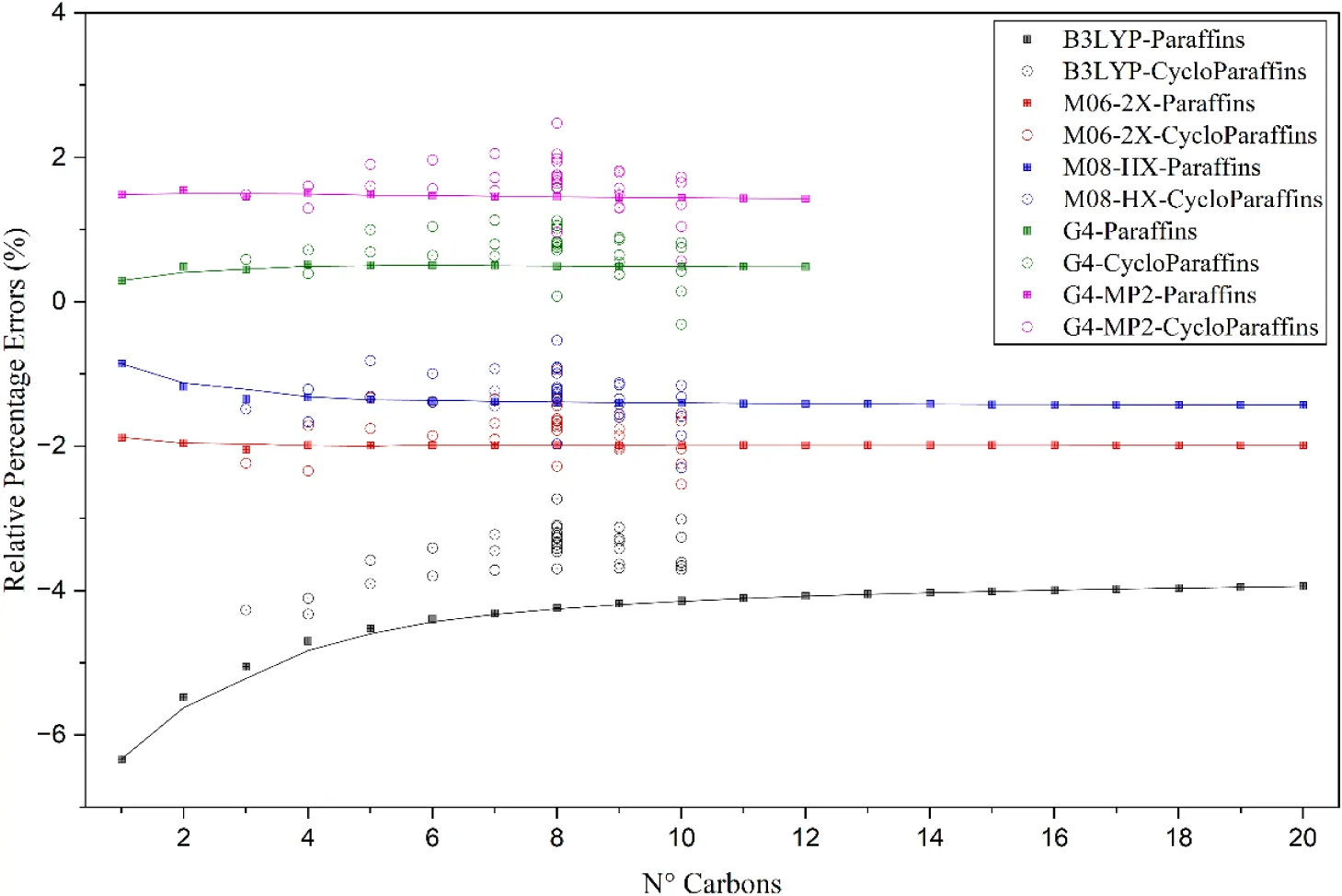
Relative percentage errors for paraffins and cycloparaffins in the test group.

Paraffins exhibit an approximately linear trend across all of the methods. An exception is observed for B3LYP in smaller molecules, which show higher RPE values for compounds containing one to five carbon atoms. Cycloparaffins display greater RPE variability, suggesting dependence on branch position, ring size, and carbon number.^31^ These measures reveal that the proposed methodology is robust and transferable for paraffins and larger cyclic alkanes but requires careful validation for small, strained rings.

The deviations in combustion enthalpy for the analyzed cycloparaffins from the test group are summarized in Table 3.

**Table 3:** Relative Percentage Error and Percentile Values for the Evaluated Methods Applied to Cycloparaffins in the Test Group

Method	B3LYP	M06-2X	M08-HX	G4-MP2	G4
MRPE	3.41	1.70	1.26	1.65	0.76
Smaller |RPE|	2.73	0.94	0.53	0.57	0.08
10° Percentile	3.11	1.34	0.92	1.30	0.14
20° Percentile	3.21	1.55	1.12	1.48	0.38
30° Percentile	3.27	1.66	1.27	1.60	0.64
40° Percentile	3.38	1.76	1.31	1.73	0.75
50° Percentile	3.41	1.85	1.39	1.76	0.80
60° Percentile	3.59	1.97	1.44	1.90	0.82
70° Percentile	3.67	2.03	1.56	1.96	0.89
80° Percentile	3.83	2.24	1.59	2.05	0.96
90° Percentile	4.16	2.05	1.85	2.47	1.05
Larger |RPE|	4.32	2.52	2.30	2.47	1.57

When comparing cycloparaffins with the complete test group, the data indicate lower mean percentage errors (MPEs) for the DFT-based methods and higher MPEs for the composite methods. New EC* values were calculated using empirical correction factors derived exclusively from cycloparaffins, and the results are presented in Table 4.

**Table 4:** Mean Relative Percentage Errors for the Methods with Empirical Correction Derived from Cycloparaffins

Method	B3LYP	M06-2X	M08-HX	G4-MP2	G4
EC	1.0411	1.0200	1.0141	0.9842	0.9951
MRPE	0.55	0.32	0.20	0.21	0.23
EC*	1.0354	1.0173	1.0128	0.9838	0.9925
MRPE*	0.22	0.18	0.17	0.16	0.15

As can be seen from the values in Table 4, the EC and EC* values are very close, and this small difference improves the MRPE values. For all methods, RPE values obtained with EC from the full test group ranged from 0.20% to 0.55% whereas RPE* values with EC* ranged from 0.15% to 0.22% (Table 4), indicating improved correction performance. B3LYP showed the largest reduction in mean RPE, decreasing from 0.55% to 0.22% suggesting greater effectiveness when applied to structurally similar molecules.

It is important to highlight that the composite methods require a substantially longer computational time. In contrast, the DFT methods exhibited no convergence issues and required significantly less computational time, as shown in Figure S1.

### 3.2 Combustion Enthalpies for SAF Candidates

After the combustion enthalpies for the test system were calculated using experimental values and the EC that best fits the DFT was determined, the next step was to evaluate some cycloparaffins. Among the various compounds present in aviation kerosene, cycloparaffins show high potential because they reduce the freezing temperature while maintaining high energy density and flash point.^9,10^

The Δ_c_H_theo_ values for the cycloparaffins proposed by Sacia et al.^9^ were calculated using DFT methods (Table 5).

**Table 5:** Theoretical Enthalpies of Combustion for Cycloparaffins-A (Figure 1)

Structure	Branching	Δ_c_H_B3LYP_ (kcal mol^–1^)	Δ_c_H_M06‑2X_ (kcal mol^–1^)	Δ_c_H_M08‑HX_ (kcal mol^–1^)
C_12_H_24_	R1(methyl)_R2(methyl)_R3(methyl)	–1704.7	–1728.0	–1735.0
C_15_H_30_	R1(methyl)_R2(methyl)_R3(butyl)	–2129.2	–2160.1	–2169.3
C_14_H_28_	R1(methyl)_R2(methyl)_R3(propyl)	–1987.7	–2016.1	–2024.5
C_13_H_26_	R1(methyl)_R2(methyl)_R3(ethyl)	–1846.3	–1872.2	–1879.8
C_15_H_30_	R1(methyl)_R2(butyl)_R3(methyl)	–2128.8	–2159.4	–2168.5
C_18_H_36_	R1(methyl)_R2(butyl)_R3(butyl)	–2553.2	–2591.1	–2602.4
C_17_H_34_	R1(methyl)_R2(butyl)_R3(propyl)	–2412.4	—	–2459.6
C_16_H_32_	R1(methyl)_R2(butyl)_R3(ethyl)	–2270.1	–2302.9	–2312.8
C_14_H_28_	R1(methyl)_R2(propyl)_R3(methyl)	–1987.4	–2015.4	–2023.7
C_17_H_34_	R1(methyl)_R2(propyl)_R3(butyl)	–2412.6	—	–2458.0
C_16_H_32_	R1(methyl)_R2(propyl)_R3(propyl)	–2270.4	–2303.1	–2312.9
C_15_H_30_	R1(methyl)_R2(propyl)_R3(ethyl)	–2129.0	–2159.1	–2168.3
C_13_H_26_	R1(methyl)_R2(ethyl)_R3(methyl)	–1845.9	–1871.3	–1878.9
C_16_H_32_	R1(methyl)_R2(ethyl)_R3(butyl)	–2270.4	–2303.0	–2312.9
C_15_H_30_	R1(methyl)_R2(ethyl)_R3(propyl)	–2128.9	–2159.0	–2168.2
C_14_H_28_	R1(methyl)_R2(ethyl)_R3(ethyl)	–1987.5	–2015.2	–2023.5
C_15_H_30_	R1(butyl)_R2(methyl)_R3(methyl)	–2133.3	–2162.6	—
C_18_H_36_	R1(butyl)_R2(methyl)_R3(butyl)	—	–2595.4	—
C_17_H_34_	R1(butyl)_R2(methyl)_R3(propyl)	–2416.4	–2450.7	—
C_16_H_32_	R1(butyl)_R2(methyl)_R3(ethyl)	–2274.1	–2306.3	—
C_18_H_36_	R1(butyl)_R2(butyl)_R3(methyl)	–2557.4	–2595.4	–2606.3
C_21_H_42_	R1(butyl)_R2(butyl)_R3(butyl)	–2978.3	–3024.1	–3037.6
C_20_H_40_	R1(butyl)_R2(butyl)_R3(propyl)	–2840.4	–2883.5	–2896.0
C_19_H_38_	R1(butyl)_R2(butyl)_R3(ethyl)	–2692.7	–2732.9	–2744.9
C_17_H_34_	R1(butyl)_R2(propyl)_R3(methyl)	–2416.0	–2451.4	–2461.6
C_20_H_40_	R1(butyl)_R2(propyl)_R3(butyl)	–2840.5	–2881.8	–2894.3
C_19_H_38_	R1(butyl)_R2(propyl)_R3(propyl)	–2698.4	–2739.4	–2751.1
C_18_H_36_	R1(butyl)_R2(propyl)_R3(ethyl)	–2557.7	–2593.9	–2604.8
C_16_H_32_	R1(butyl)_R2(ethyl)_R3(methyl)	–2274.6	–2306.0	–2315.7
C_19_H_38_	R1(butyl)_R2(ethyl)_R3(butyl)	–2699.0	–2739.5	–2751.1
C_18_H_36_	R1(butyl)_R2(ethyl)_R3(propyl)	–2557.7	–2594.1	–2605.2
C_17_H_34_	R1(butyl)_R2(ethyl)_R3(ethyl)	–2416.3	–2450.1	–2460.5
C_14_H_28_	R1(propyl)_R2(methyl)_R3(methyl)	–1991.9	–2018.7	–2026.8
C_17_H_34_	R1(propyl)_R2(methyl)_R3(butyl)	—	–2451.4	–2461.7
C_16_H_32_	R1(propyl)_R2(methyl)_R3(propyl)	–2275.0	–2306.8	–2316.3
C_15_H_30_	R1(propyl)_R2(methyl)_R3(ethyl)	–2132.7	–2162.3	–2171.3
C_17_H_34_	R1(propyl)_R2(butyl)_R3(methyl)	–2416.0	–2451.3	–2461.6
C_20_H_40_	R1(propyl)_R2(butyl)_R3(butyl)	–2840.6	–2882.1	–2894.7
C_19_H_38_	R1(propyl)_R2(butyl)_R3(propyl)	–2699.0	–2739.5	–2751.2
C_18_H_36_	R1(propyl)_R2(butyl)_R3(ethyl)	–2557.7	–2594.2	–2605.3
C_16_H_32_	R1(propyl)_R2(propyl)_R3(methyl)	–2274.6	–2306.1	–2315.7
C_19_H_38_	R1(propyl)_R2(propyl)_R3(butyl)	–2699.1	–2738.1	–2750.0
C_18_H_36_	R1(propyl)_R2(propyl)_R3(propyl)	–2557.6	–2595.5	–2606.5
C_17_H_34_	R1(propyl)_R2(propyl)_R3(ethyl)	–2416.3	–2450.2	–2460.6
C_15_H_30_	R1(propyl)_R2(ethyl)_R3(methyl)	–2133.2	–2162.2	–2171.0
C_18_H_36_	R1(propyl)_R2(ethyl)_R3(butyl)	–2557.7	–2594.2	–2604.6
C_17_H_34_	R1(propyl)_R2(ethyl)_R3(propyl)	–2416.2	–2450.2	–2460.5
C_16_H_32_	R1(propyl)_R2(ethyl)_R3(ethyl)	–2274.9	–2306.2	–2315.8
C_13_H_26_	R1(ethyl)_R2(methyl)_R3(methyl)	–1850.4	–1874.9	–1882.3
C_16_H_32_	R1(ethyl)_R2(methyl)_R3(butyl)	–2274.0	–2306.4	–2316.0
C_15_H_30_	R1(ethyl)_R2(methyl)_R3(propyl)	–2132.6	–2162.4	–2171.2
C_14_H_28_	R1(ethyl)_R2(methyl)_R3(ethyl)	–1992.0	–2019.0	–2027.2
C_16_H_32_	R1(ethyl)_R2(butyl)_R3(methyl)	–2274.6	–2306.3	–2316.0
C_19_H_38_	R1(ethyl)_R2(butyl)_R3(butyl)	–2698.9	–2739.5	–2751.2
C_18_H_36_	R1(ethyl)_R2(butyl)_R3(propyl)	–2557.5	–2595.5	–2606.4
C_17_H_34_	R1(ethyl)_R2(butyl)_R3(ethyl)	–2416.2	–2450.5	–2460.8
C_15_H_30_	R1(ethyl)_R2(propyl)_R3(methyl)	–2133.1	–2162.3	–2171.2
C_18_H_36_	R1(ethyl)_R2(propyl)_R3(butyl)	–2557.6	–2594.4	–2605.5
C_17_H_34_	R1(ethyl)_R2(propyl)_R3(propyl)	–2416.1	–2451.5	–2461.7
C_16_H_32_	R1(ethyl)_R2(propyl)_R3(ethyl)	–2274.8	–2306.4	–2316.0
C_14_H_28_	R1(ethyl)_R2(ethyl)_R3(methyl)	–1991.7	–2018.4	–2026.5
C_17_H_34_	R1(ethyl)_R2(ethyl)_R3(butyl)	—	–2448.7	–2459.3
C_16_H_32_	R1(ethyl)_R2(ethyl)_R3(propyl)	–2271.4	–2304.7	–2314.5
C_15_H_30_	R1(ethyl)_R2(ethyl)_R3(ethyl)	—	–2163.9	–2182.4

It is important to emphasize that molecules have multiple conformers and that there may be differences in the values of Δ_c_H°_theo_. The geometries of the molecules evaluated in Table 5 are provided in the Supporting Information.

The calculated Δ_c_H_theo_ values ranged from −1705 to −2978 kcal mol^–1^ for B3LYP, from −1728 to −3024 kcal mol^–1^ for M06-2X, and from −1735 to −3038 kcal mol^–1^ for M08-HX. The energy difference between M06-2X and M08-HX ranged from 7 to 18 kcal mol^–1^ whereas B3LYP differed from M08-HX by 30 to 59 kcal mol^–1^.

The EC* correction was applied to the combustion enthalpies of the cycloparaffin isomers, and the average values for each isomeric group are presented in Table 6.

**Table 6:** Theoretical Enthalpies of Combustion for Cycloparaffin Isomers

Structure	B3LYP (kcal mol^–1^)	B3LYP-EC* (kcal mol^–1^)	M06-2X (kcal mol^–1^)	M06-2X-EC* (kcal mol^–1^)	M08-HX (kcal mol^–1^)	M08-HX-EC* (kcal mol^–1^)
C_12_H_24_	–1704.7	–1765.0	–1728.0	–1757.9	–1735.0	–1757.2
C_13_H_26_	–1848.2	–1913.6	–1873.1	–1905.5	–1880.6	–1904.5
C_14_H_28_	–1955.1	–2025.3	–1985.9	–2020.5	–1995.8	–2021.0
C_15_H_30_	–2130.9	–2206.2	–2161.8	–2199.1	–2171.4	–2199.5
C_16_H_32_	–2273.3	–2353.7	–2306.2	–2346.1	–2315.9	–2345.5
C_17_H_34_	–2415.4	–2501.0	–2450.5	–2492.8	–2460.7	–2492.2
C_18_H_36_	–2557.2	–2647.8	–2594.4	–2639.4	–2605.4	–2638.7
C_19_H_38_	–2698.0	–2794.0	–2739.5	–2786.8	–2751.2	–2786.4
C_20_H_40_	–2840.5	–2941.1	–2882.5	–2932.3	–2895.0	–2932.1
C_21_H_42_	–2978.3	–3083.7	–3024.1	–3076.4	–3037.5	–3076.5

The results in Table 6 indicate that the M08-HX method yields deviations closest to those obtained with M06-2X, whereas B3LYP exhibits the largest deviations among the functionals. After applying EC* to the cycloparaffins-a proposed by Sacia et al. (2015), the Δ_c_H°_theo_ values calculated using M06-2X and M08-HX differ by less than 1 kcal mol^–1^. In contrast. B3LYP shows Δ_c_H°_theo_ deviations ranging from 6–10 kcal mol^–1^. The reduced differences in combustion enthalpy among the methods support the hypothesis that empirical correction based on structurally similar compounds significantly decreases deviations relative to the more accurate methods while preserving good precision.

## 4 Conclusion

The results obtained from the test group demonstrate that the G4 method provides the highest accuracy for predicting combustion enthalpy; however, it is also the most computationally demanding approach. The M08-HX results are comparable to those of the G4-MP2 composite method and, due to its lower computational cost, are more suitable for larger molecules. Comparing the computational times of the methods used, it can be inferred that the B3LYP method requires the least computational time, followed by M08-HX, M06-2X, G4-MP2, and G4. Regarding the accuracy of the methods, B3LYP is the least accurate, followed by M06-2X, M08-HX, G4-MP2, and G4.

Application of the empirical correction significantly improved the accuracy of the theoretical combustion enthalpy. Yielding errors were comparable to those of composite methods. The results for cycloparaffins further confirm the reliability of the functionals M06-2X and M08-HX, demonstrating high accuracy and precision in predicting the combustion enthalpy.

With reduced prediction errors, reliable evaluation of additional physicochemical properties—such as molecular stability and reaction kinetics—becomes feasible. These properties are essential for optimizing jet engine performance and reducing emissions of carbon monoxide, hydrocarbons, and particulate matter during combustion.

## Supplementary Material



## Notes

Accuracy-computational time relationship (Figure S1) and geometries of molecular structure (PDF)
