# Supplementary material for: Improved Combustion Enthalpy Predictions Obtained by DFT-Based Methods through Empirical Correction: A Study of Cycloparaffins for Sustainable Aviation Fuel
Source: ACS Omega. 2026 Oct 22;11(43):67565–75. doi: 10.1021/acsomega.6c08844 (PMC13737830; doi:10.1021/acsomega.6c08844)
Supplement: Supplementary file 1 [file ao6c08844_si_001.pdf]

Supplementary information

Improved combustion enthalpy predictions obtained by DFT-based methods through empirical correction: Study of cycloparaffins for sustainable aviation fuel

Thiago Soares Silva[a,b], Luiz Fernando de Araujo Ferrão[b], Douglas Henrique Pereira[a,b]*

[a] Postgraduate Program in Chemistry, Department of Chemistry, Federal University of Tocantins, Campus Gurupi-Badejós, P.O. Box 66, 77 402-970, Gurupi, Tocantins, Brazil.

[b] Department of Chemistry, Aeronautics Institute of Technology, Praça Marechal Eduardo Gomes, 50, Vila das Acácias, São José dos Campos, SP, 12228-900, Brazil

Corresponding author: *e-mail: douglas.pereira@gp.ita.br

ORCID: 0000-0002-7700-4769

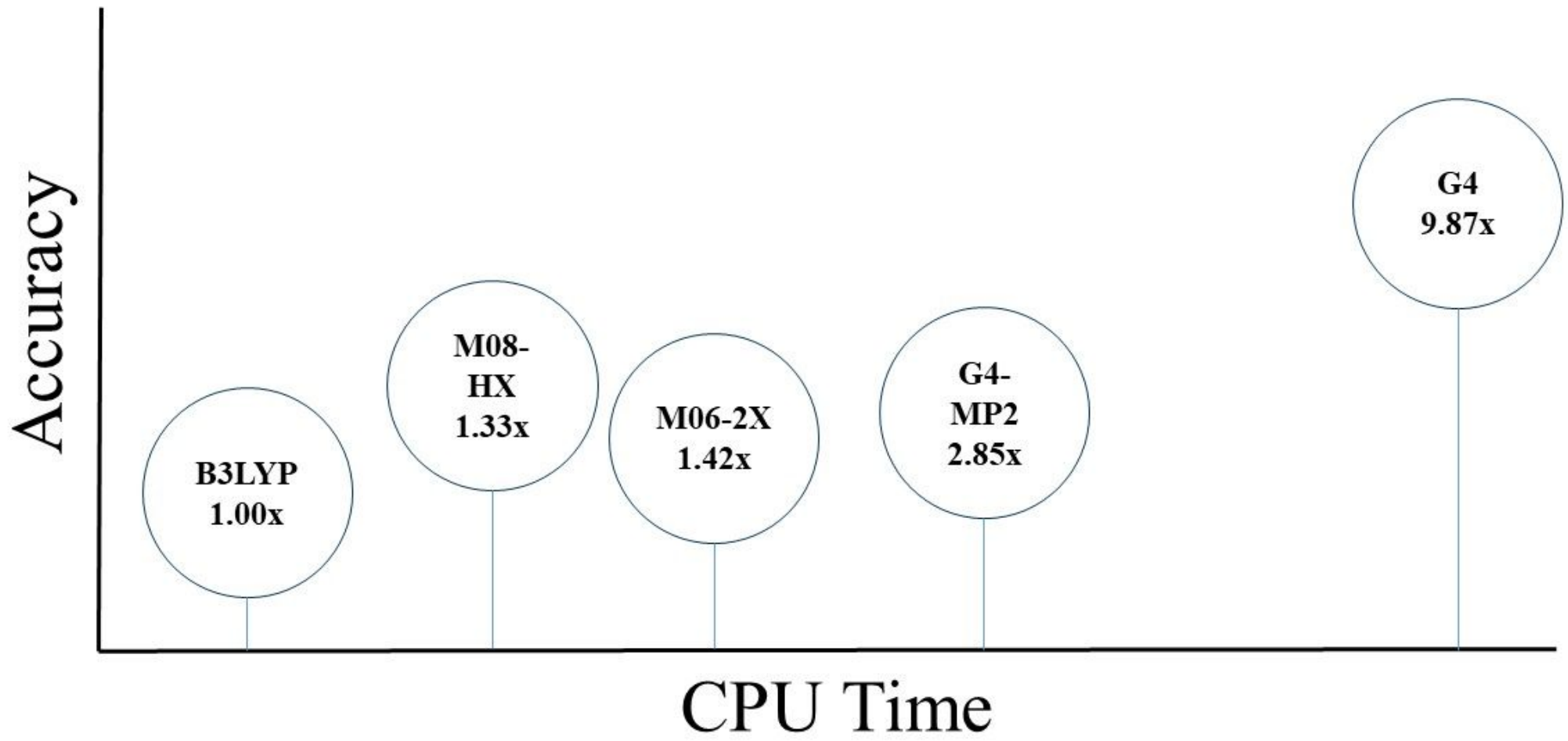

Figure S1. Accuracy-computational time relationship.

Geometries of molecular structure:

===

Cycloparaffins-a_R1-methyl_R2-methyl_R3-methyl

```
0 1
C   -2.98576244   6.02136534  -1.47832878
C   -2.37998904   4.65231244  -1.79693048
C   -1.10677537   4.31675723  -1.01617937
C   -1.41556694   4.41613102   0.48866937
C   -1.94572637   5.83390808   0.81832828
C   -3.25748046   6.09383226   0.03671075
H   -2.29426605   6.78844384  -1.75819181
H   -3.90025742   6.15516018  -2.01749283
H   -2.14236853   5.90099869   1.86796189
H   -1.21320154   6.56433347   0.54485779
C   -2.70228148   3.87278474  -3.08536525
H   -2.77000273   2.82837251  -2.86284127
H   -3.63466989   4.21357960  -3.48462796
C   -1.58516374   4.10594000  -4.11942840
H   -1.80909420   3.56432010  -5.01463957
H   -1.51744249   5.15035223  -4.34195239
H   -0.65277533   3.76514514  -3.72016569
C   -0.63151081   2.89347543  -1.36257874
H   -1.39718397   2.19025416  -1.10935065
H   -0.42323382   2.83210722  -2.41031661
H    0.25610163   2.66916132  -0.80874905
C   -3.80527472   7.48945818   0.38849155
H   -4.71364391   7.66219984  -0.14995598
H   -3.99816100   7.54236180   1.43963193
H   -3.08462956   8.23350103   0.12021783
C   -4.29259834   5.02160309   0.42459677
H   -5.20260370   5.19402344  -0.11118440
H   -3.91174691   4.05251912   0.17816536
C   -4.56613021   5.09597389   1.93828409
H   -4.94698165   6.06505786   2.18471550
H   -5.28533550   4.35098350   2.20778932
H   -3.65612485   4.92355354   2.47406527
H   -2.16097747   3.69428893   0.74983435
H   -0.52256992   4.22499580   1.04627465
H   -0.33975111   5.01797571  -1.27086827
H   -1.83486847   5.17867501  -2.55236812
```

===

Cycloparaffins-a_R1-methyl_R2-methyl_R3-butyl

```
0 1
C   -2.97819325   6.01571624  -1.44712290
C   -2.37449481   4.66554089  -1.84126620
C   -1.10777078   4.28038374  -1.07295192
C   -1.42666706   4.29257987   0.43302071
C   -1.95467851   5.69048245   0.84156434
```

C -3.26017145 6.00032491 0.06769667
H -2.28238912 6.79542689 -1.67692272
H -3.88851062 6.18405718 -1.98367073
H -2.15837608 5.69670446 1.89197787
H -1.21800561 6.43308463 0.61627552
C -2.69028185 3.96391106 -3.17528054
H -2.76279551 2.90850807 -3.01476172
H -3.61881648 4.33076901 -3.56016850
C -1.56530780 4.25327130 -4.18637101
H -1.78471811 3.76577521 -5.11325110
H -1.49279426 5.30867430 -4.34688987
H -0.63677319 3.88641341 -3.80148298
C -0.63455362 2.87815874 -1.49889887
H -1.40414888 2.16401524 -1.29244733
H -0.41921681 2.87749740 -2.54700654
H 0.24849857 2.61869037 -0.95319292
C -3.80603951 7.37488151 0.49690785
H -4.71011501 7.58203877 -0.03660255
H -4.00603595 7.36683956 1.54802001
H -3.08123952 8.13081525 0.27752427
C -4.30128577 4.91087302 0.38514838
H -5.20701687 5.11755911 -0.14573022
H -3.92175677 3.95654407 0.08495846
C -4.58506248 4.89746785 1.89871740
H -4.96459141 5.85179684 2.19890731
H -3.67933142 4.69078168 2.42959598
C -5.62617679 3.80801600 2.21616914
H -6.53190784 4.01470211 1.68529053
H -5.24664782 2.85368704 1.91597929
C -5.90995347 3.79461083 3.72973810
H -6.28948236 4.74893981 4.02992804
H -5.00422243 3.58792462 4.26061664
C -6.95106777 2.70515901 4.04718981
H -7.14823719 2.69584497 5.09882544
H -7.85679888 2.91184519 3.51631133
H -6.57153885 1.75083004 3.74699979
H -2.17611338 3.55931195 0.64644128
H -0.53815330 4.06599324 0.98447497
H -0.33682052 4.99260860 -1.28096988
H -1.82251805 5.23330394 -2.56089581

==

Cycloparaffins-a_R1-methyl_R2-methyl_R3-propyl
0 1
C -3.00906384 5.98819282 -1.48929167
C -2.32431738 4.65694977 -1.80817787
C -1.06720366 4.36848191 -0.98374143
C -1.43853612 4.42271387 0.50923141
C -2.05066044 5.80608441 0.84303540
C -3.34245661 6.01716145 0.01484834

H	-2.34611939	6.79352559	-1.72771174
H	-3.90751775	6.08788598	-2.06177857
H	-2.29085178	5.84260690	1.88508834
H	-1.34520002	6.57669061	0.61196900
C	-2.55748923	3.88840001	-3.12216528
H	-2.58193096	2.83770818	-2.92129463
H	-3.48947257	4.19114948	-3.55187442
C	-1.41412394	4.19632806	-4.10679646
H	-1.57613327	3.66233565	-5.01976212
H	-1.38968235	5.24702014	-4.30766714
H	-0.48214018	3.89357819	-3.67708715
C	-0.50894574	2.97726738	-1.33654362
H	-1.24805476	2.23261265	-1.12652554
H	-0.25749943	2.94698923	-2.37613863
H	0.36659679	2.78557685	-0.75209535
C	-3.97194033	7.37711019	0.36965790
H	-4.86623856	7.51598034	-0.20117133
H	-4.20773874	7.39961740	1.41311020
H	-3.27921218	8.16063242	0.14355837
C	-4.33739913	4.88817888	0.34209108
H	-5.23341843	5.02659564	-0.22614341
H	-3.89974194	3.94393504	0.09357572
C	-4.67264255	4.91898412	1.84484275
H	-5.11030071	5.86322742	2.09335803
H	-3.77662307	4.78056844	2.41307701
C	-5.66758332	3.79000000	2.17208596
H	-6.56360281	3.92841536	1.60385164
H	-5.22992515	2.84575662	1.92357071
C	-6.00282649	3.82080607	3.67483807
H	-6.44048495	4.76504944	3.92335314
H	-6.69411636	3.03638208	3.90220853
H	-5.10680692	3.68239091	4.24307248
H	-2.15680660	3.66029488	0.72764691
H	-0.55940974	4.26434392	1.09826110
H	-0.32659127	5.11123220	-1.19520140
H	-1.77712869	5.22421479	-2.53184544

==

Cycloparaffins-a_R1-methyl_R2-methyl_R3-ethyl

0 1

C	-2.94380983	6.06092963	-1.40309980
C	-2.43103104	4.68683459	-1.84092980
C	-1.15490797	4.22319665	-1.13396987
C	-1.40753391	4.22912975	0.38456305
C	-1.84162178	5.64663947	0.83408461
C	-3.16070582	6.03722145	0.12229228
H	-2.21767820	6.80604613	-1.65296825
H	-3.86603456	6.28559578	-1.89698909
H	-1.99926310	5.64750217	1.89240811
H	-1.07651690	6.35246523	0.58644645

C -2.84120358 4.02356922 -3.16887467
H -2.96331352 2.97115899 -3.01913450
H -3.76440913 4.44508238 -3.50787079
C -1.74690341 4.26832390 -4.22443250
H -2.03189353 3.80748373 -5.14709558
H -1.62479337 5.32073394 -4.37417257
H -0.82369791 3.84681083 -3.88543643
C -0.77646621 2.80454795 -1.59853156
H -1.57369822 2.12917507 -1.36789630
H -0.60681539 2.80858415 -2.65498901
H 0.11405829 2.49020014 -1.09548927
C -3.61297161 7.43205075 0.59293742
H -4.52675366 7.69509582 0.10231574
H -3.76765663 7.41846399 1.65161016
H -2.85874116 8.15172708 0.35189304
C -4.24418852 4.99982000 0.47080839
H -5.15953428 5.26244206 -0.01711767
H -3.92974175 4.03142104 0.14183010
C -4.46282596 4.97820961 1.99505597
H -4.77727269 5.94660859 2.32403423
H -3.54748023 4.71558754 2.48298206
C -5.54630868 3.94080818 2.34357207
H -5.69821915 3.92579319 3.40262718
H -6.46165440 4.20343024 1.85564600
H -5.23186191 2.97240919 2.01459383
H -2.18539165 3.53348468 0.62102711
H -0.50954732 3.94723508 0.89353793
H -0.35649471 4.89660601 -1.36625438
H -1.88086426 5.23550033 -2.57658080

==

Cycloparaffins-a_R1-methyl_R2-butyl_R3-methyl
0 1
C -3.35835057 6.16929354 -1.44517477
C -3.41746431 4.95169159 -2.37076937
C -2.10858221 4.16563832 -2.48118070
C -1.67923153 3.73002292 -1.06849028
C -1.53626237 4.97708550 -0.16061888
C -2.90175881 5.69988560 -0.05032453
H -2.65713883 6.87708387 -1.83534778
H -4.32269606 6.62768287 -1.37576993
H -1.22169830 4.66851553 0.81443702
H -0.80920106 5.64304718 -0.57628325
C -4.49757070 4.77434363 -3.45405970
H -4.78542992 3.74508040 -3.50557896
H -5.35075449 5.37143295 -3.20816997
C -3.93542827 5.21900298 -4.81709817
H -3.64756901 6.24826622 -4.76557891
H -3.08224451 4.62191361 -5.06298794
C -2.30685665 2.92779254 -3.37562190

H	-2.60899040	3.23919961	-4.35370205
H	-1.38718619	2.38497283	-3.44235269
C	-2.77093046	6.92084722	0.87904981
H	-3.71756443	7.41368063	0.95585688
H	-2.45699988	6.59819847	1.84974304
H	-2.04732686	7.59899304	0.47728363
C	-3.94111416	4.72164574	0.52793779
H	-4.03250169	3.87459611	-0.11940697
H	-3.62492906	4.39724553	1.49731464
C	-5.30400991	5.42941784	0.64271472
H	-5.62019505	5.75381802	-0.32666213
H	-6.02615939	4.74973177	1.04449446
H	-5.21262233	6.27646749	1.29005944
H	-3.06215548	2.29914348	-2.95227333
C	-5.01553463	5.04165507	-5.90038845
H	-5.86871840	5.63874442	-5.65449871
H	-5.30339390	4.01239183	-5.95190772
C	-4.45339215	5.48631446	-7.26342689
H	-4.16553290	6.51557770	-7.21190755
H	-3.60020835	4.88922516	-7.50931666
C	-5.53349854	5.30896656	-8.34671724
H	-6.38668229	5.90605592	-8.10082747
H	-5.14291903	5.61791819	-9.29376348
H	-5.82135783	4.27970333	-8.39823654
H	-3.12325751	5.64093743	-3.13449874
H	-1.35341330	4.79281667	-2.90693561
H	-2.41983618	3.07842836	-0.65396736
H	-0.74294804	3.21532177	-1.12642082

==

Cycloparaffins-a_R1-methyl_R2-butyl_R3-butyl

0 1

C	-3.63917185	6.12297035	-1.39019124
C	-3.41550891	4.95517324	-2.35469688
C	-1.98356614	4.41616899	-2.38775886
C	-1.58347991	4.00154022	-0.96021962
C	-1.72686401	5.21465169	-0.00793416
C	-3.20321299	5.68239943	0.02120347
H	-3.05002266	6.95911953	-1.70453543
H	-4.67225846	6.40112391	-1.38037213
H	-1.43009069	4.92513017	0.97847370
H	-1.10263795	6.01405157	-0.34829549
C	-4.36574651	4.63824452	-3.52469396
H	-4.46063982	3.57762926	-3.62957570
H	-5.32768809	5.06465083	-3.32960830
C	-3.79324739	5.23387354	-4.82475862
H	-3.69949212	6.29469935	-4.71974964
H	-2.83095170	4.80808307	-5.01823527
C	-1.89531654	3.20292033	-3.33211146
H	-2.17924471	3.49873141	-4.32045004

H	-0.89120342	2.83349491	-3.34501079
C	-3.35517331	6.86820025	0.99215543
H	-4.37737631	7.18396062	1.01225042
H	-3.05590659	6.56609004	1.97412132
H	-2.73794727	7.67854211	0.66467554
C	-4.08903052	4.51477735	0.49391450
H	-3.98579875	3.69189123	-0.18212279
H	-3.78575129	4.21118206	1.47421266
C	-5.55978742	4.96855720	0.52972611
H	-5.86333353	5.27121679	-0.45054424
H	-5.66296339	5.79212342	1.20521942
H	-2.55408980	2.43340697	-2.98707026
C	-4.73997154	4.91584045	-5.99726491
H	-5.70255604	5.34191035	-5.80466039
H	-4.83406498	3.85525006	-6.10202780
C	-4.16540776	5.51084307	-7.29633477
H	-4.07261231	6.57165959	-7.19183643
H	-3.20251410	5.08561894	-7.48806655
C	-5.11013911	5.19107613	-8.46977699
H	-6.07320555	5.61613964	-8.27852545
H	-4.71053419	5.60462349	-9.37221817
H	-5.20290365	4.13014842	-8.57416817
C	-6.44540308	3.80193079	1.00482435
H	-6.14269966	3.50090132	1.98608625
H	-6.34127924	2.97756665	0.33090568
C	-7.91653221	4.25542429	1.03772289
H	-8.02065077	5.08041931	1.71118944
H	-8.21891822	4.55591765	0.05634425
C	-8.80253650	3.08935641	1.51348278
H	-8.50049615	2.78948978	2.49516107
H	-9.82494638	3.40415225	1.53526627
H	-8.69764172	2.26413748	0.84038775
H	-2.22387839	3.21353539	-0.62243942
H	-0.56910712	3.66089146	-0.96209966
H	-1.32377021	5.18400905	-2.73484343
H	-3.19517253	5.71794762	-3.07162418

==

Cycloparaffins-a_R1-methyl_R2-butyl_R3-propyl

0 1

C	-3.93486714	5.71981716	-1.77778670
C	-3.49658515	4.45312736	-2.53634001
C	-2.15038957	3.75809360	-2.25968985
C	-1.70549751	3.96308829	-0.79928477
C	-1.83837311	5.43620111	-0.37200813
C	-3.34485913	5.75617895	-0.35618152
H	-3.57954736	6.56224400	-2.33362667
H	-5.00128196	5.76082001	-1.70057252
H	-1.44632597	5.55002304	0.61706822
H	-1.30688435	6.08604139	-1.03544776

C -4.15937164 4.20443059 -3.90398703
H -3.78547904 3.29270570 -4.32091982
H -5.21942075 4.13092013 -3.77841972
C -3.83477087 5.37351190 -4.85234097
H -4.20862668 6.28530791 -4.43554845
H -2.77474333 5.44712350 -4.97806065
C -2.25893481 2.24427954 -2.52065544
H -2.55371078 2.07757158 -3.53565183
H -1.30966156 1.78301084 -2.34458898
C -3.59371547 7.15605622 0.23552323
H -4.64537283 7.35201110 0.25805679
H -3.20178146 7.19759582 1.23029597
H -3.10684864 7.89119369 -0.37063670
C -4.03314994 4.69075464 0.51719757
H -3.86332305 3.72139252 0.09720820
H -3.62945441 4.72614320 1.50748568
C -5.54684628 4.96933786 0.56957165
H -5.95041253 4.93420050 -0.42078427
H -5.71683801 5.93857861 0.98980032
H -2.98888438 1.82045648 -1.86304456
C -4.49793718 5.12416662 -6.21972406
H -5.55793471 5.05054357 -6.09384807
H -4.12405759 4.21231480 -6.63647904
C -4.17370098 6.29305888 -7.16844767
H -4.54749984 7.20491587 -6.75168083
H -3.11370980 6.36665193 -7.29443856
C -4.83713671 6.04360725 -8.53568619
H -5.89713941 5.97000699 -8.40962793
H -4.61188346 6.85574755 -9.19491059
H -4.46335362 5.13173390 -8.95244212
C -6.23508608 3.90336684 1.44235017
H -5.83167869 3.93853627 2.43277519
H -6.06490010 2.93417631 1.02211536
C -7.74887628 4.18160580 1.49442107
H -8.22701032 3.44085379 2.10070891
H -7.91914995 5.15073453 1.91474166
H -8.15223642 4.14650346 0.50398099
H -2.33116929 3.38712000 -0.14989869
H -0.69092765 3.63536611 -0.70900300
H -2.76102739 5.11046309 -2.95078090
H -1.42719030 4.18879581 -2.92027456

==
Cycloparaffins-a_R1-methyl_R2-butyl_R3-ethyl
0 1
C -3.64161211 5.84197913 -1.72549471
C -3.38003251 4.51156773 -2.45372680
C -2.03085986 3.81285908 -2.19490650
C -1.71904893 3.75250591 -0.70450946
C -1.70072757 5.20040215 -0.16190747

C -3.12410638 5.76649334 -0.26208561
H -3.15823589 6.61967393 -2.27906337
H -4.69162842 6.03979991 -1.66863825
H -1.40239002 5.18997242 0.86560703
H -1.01433306 5.79948800 -0.72303014
C -4.21099024 4.20389351 -3.71326873
H -3.96020769 3.22926113 -4.07672398
H -5.25251327 4.23685152 -3.47027991
C -3.90387288 5.25152638 -4.79942906
H -4.15465547 6.22615877 -4.43597385
H -2.86234984 5.21856839 -5.04241787
C -2.06496810 2.39691382 -2.79950191
H -2.25900648 2.46211421 -3.84973902
H -1.12136563 1.91823844 -2.64017753
C -3.17737019 7.18006050 0.34667614
H -4.18096860 7.54877512 0.30496578
H -2.85345723 7.14195915 1.36575841
H -2.53469269 7.83160104 -0.20772496
C -4.03804170 4.81431168 0.53143329
H -3.99846900 3.83733015 0.09687480
H -3.70667446 4.76632725 1.54769771
C -5.48585315 5.33731468 0.48773064
H -5.81722043 5.38529913 -0.52853376
H -5.52542576 6.31429618 0.92228908
H -2.83823074 1.82658269 -2.32866522
C -4.73483054 4.94385212 -6.05897097
H -5.77635356 4.97681005 -5.81598220
H -4.48404788 3.96921967 -6.42242621
C -4.42771328 5.99148506 -7.14513127
H -4.67849588 6.96611753 -6.78167604
H -3.38619027 5.95852707 -7.38812004
C -5.25867103 5.68381078 -8.40467320
H -6.30019406 5.71676871 -8.16168441
H -5.04528439 6.41171155 -9.15934302
H -5.00788840 4.70917837 -8.76812843
C -6.39978850 4.38513302 1.28124960
H -6.06842125 4.33714856 2.29751400
H -7.40573543 4.74851826 1.25088483
H -6.36021585 3.40815148 0.84669108
H -2.68964173 4.95667552 -3.13939403
H -1.25625482 4.38649257 -2.65948329
H -2.45905728 3.17928224 -0.18614448
H -0.76004105 3.29872968 -0.56561755

==

Cycloparaffins-a_R1-methyl_R2-propyl_R3-methyl
0 1
C -3.13295376 6.14012029 -1.47678548
C -3.48058020 4.93786180 -2.35798042
C -2.31552520 3.98248271 -2.62842507

C -1.75642791 3.49869921 -1.27818033
C -1.32504570 4.71880226 -0.42665583
C -2.55407213 5.61911704 -0.14716955
H -2.40484930 6.74561582 -1.97492105
H -4.00900266 6.72431438 -1.28663933
H -0.92231517 4.37460754 0.50298825
H -0.58064742 5.27927160 -0.95262671
C -4.71409257 4.90308696 -3.27930447
H -5.14027909 3.92169330 -3.26783453
H -5.43821929 5.61047095 -2.93268012
C -4.29201393 5.26268111 -4.71601656
H -3.86582735 6.24407476 -4.72748656
H -3.56788726 4.55529709 -5.06264091
C -2.79810577 2.77900435 -3.45930165
H -3.18867875 3.12432678 -4.39370264
H -1.97673331 2.11716680 -3.63879622
C -2.13509237 6.81507469 0.72789687
H -2.98816469 7.43104715 0.92217896
H -1.73545514 6.45699693 1.65362383
H -1.39054796 7.38813757 0.21588852
C -3.62389664 4.79185667 0.58952294
H -3.91553719 3.96222742 -0.02003520
H -3.22246111 4.43173490 1.51367761
C -4.85174382 5.67694826 0.87343167
H -5.25317933 6.03707012 -0.05072297
H -5.59506349 5.10216349 1.38528938
H -4.56010323 6.50657744 1.48298987
H -3.56420475 2.25933646 -2.92270345
C -5.52552634 5.22790623 -5.63734055
H -6.24965304 5.93529021 -5.29071614
H -5.95171287 4.24651257 -5.62587053
C -5.10344770 5.58750041 -7.07405275
H -5.96049848 5.56333869 -7.71419353
H -4.67726114 6.56889405 -7.08552269
H -4.37932104 4.88011641 -7.42067710
H -2.51237052 2.95428327 -0.75181348
H -0.91380274 2.86252497 -1.45186158
H -1.55008172 4.50070106 -3.16735553
H -3.20608706 5.57823582 -3.17006084

==

Cycloparaffins-a_R1-methyl_R2-propyl_R3-butyl
0 1
C 0.50898029 2.67390779 1.76188502
C 1.58942849 2.81526581 2.85034655
C 2.01569667 1.63189965 3.73905066
C 1.85046096 0.29045350 3.00017102
C 0.47794892 0.19371437 2.30941745
C 0.47505606 1.24673437 1.18539346
H -0.43286804 2.89744244 2.21785377

H 0.69355753 3.35670552 0.95900439
H 0.37193165 -0.77936783 1.87722824
H -0.32135649 0.35756967 3.00160140
C 1.84989197 4.23663978 3.38274745
H 2.62670116 4.20536392 4.11792772
H 2.14899905 4.87091971 2.57457042
C 0.56194433 4.79021012 4.02014120
H -0.21493190 4.82140170 3.28503181
H 0.26288982 4.15604536 4.82841583
C 3.49151293 1.77189782 4.15603560
H 3.62701835 2.69231024 4.68459246
H 3.76223154 0.95342882 4.78984218
C -0.78833561 1.10562962 0.31616428
H -0.76267741 1.83029706 -0.47065146
H -0.82338782 0.12271378 -0.10521614
H -1.65674084 1.26582075 0.92041239
C 1.72945839 1.00636913 0.32502600
H 1.75821258 1.72086269 -0.47094040
H 2.60405725 1.11327117 0.93212571
C 1.68392463 -0.41593102 -0.26355949
H 0.80942936 -0.52290156 -0.87079942
H 1.65508689 -1.13060848 0.53224930
H 4.11069518 1.76722623 3.28340167
C 0.82256063 6.21166826 4.55222133
H 1.12167986 6.84575992 3.74392994
H 1.59938996 6.18047642 5.28738772
C -0.46536514 6.76539982 5.18953306
H -0.28431003 7.75306665 5.55916866
H -1.24220996 6.79654105 4.45438503
H -0.76445573 6.13131457 5.99785007
C 2.93873378 -0.65551876 -1.12362633
H 3.81312416 -0.54851977 -0.51623557
H 2.96753587 0.05926241 -1.91935102
C 2.89376563 -2.07768518 -1.71260920
H 2.86488118 -2.79253092 -0.91696132
H 2.01945979 -2.18469052 -2.32011538
C 4.14879642 -2.31695902 -2.57240660
H 5.02303940 -2.20996220 -1.96483169
H 4.11767026 -3.30507707 -2.98174526
H 4.17770185 -1.60206801 -3.36802833
H 2.59583874 0.20758888 2.23699792
H 1.97037173 -0.50042582 3.71081667
H 1.39102587 1.64393516 4.60770501
H 0.76249120 2.65995895 3.51137132

==

Cycloparaffins-a_R1-methyl_R2-propyl_R3-propyl
0 1
C -3.29194513 5.97528663 -1.50383855
C -3.45329819 4.76151544 -2.42119348

C	-2.15449929	3.95395710	-2.66226550
C	-1.63924465	3.49839472	-1.27591377
C	-1.39018854	4.74278356	-0.37240195
C	-2.74123990	5.49380603	-0.15573274
H	-2.58392514	6.65655765	-1.95827956
H	-4.22053020	6.48738167	-1.38791589
H	-0.98531726	4.43291313	0.56459079
H	-0.70182715	5.40432522	-0.86203557
C	-4.65094263	4.62626789	-3.38317867
H	-4.91035331	3.59586248	-3.49050891
H	-5.48829573	5.14955466	-3.00465962
C	-4.26321499	5.20807680	-4.77111830
H	-4.01567517	6.24167309	-4.66350221
H	-3.43431535	4.67463342	-5.17227531
C	-2.42262540	2.71782407	-3.54451759
H	-2.81921217	3.02289790	-4.48199880
H	-1.52789132	2.17544634	-3.69057649
C	-2.52556240	6.70578462	0.79201588
H	-3.45611171	7.18616902	0.94110941
H	-2.12905698	6.35844701	1.71929224
H	-1.82753717	7.38249687	0.33349836
C	-3.77013570	4.52710509	0.48086325
H	-3.91714886	3.67352146	-0.15103652
H	-3.40668802	4.20442240	1.43365810
C	-5.11788038	5.27835020	0.67535084
H	-5.46419735	5.62591013	-0.28444800
H	-4.97368329	6.10923580	1.33539699
H	-3.13796934	2.09275243	-3.05252705
C	-5.48064701	5.07478599	-5.72620986
H	-6.30979550	5.58484491	-5.29826615
H	-5.72074314	4.04746683	-5.85519947
C	-5.13542931	5.69759183	-7.08841499
H	-5.97650062	5.60714103	-7.76462776
H	-4.89641566	6.73993714	-6.94685818
H	-4.27803671	5.19010808	-7.49284957
C	-6.18233094	4.32515094	1.27687123
H	-5.86675595	3.97736517	2.23298923
H	-6.31949891	3.48917060	0.60269251
C	-7.51187645	5.07098412	1.42629263
H	-8.23841527	4.40604389	1.82777503
H	-7.39134066	5.90719071	2.08755948
H	-7.83709946	5.43169097	0.47332949
H	-3.23286163	5.45958448	-3.20334240
H	-1.44540601	4.57036812	-3.17365036
H	-2.39752679	2.87063712	-0.82261761
H	-0.72524183	2.94310877	-1.39129576

==

Cycloparaffins-a_R1-methyl_R2-propyl_R3-ethyl

0 1

C -3.29535316 6.08862925 -1.45911481
C -3.48141713 4.88882456 -2.39111443
C -2.21925529 4.05402551 -2.62198387
C -1.69000461 3.58002264 -1.25632936
C -1.42076387 4.80626017 -0.34875741
C -2.74184854 5.58007676 -0.11396280
H -2.60167435 6.77445818 -1.89882880
H -4.23114755 6.58322585 -1.30238870
H -1.03764573 4.47048959 0.59218821
H -0.70518638 5.44968389 -0.81657655
C -4.65542689 4.77071540 -3.38073626
H -4.98781935 3.75441714 -3.42014585
H -5.46005536 5.39679690 -3.05593633
C -4.19297661 5.21422660 -4.78110679
H -3.86058414 6.23052485 -4.74169727
H -3.38834818 4.58814507 -5.10590668
C -2.54205309 2.83894944 -3.51136098
H -2.91293667 3.17711595 -4.45634182
H -1.65391151 2.26170189 -3.66265464
C -2.48393015 6.78037622 0.81574645
H -3.40007858 7.30857894 0.97876169
H -2.10237826 6.43063195 1.75222767
H -1.77013059 7.43589208 0.36221680
C -3.76719012 4.63432693 0.53859512
H -3.94696278 3.80167654 -0.10891945
H -3.38357528 4.28276501 1.47355175
C -5.08591203 5.39295742 0.77745134
H -5.46952686 5.74451930 -0.15750528
H -4.90613930 6.22560785 1.42496587
H -3.28359269 2.23378931 -3.03303034
C -5.36698629 5.09611744 -5.77072858
H -6.17161479 5.72219893 -5.44592872
H -5.69937870 4.07981919 -5.81013809
C -4.90453596 5.53962865 -7.17109911
H -5.72024400 5.45756577 -7.85869352
H -4.57214350 6.55592692 -7.13168959
H -4.09990747 4.91354713 -7.49589895
C -6.11125369 4.44720762 1.43000928
H -5.72763885 4.09564574 2.36496591
H -7.02750853 4.97430805 1.59596781
H -6.29102642 3.61455719 0.78249476
H -1.47810747 4.65775870 -3.10271888
H -3.22525705 5.57784918 -3.16862783
H -2.41858397 2.95150040 -0.78832089
H -0.78344737 3.03008230 -1.39992460

==
Cycloparaffins-a_R1-methyl_R2-ethyl_R3-methyl
0 1
C -3.12834712 6.12151060 -1.53004411

C -3.60492805 4.90886175 -2.33324251
C -2.51229412 3.88932121 -2.66480070
C -1.86209889 3.42423553 -1.34922531
C -1.30388930 4.64962332 -0.58328627
C -2.46193377 5.61761651 -0.23528123
H -2.41875036 6.67381131 -2.10998926
H -3.95721049 6.75374333 -1.28886515
H -0.83903021 4.31745399 0.32140681
H -0.58252385 5.15525233 -1.19063395
C -4.91324890 4.90350234 -3.14556501
H -5.38131254 3.94498380 -3.06154930
H -5.57184801 5.65653095 -2.76596638
C -4.60001773 5.19389479 -4.62514551
H -4.13195409 6.15241333 -4.70916121
H -3.94141861 4.44086618 -5.00474413
C -3.11892729 2.68371469 -3.40649410
H -3.57204299 3.01625806 -4.31698907
H -2.34711801 1.97699548 -3.62955998
C -1.91515173 6.82029227 0.55604703
H -2.71933918 7.48326587 0.79822169
H -1.45434231 6.47428421 1.45761960
H -1.19186670 7.33895210 -0.03788121
C -3.50130097 4.86856705 0.61926000
H -3.88180960 4.03424160 0.06788514
H -3.03893018 4.52037834 1.51919215
C -4.65848848 5.82145384 0.97213933
H -5.12085927 6.16964255 0.07220718
H -5.38064621 5.30101038 1.56587902
H -4.27797986 6.65577929 1.52351419
H -3.85911276 2.22022886 -2.78826570
C -5.90833858 5.18853538 -5.43746801
H -6.56693769 5.94156400 -5.05786938
H -5.69070393 5.39030157 -6.46548822
H -6.37640222 4.23001685 -5.35345230
H -3.37213926 5.50760540 -3.18893838
H -1.77302759 4.35131413 -3.28524210
H -2.59418579 2.93520790 -0.74111287
H -1.06746504 2.74229306 -1.56925147

===
Cycloparaffins-a_R1-methyl_R2-ethyl_R3-butyl
0 1
C -3.55366633 6.03642397 -1.46205899
C -3.50210452 4.84649409 -2.42340310
C -2.12220962 4.20052192 -2.57122917
C -1.63212680 3.76976078 -1.17693339
C -1.60347903 4.99799123 -0.23328888
C -3.03331511 5.57533417 -0.08690166
H -2.93274826 6.82259694 -1.83794829
H -4.55831163 6.39262414 -1.36873566

H -1.24629426 4.69466034 0.72864116
H -0.95296912 5.74592544 -0.63619249
C -4.57329545 4.59225484 -3.50020964
H -4.75652385 3.54107117 -3.57983573
H -5.47897213 5.09251077 -3.22745745
C -4.07727722 5.13093959 -4.85497333
H -3.89404875 6.18212321 -4.77534717
H -3.17160060 4.63068364 -5.12772554
C -2.20662450 2.97546824 -3.50059322
H -2.55177051 3.28296677 -4.46559009
H -1.23786910 2.53070693 -3.59333143
C -3.01382267 6.77575518 0.87757195
H -4.00422337 7.16778600 0.97915059
H -2.65590674 6.45848635 1.83472226
H -2.36779444 7.53505196 0.48896428
C -3.96080672 4.48052108 0.47233320
H -3.97498586 3.64773358 -0.19935420
H -3.60048883 4.16177687 1.42809110
C -5.38647930 5.04305271 0.62271894
H -5.74679718 5.36179690 -0.33303897
H -5.37230012 5.87584021 1.29440630
H -2.88888577 2.26155738 -3.08858513
C -5.14846815 4.87670047 -5.93177990
H -6.05414481 5.37695644 -5.65902771
H -4.80383205 5.25098149 -6.87307668
H -5.33169662 3.82551685 -6.01140611
C -6.31397096 3.94823969 1.18195386
H -5.95365311 3.62949555 2.13771179
H -6.32815009 3.11545215 0.51026656
C -7.73964358 4.51077133 1.33233945
H -7.72546450 5.34355887 2.00402675
H -8.09996141 4.82951545 0.37658151
C -8.66713519 3.41595830 1.89157435
H -8.30681741 3.09721425 2.84733233
H -9.65769995 3.80680813 1.99606297
H -8.68131421 2.58317072 1.21988707
H -3.28923614 5.58387355 -3.16896237
H -1.43996153 4.91302702 -2.98568553
H -2.29757445 3.03477590 -0.77459434
H -0.64956273 3.35438777 -1.26019872

==
Cycloparaffins-a_R1-methyl_R2-ethyl_R3-propyl
0 1
C -3.54481058 6.06371158 -1.46160582
C -3.50591475 4.85849740 -2.40432533
C -2.13139955 4.20012994 -2.54751365
C -1.63872764 3.78769630 -1.14859623
C -1.59720991 5.03033633 -0.22449781
C -3.02217207 5.62043045 -0.08146899

H -2.91972000 6.83929125 -1.85229481
H -4.54643205 6.42872901 -1.36986355
H -1.23828446 4.73950997 0.74064086
H -0.94291959 5.76704104 -0.64171528
C -4.58337020 4.59529900 -3.47269619
H -4.77460328 3.54437488 -3.53505279
H -5.48423322 5.10643603 -3.20421018
C -4.08902375 5.10899044 -4.83773931
H -3.89779066 6.15991460 -4.77538271
H -3.18816071 4.59785342 -5.10622533
C -2.22860022 2.96130138 -3.45715963
H -2.57546942 3.25615782 -4.42547852
H -1.26351214 2.50800999 -3.54678080
C -2.98992671 6.83565950 0.86392964
H -3.97700825 7.23652667 0.96330588
H -2.63039176 6.53081684 1.82450528
H -2.33997587 7.58398244 0.46084563
C -3.95532428 4.54139382 0.49862311
H -3.97836146 3.69829749 -0.15982796
H -3.59340400 4.23503661 1.45782013
C -5.37621478 5.11671092 0.64586593
H -5.73813500 5.42306817 -0.31333111
H -5.35317761 5.95980725 1.30431705
H -2.91435462 2.25899180 -3.03124629
C -5.16647922 4.84579202 -5.90611018
H -6.06734220 5.35692913 -5.63762415
H -4.82300474 5.20270745 -6.85454926
H -5.35771238 3.79486785 -5.96846677
C -6.30936704 4.03767429 1.22595802
H -5.94744685 3.73131710 2.18515509
H -6.33240415 3.19457795 0.56750691
C -7.73025757 4.61299129 1.37320076
H -8.37861655 3.86327096 1.77625169
H -7.70722051 5.45608758 2.03165192
H -8.09217777 4.91934855 0.41400367
H -2.30786287 3.06404020 -0.73208702
H -0.65957752 3.36383561 -1.22926310
H -1.44567880 4.90099562 -2.97585271
H -3.29074189 5.58249928 -3.16223159

==
Cycloparaffins-a_R1-methyl_R2-ethyl_R3-ethyl
0 1
C -3.47805936 6.09853489 -1.46875716
C -3.51439751 4.86982407 -2.38074595
C -2.17178664 4.15323161 -2.54546969
C -1.65529452 3.75593263 -1.15077024
C -1.53770039 5.01832506 -0.26061701
C -2.93340550 5.66875080 -0.09287027
H -2.83439147 6.83839471 -1.89677489

H	-4.46134271	6.50579865	-1.35834826
H	-1.16261268	4.73718036	0.70123891
H	-0.86718035	5.71752091	-0.71496051
C	-4.63209310	4.62398889	-3.41124180
H	-4.86652834	3.58041184	-3.44102503
H	-5.50380509	5.17750972	-3.13082624
C	-4.15776370	5.08346828	-4.80246079
H	-3.92332849	6.12704534	-4.77267752
H	-3.28605171	4.52994745	-5.08287633
C	-2.34442447	2.89724865	-3.41971395
H	-2.70740353	3.18185439	-4.38519092
H	-1.40106429	2.40333523	-3.52469546
C	-2.82553753	6.90466270	0.81953663
H	-3.79265648	7.34733826	0.93623877
H	-2.45052914	6.60938016	1.77717789
H	-2.15847977	7.61598772	0.37913825
C	-3.89126252	4.64287210	0.54091434
H	-3.96683839	3.78539027	-0.09462731
H	-3.51397177	4.34594637	1.49715006
C	-5.28336338	5.27847718	0.71313924
H	-5.66065412	5.57540290	-0.24309652
H	-5.20778750	6.13595901	1.34868092
H	-3.04475525	2.23389286	-2.95668492
C	-5.27545920	4.83763313	-5.83295670
H	-6.14717123	5.39115393	-5.55254118
H	-4.94589260	5.15688185	-6.79958285
H	-5.50989441	3.79405607	-5.86273999
C	-6.24122046	4.25259855	1.34692384
H	-5.86392978	3.95567285	2.30315964
H	-7.20845936	4.69422029	1.46658651
H	-6.31679632	3.39511671	0.71138221
H	-3.29289071	5.56562652	-3.16285445
H	-1.47161730	4.81508652	-3.01088451
H	-2.34014542	3.07035196	-0.69705405
H	-0.69646302	3.29107102	-1.24795974

==

Cycloparaffins-a_R1-butyl_R2-methyl_R3-methyl

0 1

C	-3.04960621	5.84894211	-1.51178050
C	-2.26307992	4.55527319	-1.73666122
C	-1.00110301	4.41489083	-0.88170525
C	-1.39878174	4.53581968	0.60053128
C	-2.11699575	5.88776019	0.83772579
C	-3.40775585	5.94828681	-0.01635867
H	-2.44457580	6.68497480	-1.79442433
H	-3.94389977	5.84437764	-2.09924811
H	-2.37553205	5.97226590	1.87257738
H	-1.46711062	6.69289179	0.56512267
C	-2.41797993	3.68949573	-3.00079545

H -2.36755556 2.65481685 -2.73284586
H -3.36300640 3.89355633 -3.45925399
C -1.28532178 4.01918758 -3.99073925
H -1.39294717 3.41764097 -4.86906626
H -1.33574614 5.05386642 -4.25868886
H -0.34029526 3.81512696 -3.53228071
C -0.33572022 3.04989614 -1.13793800
H -0.06646315 2.97260542 -2.17061743
H 0.54236537 2.96194939 -0.53285038
C -4.14186974 7.27695125 0.24311712
H -5.03495158 7.31179171 -0.34517786
H -4.39500793 7.34792943 1.28031679
H -3.50571583 8.09429557 -0.02550243
C -4.32121301 4.77090363 0.37218330
H -5.21601708 4.80532812 -0.21351341
H -3.81087658 3.84828790 0.18987335
C -4.68132425 4.87185180 1.86608068
H -5.19166077 5.79446752 2.04839063
H -5.31599900 4.05379986 2.13604167
H -3.78652014 4.83742735 2.45177741
C -1.32165303 1.92271985 -0.77875824
H -1.59091010 2.00001060 0.25392115
H -2.19973865 2.01066654 -1.38384587
C -0.65627016 0.55772518 -1.03499094
H 0.22181544 0.46977845 -0.42990329
H -0.38701303 0.48043449 -2.06767036
C -1.64220298 -0.56945116 -0.67581119
H -1.17989151 -1.51785653 -0.85384302
H -1.91146008 -0.49216049 0.35686823
H -2.52028859 -0.48150444 -1.28089884
H -1.74820909 5.11488669 -2.48941665
H -0.31440798 5.19618431 -1.13256472
H -2.06184857 3.73707097 0.85984367
H -0.51966638 4.48159197 1.20808015

==

Cycloparaffins-a_R1-butyl_R2-methyl_R3-butyl
0 1
C -0.54915999 -2.28195700 -0.79302447
C -1.73465656 -1.37390343 -0.41594254
C -1.83964499 -0.69518816 0.96256293
C -0.44558806 -0.41178056 1.55331702
C 0.47324753 -1.64214317 1.44247610
C 0.73720356 -1.86121528 -0.05903413
H -0.81108194 -3.28028306 -0.51078214
H -0.36455390 -2.24015654 -1.84614779
H 1.40035851 -1.43143670 1.93336185
H 0.02818116 -2.50517862 1.89190311
C -3.03706857 -1.57374061 -1.21310965
H -3.77522476 -0.87915063 -0.87022502

H	-2.84760267	-1.40841585	-2.25314971
C	-3.54980697	-3.01115495	-1.00687786
H	-4.45474194	-3.14993285	-1.56072106
H	-2.81169506	-3.70577571	-1.34977709
H	-3.73925956	-3.17646949	0.03316050
C	-2.59529648	0.64210801	0.85172027
H	-3.57384856	0.46706074	0.45586998
H	-2.67712296	1.08693571	1.82143219
C	1.79481014	-2.96116275	-0.26689978
H	1.98028049	-3.08442964	-1.31346645
H	2.70244924	-2.68089416	0.22559029
H	1.43652920	-3.88241943	0.14276677
C	1.25032066	-0.52877117	-0.63602373
H	1.44835106	-0.64531907	-1.68106323
H	0.50867213	0.22966260	-0.49595855
C	2.54580291	-0.12012690	0.08946845
H	3.28752669	-0.87847616	-0.05076326
H	2.34783831	-0.00376348	1.13453820
C	-1.82489465	1.59115658	-0.08495141
H	-0.84633261	1.76623948	0.31085657
H	-1.74305523	1.14642504	-1.05470711
C	-2.58076635	2.92834694	-0.19550070
H	-2.66258085	3.37301386	0.77428142
H	-3.55932346	2.75322856	-0.59129777
C	-1.81053715	3.87758762	-1.13210522
H	-2.33574905	4.80665758	-1.20882739
H	-0.83196766	4.05269435	-0.73633727
H	-1.72875075	3.43296543	-2.10190377
C	3.05864806	1.21252759	-0.48731656
H	2.31690534	1.97084278	-0.34699635
H	3.25656274	1.09622967	-1.53239861
C	4.35415980	1.62119792	0.23809278
H	5.09592976	0.86292828	0.09771846
H	4.15627708	1.73744194	1.28318871
C	4.86687935	2.95391113	-0.33862664
H	5.76701607	3.23785925	0.16538363
H	5.06475034	2.83768169	-1.38372789
H	4.12510453	3.71217663	-0.19823781
H	-1.96304555	-2.16949350	0.26212516
H	-2.37488330	-1.36147976	1.60636098
H	0.02030544	0.38192528	1.00752418
H	-0.56754352	-0.12448887	2.57678730

==

Cycloparaffins-a_R1-butyl_R2-methyl_R3-propyl

0 1

C	-3.05105430	5.82793866	-1.54803026
C	-2.22442517	4.55360092	-1.73639341
C	-0.99619897	4.44555373	-0.82913487
C	-1.45821386	4.55938593	0.63487977

C	-2.21848057	5.89370210	0.83811626
C	-3.47356175	5.92082541	-0.06911006
H	-2.45542787	6.67813533	-1.80745716
H	-3.91966592	5.80042331	-2.17225680
H	-2.52192876	5.97371589	1.86106422
H	-1.57772138	6.71408366	0.59052417
C	-2.30526289	3.68217348	-3.00352994
H	-2.24082106	2.64949162	-2.73092518
H	-3.23504106	3.86225676	-3.50150713
C	-1.14067916	4.03785591	-3.94630920
H	-1.19687181	3.43238904	-4.82673946
H	-1.20510168	5.07054505	-4.21890675
H	-0.21090787	3.85775883	-3.44833065
C	-0.28790494	3.09667360	-1.05359016
H	0.02623327	3.02416525	-2.07385690
H	0.56589539	3.03096559	-0.41201201
C	-4.25049437	7.23124985	0.15607197
H	-5.11887236	7.24295829	-0.46898077
H	-4.54831867	7.29757299	1.18164342
H	-3.62411917	8.06370504	-0.08798866
C	-4.37284620	4.72168352	0.28425792
H	-5.24332762	4.73302560	-0.33786664
H	-3.83271965	3.81178599	0.12533391
C	-4.79644857	4.81567444	1.76182697
H	-5.33671544	5.72546678	1.92084389
H	-3.92585822	4.80447818	2.38380199
C	-1.26074953	1.94671336	-0.73302973
H	-1.57479985	2.01918358	0.28727463
H	-2.11455668	2.01262019	-1.37456719
C	-0.55288095	0.59763405	-0.95766463
H	0.30091840	0.53162852	-0.31613254
H	-0.23882054	0.52518090	-1.97796550
C	-1.52592289	-0.55218241	-0.63719014
H	-1.03416601	-1.48956582	-0.79334163
H	-1.83994429	-0.47976202	0.38312541
H	-2.37974752	-0.48610861	-1.27869564
C	-5.69531669	3.61629948	2.11542928
H	-5.15506505	2.70652067	1.95624066
H	-6.56601211	3.62752461	1.49360929
C	-6.11850978	3.71014713	3.59314322
H	-6.65872857	4.61993229	3.75234871
H	-6.74304414	2.87681504	3.83891763
H	-5.24775838	3.69890452	4.21489946
H	-2.11186020	3.74511982	0.86859328
H	-0.60416447	4.52779117	1.27868844
H	-0.31877303	5.24289171	-1.05328899
H	-1.69237481	5.12438090	-2.46852943

==

Cycloparaffins-a_R1-butyl_R2-methyl_R3-ethyl

0 1

C	-3.09075789	5.63318519	-1.60798701
C	-2.12282059	4.44899256	-1.77981768
C	-0.98619319	4.30462813	-0.74895122
C	-1.51199749	4.44820965	0.67409697
C	-2.19028081	5.83260350	0.79468494
C	-3.43349840	5.83588439	-0.10581577
H	-2.62733962	6.49988512	-2.03104102
H	-4.01594378	5.43882712	-2.10914174
H	-2.49482607	5.99088000	1.80814474
H	-1.51058637	6.60657101	0.50504753
C	-2.03201831	3.78792527	-3.16774594
H	-1.36597970	2.95176249	-3.12165487
H	-3.00325831	3.45414864	-3.46805593
C	-1.50195417	4.81069605	-4.18978901
H	-1.43886416	4.35138311	-5.15412884
H	-2.16799282	5.64685879	-4.23588003
H	-0.53071423	5.14447273	-3.88947897
C	-0.29651841	2.94061271	-0.93712789
H	0.08820494	2.86985002	-1.93305983
H	0.50716583	2.84768313	-0.23687385
C	-4.19459399	7.16617775	0.04469386
H	-5.07803251	7.14187663	-0.55850250
H	-4.46647324	7.30753048	1.06987730
H	-3.56808938	7.97342089	-0.27271834
C	-4.31998992	4.66437822	0.35603503
H	-5.20984649	4.63492577	-0.23741912
H	-3.78441321	3.74515320	0.24165426
C	-4.69658331	4.85825828	1.83663894
H	-5.23216002	5.77748333	1.95101975
H	-3.80672672	4.88771064	2.43009308
C	-1.31726202	1.81219175	-0.69974943
H	-1.70198541	1.88295445	0.29618252
H	-2.12094627	1.90512132	-1.40000347
C	-0.62758715	0.44817628	-0.88792611
H	0.17609709	0.35524673	-0.18767204
H	-0.24286373	0.37741353	-1.88385806
C	-1.64833081	-0.68024465	-0.65054767
H	-1.16914117	-1.62796966	-0.78129384
H	-2.03305421	-0.60948198	0.34538433
H	-2.45201504	-0.58731502	-1.35080173
C	-5.58307482	3.68675220	2.29848976
H	-5.04749810	2.76752719	2.18410901
H	-5.84473390	3.82146108	3.32722104
H	-6.47293137	3.65729980	1.70503556
H	-2.21907122	3.67632721	0.89578190
H	-0.69209308	4.38284621	1.35848311
H	-0.28015105	5.09258212	-0.90874189
H	-1.33071896	5.07402138	-2.13592719

==

Cycloparaffins-a_R1-butyl_R2-butyl_R3-methyl

0 1

C	-3.03288158	5.23938679	-1.82904285
C	-1.93398863	4.16280120	-1.90244363
C	-0.83409977	4.04152684	-0.83126345
C	-1.33674460	4.52382245	0.54256185
C	-2.06608273	5.87522392	0.43215337
C	-3.35439185	5.61653553	-0.37136400
H	-2.66896384	6.10094507	-2.34881192
H	-3.93195892	4.89011133	-2.29225491
H	-2.32774307	6.21241439	1.41333189
H	-1.45232244	6.61415909	-0.03916574
C	-1.64358013	3.56783665	-3.29283232
H	-0.87697152	2.82596974	-3.21020911
H	-2.53370309	3.11916319	-3.68180355
C	-1.17536120	4.68621947	-4.24235323
H	-1.94199638	5.42805787	-4.32520869
H	-0.28533330	5.13503274	-3.85335104
C	-0.37615378	2.57812994	-0.68855532
H	-0.00880595	2.22597144	-1.62980381
H	0.40212034	2.51771216	0.04325439
C	-4.24607487	6.87209783	-0.38190766
H	-5.14434614	6.66705787	-0.92594685
H	-4.49205832	7.14336121	0.62347827
H	-3.72135850	7.67825404	-0.85062850
C	-4.09854929	4.45440345	0.31222858
H	-5.00404051	4.24879916	-0.21950209
H	-3.47783598	3.58285592	0.30990736
C	-4.43293707	4.84266487	1.76448429
H	-5.05362249	5.71424580	1.76676305
H	-4.95006443	4.03530253	2.23950040
H	-3.52745404	5.04823657	2.29623374
C	-1.56701909	1.70856211	-0.24446078
H	-1.93440997	2.06068792	0.69677679
H	-2.34526974	1.76898724	-0.97629005
C	-1.10903946	0.24517461	-0.10177701
H	-0.33086688	0.18472361	0.63014553
H	-0.74153253	-0.10692195	-1.04298380
C	-2.29998740	-0.62441443	0.34210752
H	-1.98181871	-1.64118114	0.44130404
H	-2.66751361	-0.27228934	1.28328935
H	-3.07811778	-0.56395452	-0.38986816
C	-0.88465852	4.09084093	-5.63251373
H	-0.11807763	3.34894894	-5.54953274
H	-1.77468406	3.64209758	-6.02153999
C	-0.41619498	5.20898225	-6.58217749
H	-1.18274006	5.95086400	-6.66522923
H	0.47382475	5.65774347	-6.19312211
C	-0.12540990	4.61340392	-7.97225641

H -1.01546882 4.16466189 -8.36129600
H 0.20014385 5.39021743 -8.63214497
H 0.64112108 3.87149073 -7.88914860
H -1.29720216 5.00623174 -2.06988758
H -0.01213763 4.64918755 -1.14757355
H -2.03691435 3.81698304 0.93631989
H -0.49492578 4.60329855 1.19826119

==

Cycloparaffins-a_R1-butyl_R2-butyl_R3-butyl
0 1
C -3.31856824 6.23360677 -2.55129066
C -3.46627384 4.72620319 -2.83827793
C -2.27503673 3.81723615 -2.48589531
C -1.65006263 4.25122445 -1.13100584
C -1.40431972 5.76342431 -1.03842283
C -2.77017529 6.47917906 -1.15074676
H -2.61363626 6.64335616 -3.24417052
H -4.26704074 6.71332966 -2.67432164
H -0.96321107 5.98735229 -0.08963513
H -0.74821810 6.08888983 -1.81849722
C -4.49617030 4.20292170 -3.85663421
H -4.02012772 4.06031870 -4.80423504
H -4.89481928 3.27097135 -3.51392677
C -5.63746051 5.22622942 -4.00438282
H -6.11344679 5.36895287 -3.05678070
H -5.23874460 6.15812249 -4.34719750
C -2.73835856 2.35910526 -2.31042191
H -3.45535662 2.30557398 -1.51799749
H -3.18532738 2.01503602 -3.21968237
C -2.58808472 7.99354541 -0.93804519
H -3.53506595 8.48308414 -1.03015761
H -2.18832178 8.17103967 0.03847586
C -3.73741261 5.93025200 -0.08553313
H -4.68266666 6.42379193 -0.17395413
H -3.86915987 4.87848290 -0.23152210
C -6.66752171 4.70295350 -5.02259119
H -6.19151895 4.56010223 -5.97017183
H -7.06634103 3.77113251 -4.67971874
C -7.80866358 5.72643649 -5.17048800
H -7.40978980 6.65823453 -5.51333438
H -8.28469546 5.86928354 -4.22291706
C -1.52640999 1.47322103 -1.96689260
H -1.07950523 1.81721256 -1.05757309
H -0.80938581 1.52687640 -2.75928616
C -1.98963201 0.01503124 -1.79167245
H -2.70664018 -0.03865415 -0.99925884
H -2.43654719 -0.32891251 -2.70098606
C -8.83874444 5.20327217 -6.18875091
H -9.23765052 4.27148121 -5.84590558

H	-9.63156000	5.91444937	-6.29151149
H	-8.36272783	5.06038933	-7.13633003
C	-0.77767504	-0.87087516	-1.44824375
H	-0.33071071	-0.52692306	-0.53895147
H	-1.09954777	-1.88402615	-1.32649312
H	-0.06068957	-0.81720560	-2.24068165
C	-3.15463912	6.18474937	1.31701927
H	-3.02275627	7.23649802	1.46301440
H	-2.20944839	5.69109729	1.40547239
C	-4.12212788	5.63594909	2.38210181
H	-4.25407308	4.58420818	2.23604179
H	-5.06726612	6.12968127	2.29360395
C	-3.53949633	5.89027037	3.78473340
H	-3.40740851	6.94198752	3.93078171
H	-2.59440839	5.39642157	3.87329922
C	-4.50722162	5.34165652	4.84969376
H	-4.63932855	4.28994005	4.70363821
H	-4.10245053	5.51833959	5.82428847
H	-5.45228300	5.83554005	4.76108187
H	-1.91433044	8.37870636	-1.67467401
H	-2.33478505	4.00282785	-0.34719596
H	-0.73145679	3.71921147	-0.99679626
H	-1.56743402	3.87599520	-3.28634847
H	-2.97443020	5.13501575	-3.69611677

==

Cycloparaffins-a_R1-butyl_R2-butyl_R3-propyl

0 1

C	-3.07744175	5.21095263	-1.87292299
C	-1.96738081	4.14463286	-1.92511555
C	-0.89741689	4.02238127	-0.82413765
C	-1.44316907	4.48714118	0.53927752
C	-2.18107009	5.83319187	0.42079267
C	-3.44362596	5.57098689	-0.42148728
H	-2.70656713	6.08066885	-2.37387644
H	-3.95981674	4.85847213	-2.36493012
H	-2.47351645	6.15867143	1.39722665
H	-1.56080265	6.58195394	-0.02589511
C	-1.63233380	3.56580910	-3.31231146
H	-0.86179659	2.82980159	-3.21508515
H	-2.50704309	3.11331928	-3.73068186
C	-1.14746451	4.69754395	-4.23732361
H	-1.91801834	5.43353710	-4.33451716
H	-0.27274227	5.15006448	-3.81900736
C	-0.43089707	2.56161416	-0.68212327
H	-0.03394068	2.22161196	-1.61578762
H	0.32680987	2.50083445	0.07092387
C	-4.34612892	6.81863355	-0.44530631
H	-5.22667816	6.61101980	-1.01664976
H	-4.62303104	7.07770526	0.55525866

H -3.81574707 7.63404306 -0.89106851
C -4.19616343 4.39554946 0.22938403
H -5.08428969 4.18723803 -0.32984456
H -3.56762671 3.52964067 0.23620055
C -4.57513751 4.76613007 1.67526218
H -5.20361109 5.63208072 1.66848618
H -3.68704844 4.97435482 2.23458690
C -1.62615639 1.67765456 -0.28014023
H -2.02313655 2.01764262 0.65351264
H -2.38388721 1.73838087 -1.03317183
C -1.15947510 0.21694227 -0.13809884
H -0.40170623 0.15622758 0.61489346
H -0.76254885 -0.12307768 -1.07176838
C -2.35468833 -0.66699340 0.26398736
H -2.03047312 -1.68191262 0.36262493
H -2.75154905 -0.32700355 1.19769091
H -3.11248635 -0.60622736 -0.48897110
C -0.81253639 4.11872151 -5.62454409
H -0.04187326 3.38281895 -5.52738979
H -1.68721728 3.66607974 -6.04281406
C -0.32795300 5.25054886 -6.54959578
H -1.09866737 5.98640000 -6.64673328
H 0.54669816 5.70322771 -6.13131466
C 0.00701974 4.67180014 -7.93684782
H -0.86762810 4.21908599 -8.35511597
H 0.34366722 5.45822243 -8.57959016
H 0.77775606 3.93597825 -7.83971144
C -5.32782384 3.59064363 2.32584908
H -4.69935263 2.72468322 2.33259159
H -6.21589911 3.38247422 1.76649019
C -5.70688811 3.96107798 3.77174946
H -6.33533358 4.82705230 3.76501000
H -6.22990053 3.14434635 4.22374402
H -4.81881425 4.16921588 4.33111428
H -1.33360030 4.99509537 -2.06629656
H -0.07216119 4.64003216 -1.11115645
H -2.14798014 3.77058864 0.90628288
H -0.62106546 4.56751364 1.21943184

==
Cycloparaffins-a_R1-butyl_R2-butyl_R3-ethyl
0 1
C -3.18040482 5.26976081 -1.84086174
C -2.50461596 3.88614223 -1.86388156
C -1.16586784 3.95606200 -1.10594004
C -1.42323020 4.37742376 0.35276347
C -2.09905454 5.76101313 0.37580894
C -3.43779324 5.69110631 -0.38215358
H -2.54096036 5.98690087 -2.31172669
H -4.11056342 5.22120122 -2.36750659

H	-2.27788477	6.05376068	1.38932553
H	-1.45962769	6.47817178	-0.09506054
C	-2.24725231	3.46471811	-3.32257124
H	-1.77775336	2.50335439	-3.33853429
H	-3.17741553	3.41617248	-3.84920319
C	-1.32687138	4.49678068	-4.00032864
H	-1.79635653	5.45815007	-3.98438274
H	-0.39671257	4.54532020	-3.47368865
C	-0.49004294	2.57247337	-1.12896385
H	-0.31122785	2.27969479	-2.14247549
H	0.44012387	2.62107606	-0.60234617
C	-4.11359524	7.07471932	-0.35908547
H	-5.04374846	7.02616594	-0.88573460
H	-4.29243612	7.36745155	0.65443656
H	-3.47415288	7.79188208	-0.82992874
C	-4.35811683	4.65892882	0.29551252
H	-5.28827887	4.61039997	-0.23112273
H	-3.88857917	3.69758892	0.27945502
C	-4.61548966	5.08019615	1.75424154
H	-5.08500306	6.04154625	1.77031625
H	-3.68533060	5.12869382	2.28088532
C	-1.41031321	1.54031986	-0.45121363
H	-1.58915252	1.83312302	0.56228732
H	-2.34047187	1.49169096	-0.97784591
C	-0.73445253	0.15674112	-0.47417728
H	0.19570954	0.20538516	0.05245084
H	-0.55561488	-0.13607392	-1.48767333
C	-1.65471234	-0.87541002	0.20359764
H	-1.18512310	-1.83672692	0.18764590
H	-1.83353986	-0.58259392	1.21709631
H	-2.58487548	-0.92405116	-0.32302207
C	-1.06950342	4.07531492	-5.45900724
H	-0.60003376	3.11393782	-5.47494977
H	-1.99966132	4.02678951	-5.98565172
C	-0.14908868	5.10735298	-6.13676505
H	-0.61855434	6.06873091	-6.12082232
H	0.78107017	5.15587534	-5.61012123
C	0.10827745	4.68588707	-7.59544257
H	-0.82187661	4.63736719	-8.12209180
H	0.74778709	5.40294681	-8.06634876
H	0.57774499	3.72450956	-7.61138286
C	-5.53584899	4.04802624	2.43186173
H	-5.06634352	3.08667145	2.41577354
H	-5.71466519	4.34070968	3.44540175
H	-6.46601124	3.99954217	1.90522226
H	-3.14406092	3.16902214	-1.39298654
H	-0.52644621	4.67320613	-1.57683184
H	-2.06264653	3.66026843	0.82364480
H	-0.49306200	4.42601419	0.87938538

===

Cycloparaffins-a_R1-butyl_R2-propyl_R3-methyl

0 1

C	-3.04647007	5.30306485	-1.79931485
C	-1.93601730	4.24165049	-1.91090683
C	-0.83386014	4.09502418	-0.84526716
C	-1.34043794	4.52338776	0.54487311
C	-2.08442077	5.86980783	0.48222328
C	-3.37058618	5.62544271	-0.32909191
H	-2.69242736	6.18618706	-2.28885870
H	-3.94217843	4.96046719	-2.27392935
H	-2.34881827	6.16962149	1.47474555
H	-1.47913743	6.63140400	0.03672195
C	-1.64036474	3.69850487	-3.32129396
H	-0.86586700	2.96240118	-3.26479782
H	-2.52598813	3.25419377	-3.72522047
C	-1.18470381	4.85402065	-4.23165148
H	-1.95919436	5.59013720	-4.28841515
H	-0.29913889	5.29846472	-3.82767126
C	-0.35974005	2.63267365	-0.75431687
H	0.01044250	2.31789735	-1.70761642
H	0.41993991	2.55520071	-0.02560115
C	-4.27576196	6.87089191	-0.29486732
H	-5.17237723	6.67542553	-0.84511324
H	-4.52353617	7.10397956	0.71962655
H	-3.76027342	7.69864178	-0.73532475
C	-4.10138381	4.43198992	0.31373595
H	-5.00529853	4.23557616	-0.22412311
H	-3.47135634	3.56780338	0.28007224
C	-4.43805322	4.76504059	1.77911690
H	-5.06806133	5.62924219	1.81280170
H	-4.94585521	3.93584627	2.22574760
H	-3.53414122	4.96143285	2.31695922
C	-1.54037946	1.73479988	-0.34024378
H	-1.91054479	2.04937941	0.61312667
H	-2.32014133	1.81231833	-1.06883374
C	-1.06584816	0.27251276	-0.24977666
H	-0.28604245	0.19503377	0.47878882
H	-0.69572945	-0.04194238	-1.20320593
C	-2.24626143	-0.62568236	0.16426980
H	-1.91648441	-1.64166700	0.22699041
H	-2.61634306	-0.31130913	1.11774029
H	-3.02611066	-0.54816168	-0.56424291
C	-0.88880867	4.31025085	-5.64175852
H	-0.11435414	3.57410620	-5.58484549
H	-1.77437530	3.86583624	-6.04570872
C	-0.43296492	5.46547510	-6.55239273
H	-0.22733796	5.08753849	-7.53208280
H	-1.20739200	6.20162701	-6.60938134
H	0.45258022	5.90990999	-6.14845318

H -1.30858948 5.09727879 -2.04918253
H -0.01889903 4.72237099 -1.14056126
H -2.03257833 3.79563895 0.91400000
H -0.49896309 4.58897219 1.20252552

==

Cycloparaffins-a_R1-butyl_R2-propyl_R3-butyl
0 1
C -3.06021138 5.92968386 -1.49200311
C -2.22093154 4.67125770 -1.72620606
C -0.98842763 4.54551940 -0.82703168
C -1.44641769 4.60469223 0.64147802
C -2.21987902 5.92320558 0.89237563
C -3.47843375 5.96796914 -0.00940973
H -2.47443581 6.79443742 -1.72430601
H -3.93071135 5.91416834 -2.11403348
H -2.52050359 5.96520432 1.91841426
H -1.58858121 6.75822983 0.67078582
C -2.29702932 3.84255489 -3.02201636
H -2.22071251 2.80195782 -2.78488016
H -3.23040834 4.02949447 -3.51064159
C -1.13960445 4.24248308 -3.95589387
H -1.21603195 5.28303950 -4.19315896
H -0.20620374 4.05570900 -3.46724250
C -0.26667718 3.21263545 -1.09925322
H 0.04435886 3.17791180 -2.12245597
H 0.59017599 3.13463845 -0.46315453
C -4.26796027 7.26192737 0.26259178
H -5.13864737 7.28579251 -0.35888412
H -4.56276896 7.29040764 1.29078112
H -3.65103449 8.10862784 0.04486074
C -4.36423709 4.74825165 0.30582802
H -5.23688721 4.77177531 -0.31291037
H -3.81542210 3.84987695 0.11444725
C -4.78388518 4.78756781 1.78701177
H -5.33259887 5.68595112 1.97858923
H -3.91119056 4.76386756 2.40568134
C -1.22580274 2.04196897 -0.81423815
H -1.53686770 2.07672610 0.20895626
H -2.08264749 2.11991441 -1.45035449
C -0.50403005 0.70910060 -1.08631772
H 0.35282017 0.63113868 -0.45018452
H -0.19296496 0.67433921 -2.10949457
C -1.46317086 -0.46152797 -0.80131232
H -0.96169598 -1.38763356 -0.99036870
H -1.77423423 -0.42675787 0.22187690
H -2.32001330 -0.38355520 -1.43743173
C -1.21560367 3.41355682 -5.25156264
H -1.13913630 2.37302045 -5.01422892
H -2.14900866 3.60028614 -5.74020894

C	-0.05825609	3.81343418	-6.18553230
H	-0.11106605	3.23737988	-7.08568880
H	-0.13475384	4.85394707	-6.42296044
H	0.87517435	3.62679143	-5.69688644
C	-5.66977424	3.56789123	2.10202793
H	-5.12112151	2.66950548	1.91024384
H	-6.54253573	3.59174225	1.48347002
C	-6.08917565	3.60687180	3.58330452
H	-6.63773914	4.50529803	3.77516100
H	-5.21640108	3.58290432	4.20183599
C	-6.97519527	2.38726555	3.89823257
H	-7.26655214	2.41432694	4.92744472
H	-7.84799085	2.41124927	3.27972282
H	-6.42665091	1.48882780	3.70634654
H	-1.69747712	5.27220226	-2.44019413
H	-0.32024443	5.35716646	-1.02619817
H	-2.09064674	3.77608015	0.84946152
H	-0.58979097	4.56032026	1.28112543

==

Cycloparaffins-a_R1-butyl_R2-propyl_R3-propyl

0 1

C	-3.07215883	5.27267429	-1.83868342
C	-1.95032870	4.21985607	-1.92045878
C	-0.88432007	4.07290400	-0.82008883
C	-1.44248489	4.48549204	0.55509392
C	-2.18809521	5.83173201	0.47535129
C	-3.44729009	5.59117419	-0.37959828
H	-2.70734308	6.15931328	-2.31307932
H	-3.95020409	4.92659545	-2.34331819
H	-2.48638552	6.12600057	1.45999679
H	-1.56891523	6.59602334	0.05451934
C	-1.60057480	3.69385706	-3.32565077
H	-0.81746187	2.96878129	-3.25118591
H	-2.46591609	3.23956584	-3.76328916
C	-1.13602994	4.86776809	-4.20813816
H	-1.92127947	5.59098860	-4.27916967
H	-0.27083990	5.32135060	-3.77190386
C	-0.39851845	2.61348432	-0.72933498
H	0.01104621	2.31613624	-1.67215830
H	0.35413254	2.53536001	0.02729613
C	-4.35096357	6.83874326	-0.37467179
H	-5.23038059	6.64396680	-0.95208174
H	-4.62964887	7.07320569	0.63178878
H	-3.82078488	7.66554368	-0.79948405
C	-4.20571616	4.40190401	0.23852631
H	-5.08830636	4.20514421	-0.33364457
H	-3.57879928	3.53404157	0.23446145
C	-4.59910610	4.74909654	1.68638433
H	-5.22052633	5.61987179	1.68647488

H -3.71569222 4.94046047 2.25906542
C -1.58177510 1.69401280 -0.37386628
H -1.99192177 1.98731694 0.57045975
H -2.33510516 1.77442160 -1.12941900
C -1.08903641 0.23601236 -0.29266207
H -0.33716865 0.15459912 0.46477433
H -0.67689613 -0.05153524 -1.23707776
C -2.26885307 -0.69233591 0.05500590
H -1.92347206 -1.70449028 0.10341476
H -2.67930604 -0.41006874 1.00182485
H -3.02269272 -0.60870207 -0.69997865
C -0.78905621 4.34887790 -5.61654491
H -0.00066276 3.62860672 -5.54778932
H -1.65342741 3.89169811 -6.04999132
C -0.33531013 5.52525982 -6.50076200
H -0.09706242 5.16516187 -7.47990440
H -1.12393706 6.24544773 -6.56775198
H 0.53002638 5.98311185 -6.06889552
C -5.37068463 3.57167383 2.31068083
H -4.75156792 2.69897859 2.31250007
H -6.25427661 3.38107846 1.73823133
C -5.76323324 3.92540627 3.75807841
H -6.38095505 4.79921438 3.75726988
H -6.30202258 3.10849087 4.19135039
H -4.87974434 4.11385323 4.33155400
H -1.32721335 5.08243490 -2.03086858
H -0.06609278 4.70940601 -1.08113337
H -2.14500735 3.75302362 0.89412263
H -0.62481304 4.54816667 1.24254118

==
Cycloparaffins-a_R1-butyl_R2-propyl_R3-ethyl
0 1
C -3.06645086 5.92121914 -1.48718632
C -2.22545028 4.66464165 -1.72495031
C -1.00006908 4.53157816 -0.81721067
C -1.46956163 4.57896671 0.64810002
C -2.24488203 5.89554336 0.90359536
C -3.49631760 5.94780027 -0.00764003
H -2.47877343 6.78769267 -1.70795147
H -3.93202959 5.91081173 -2.11611270
H -2.55356105 5.92930522 1.92754877
H -1.61179425 6.73224020 0.69369471
C -2.29142352 3.84644582 -3.02798690
H -2.21700149 2.80394025 -2.79869955
H -3.22093591 4.03743543 -3.52238309
C -1.12666032 4.25385811 -3.94945538
H -1.20114251 5.29636108 -4.17872179
H -0.19714774 4.06291198 -3.45502549
C -0.27628206 3.20087739 -1.09451060

H	0.04277472	3.17435968	-2.11549193
H	0.57554097	3.11761806	-0.45238985
C	-4.28776437	7.23968676	0.26840380
H	-5.15352269	7.26864933	-0.35970490
H	-4.59067426	7.26005265	1.29443512
H	-3.66903888	8.08796087	0.06228347
C	-4.38486877	4.72600604	0.29104611
H	-5.25243078	4.75446362	-0.33457820
H	-3.83470022	3.82895463	0.09731407
C	-4.81662266	4.75432170	1.76903224
H	-5.36672343	5.65139848	1.96279533
H	-3.94905447	4.72578563	2.39465683
C	-1.23781342	2.02818383	-0.82648156
H	-1.55686315	2.05479954	0.19450503
H	-2.08962232	2.11147837	-1.46864661
C	-0.51427083	0.69731280	-1.10361317
H	0.33756053	0.61396451	-0.46144837
H	-0.19521852	0.67065136	-2.12457183
C	-1.47595930	-0.47525428	-0.83549767
H	-0.97329586	-1.39999568	-1.02803936
H	-1.79500949	-0.44857281	0.18547754
H	-2.32776832	-0.39189095	-1.47765133
C	-1.19244229	3.43572715	-5.25251595
H	-1.11797317	2.39323011	-5.02325887
H	-2.12192317	3.62665482	-5.74703800
C	-0.02759395	3.84324018	-6.17380098
H	-0.07316976	3.27473198	-7.07914010
H	-0.10211434	4.88571745	-6.40315234
H	0.90185408	3.65240445	-5.67923817
C	-5.70530003	3.53258506	2.06767524
H	-5.15522828	2.63551962	1.87384396
H	-6.00525541	3.55219792	3.09457984
H	-6.57289741	3.56118271	1.44207883
H	-1.69639676	5.27125819	-2.42994316
H	-0.33032902	5.34474108	-1.00459221
H	-2.11543826	3.74877801	0.84432029
H	-0.61798575	4.52931430	1.29404815

==

Cycloparaffins-a_R1-butyl_R2-ethyl_R3-methyl

0 1

C	-2.94726218	5.94177387	-1.43301052
C	-2.23013606	4.62150560	-1.72523680
C	-0.95062575	4.39373650	-0.91639982
C	-1.29654033	4.48227904	0.58103146
C	-1.94232633	5.85713897	0.88526251
C	-3.25432873	6.00648762	0.07561440
H	-2.31226389	6.75805610	-1.70752918
H	-3.85834420	5.99868003	-1.99121410
H	-2.16474934	5.91814861	1.93010980

H	-1.26370036	6.64015160	0.61832043
C	-2.46451085	3.80748850	-3.01133346
H	-2.45495429	2.76304116	-2.77908693
H	-3.41241407	4.07061928	-3.43221549
C	-1.34836806	4.11798310	-4.02593604
H	-1.35792459	5.16243045	-4.25818249
H	-0.40046484	3.85485226	-3.60505405
C	-0.35878807	3.00894025	-1.23837423
H	-0.12525325	2.95456313	-2.28116117
H	0.53227082	2.85982519	-0.66506913
C	-3.91640891	7.35808557	0.40185482
H	-4.82444053	7.45430350	-0.15592711
H	-4.13401095	7.40526002	1.44843205
H	-3.25079198	8.15379049	0.13973304
C	-4.21018311	4.86008622	0.45470047
H	-5.11987428	4.95587996	-0.10044414
H	-3.74993258	3.92159184	0.22601341
C	-4.51917902	4.92654368	1.96191810
H	-4.97942957	5.86503804	2.19060516
H	-5.18331161	4.13001802	2.22530903
H	-3.60948787	4.83074997	2.51706275
C	-1.38552447	1.91702059	-0.88464578
H	-1.61905934	1.97139776	0.15814114
H	-2.27658336	2.06613556	-1.45795092
C	-0.79368663	0.53222438	-1.20662007
H	0.09737229	0.38310946	-0.63331496
H	-0.56015180	0.47784717	-2.24940701
C	-1.82042294	-0.55969533	-0.85289152
H	-1.40921092	-1.52185891	-1.07660099
H	-2.05395772	-0.50531819	0.18989543
H	-2.71148188	-0.41058039	-1.42619657
C	-1.58274294	3.30396612	-5.31203275
H	-0.80724115	3.51969939	-6.01698390
H	-1.57318641	2.25951877	-5.07978631
H	-2.53064616	3.56709698	-5.73291473
H	-1.71264398	5.18204941	-2.47549847
H	-0.23571133	5.15050383	-1.16358508
H	-1.98843184	3.70670282	0.83532976
H	-0.40278123	4.36669528	1.15786339

==

Cycloparaffins-a_R1-butyl_R2-ethyl_R3-butyl

0 1

C	-3.14190202	5.41042565	-1.81704432
C	-2.00764582	4.37538457	-1.93659008
C	-0.94312845	4.19709780	-0.83798953
C	-1.50904069	4.54947645	0.55064233
C	-2.27625344	5.88405594	0.52352717
C	-3.52647022	5.65607853	-0.34660313
H	-2.78721141	6.32214733	-2.25046120

1579	H	-4.01255466	5.07523048	-2.34095536
1580	H	-2.58300506	6.13125276	1.51837772
1581	H	-1.66982350	6.67672145	0.13777677
1582	C	-1.64957575	3.90621366	-3.35906980
1583	H	-0.86342586	3.18211758	-3.30849239
1584	H	-2.51083356	3.46625925	-3.81687823
1585	C	-1.18338962	5.11235977	-4.19542037
1586	H	-1.96948766	5.83648620	-4.24604079
1587	H	-0.32214779	5.55228491	-3.73760455
1588	C	-0.44465901	2.74049100	-0.79817350
1589	H	-0.03335598	2.47815202	-1.75050194
1590	H	0.30875033	2.64189548	-0.04484414
1591	C	-4.45659432	6.88205607	-0.28802270
1592	H	-5.32818309	6.69713225	-0.88054894
1593	H	-4.74634469	7.06178352	0.72620780
1594	H	-3.94153015	7.73906276	-0.66904398
1595	C	-4.25700389	4.42039109	0.21097697
1596	H	-5.13647129	4.23405224	-0.36929475
1597	H	-3.60941539	3.57012047	0.16023512
1598	C	-4.65377946	4.67659182	1.67677645
1599	H	-5.30129871	5.52691451	1.72766363
1600	H	-3.77424471	4.86283689	2.25699304
1601	C	-1.62241083	1.80332498	-0.47201361
1602	H	-2.03379249	2.06556957	0.48029122
1603	H	-2.37583932	1.90185778	-1.22536511
1604	C	-1.12362443	0.34684667	-0.43223176
1605	H	-0.37023816	0.24831049	0.32117123
1606	H	-0.71219251	0.08461069	-1.38451455
1607	C	-2.30128809	-0.59036473	-0.10620759
1608	H	-1.95470678	-1.60229188	-0.07855581
1609	H	-2.71273791	-0.32813826	0.84606096
1610	H	-3.05465362	-0.49184022	-0.85960359
1611	C	-0.82532138	4.64311471	-5.61784272
1612	H	-0.50137391	5.48115201	-6.19893116
1613	H	-0.03922959	3.91896154	-5.56722354
1614	H	-1.68659010	4.20318740	-6.07568039
1615	C	-5.38437547	3.44086551	2.23427315
1616	H	-4.73689574	2.59052304	2.18320361
1617	H	-6.26401037	3.25473101	1.65417682
1618	C	-5.78080904	3.69683069	3.70016960
1619	H	-6.42832423	4.54714177	3.75131633
1620	H	-4.90116745	3.88297794	4.28023928
1621	C	-6.51131013	2.46102120	4.25760368
1622	H	-6.78672669	2.63877519	5.27614649
1623	H	-7.39094709	2.27489352	3.67754088
1624	H	-5.86378387	1.61072967	4.20641086
1625	H	-1.39223077	5.24760587	-2.01020499
1626	H	-0.12999912	4.85202730	-1.07218800
1627	H	-2.20012119	3.79281308	0.85852727
1628	H	-0.69388667	4.59830341	1.24202594

==

Cycloparaffins-a_R1-butyl_R2-ethyl_R3-propyl

0 1

C	-3.04795073	5.88184735	-1.52057239
C	-2.21693202	4.61415748	-1.73314475
C	-0.99001249	4.49178667	-0.82597013
C	-1.45501455	4.57461170	0.63921392
C	-2.21991622	5.90215776	0.86799354
C	-3.47348937	5.94373716	-0.04084597
H	-2.45451287	6.73894184	-1.76168218
H	-3.91534708	5.86419782	-2.14683561
H	-2.52542359	5.96052240	1.89180276
H	-1.58130200	6.72937465	0.63821777
C	-2.29264919	3.76824791	-3.01785955
H	-2.22543064	2.73048132	-2.76609558
H	-3.22205993	3.95542150	-3.51389108
C	-1.12742038	4.14661995	-3.95103114
H	-1.19466103	5.18440191	-4.20281472
H	-0.19800794	3.95943707	-3.45499347
C	-0.27683177	3.15000541	-1.07611246
H	0.03915155	3.09889162	-2.09712359
H	0.57617424	3.07443250	-0.43456577
C	-4.25484304	7.24707130	0.20906244
H	-5.12208529	7.26869161	-0.41730923
H	-4.55479429	7.29191420	1.23518495
H	-3.63056106	8.08614301	-0.01704354
C	-4.37000525	4.73518619	0.28675175
H	-5.23905996	4.75622015	-0.33707790
H	-3.82685259	3.83016345	0.11125846
C	-4.79745071	4.79902123	1.76487587
H	-5.34062515	5.70406420	1.94026048
H	-3.92836519	4.77802851	2.38870281
C	-1.24621976	1.99057086	-0.78003309
H	-1.56226554	2.04184163	0.24093576
H	-2.09914599	2.06613456	-1.42166582
C	-0.53323579	0.64861674	-1.02983664
H	0.31968659	0.57302386	-0.38819760
H	-0.21716729	0.59726590	-2.05079272
C	-1.50278031	-0.51065409	-0.73367260
H	-1.00743013	-1.44307855	-0.90719837
H	-1.81885291	-0.45929174	0.28729273
H	-2.35571192	-0.43506048	-1.37531734
C	-1.20307426	3.30078909	-5.23573632
H	-0.39347650	3.56372223	-5.88406191
H	-1.13581362	2.26300619	-4.98398484
H	-2.13248308	3.48796004	-5.73178190
C	-5.69396656	3.59057558	2.09275943
H	-5.15077579	2.68551407	1.91749774
H	-6.56300708	3.61147184	1.46888303

C	-6.12150201	3.65468065	3.57084240
H	-6.66473451	4.55973418	3.74606111
H	-6.74437493	2.81503588	3.79870903
H	-5.25247209	3.63383950	4.19473243
H	-1.68540418	5.20129338	-2.45261764
H	-0.31480818	5.29566187	-1.03280876
H	-2.10646763	3.75373457	0.85523882
H	-0.60201341	4.53272780	1.28379355

==

Cycloparaffins-a_R1-butyl_R2-ethyl_R3-ethyl

0 1

C	-3.05085613	5.87966871	-1.51836386
C	-2.21859032	4.61287863	-1.73142871
C	-0.99474059	4.48852219	-0.82041292
C	-1.46475984	4.56717119	0.64334923
C	-2.23099821	5.89378026	0.87309293
C	-3.48144221	5.93739879	-0.03992958
H	-2.45696722	6.73763030	-1.75517523
H	-3.91611985	5.86336257	-2.14765072
H	-2.54007790	5.94922538	1.89598403
H	-1.59192028	6.72186356	0.64776037
C	-2.28942867	3.77033196	-3.01854437
H	-2.22265054	2.73192011	-2.76920790
H	-3.21716147	3.95841463	-3.51738776
C	-1.12104002	4.15163306	-3.94652765
H	-1.18789782	5.19001580	-4.19593838
H	-0.19334114	3.96366207	-3.44757583
C	-0.28016256	3.14768020	-1.07169819
H	0.03928138	3.09937119	-2.09174703
H	0.57070117	3.07076625	-0.42748434
C	-4.26403713	7.23977950	0.21094476
H	-5.12913057	7.26290227	-0.41831275
H	-4.56750438	7.28167714	1.23616552
H	-3.63922298	8.07964568	-0.01067531
C	-4.37860769	4.72766216	0.28120162
H	-5.24549831	4.75017095	-0.34560227
H	-3.83448217	3.82334500	0.10504649
C	-4.81128237	4.78707739	1.75797889
H	-5.35534161	5.69144187	1.93413270
H	-3.94441317	4.76450478	2.38482727
C	-1.25001835	1.98704773	-0.78192476
H	-1.56948826	2.03546677	0.23811791
H	-2.10085864	2.06391244	-1.42619428
C	-0.53553871	0.64612525	-1.03291764
H	0.31530598	0.56927386	-0.38868220
H	-0.21609189	0.59764166	-2.05296997
C	-1.50543367	-0.51441045	-0.74296905
H	-1.00903661	-1.44612421	-0.91731552
H	-1.82487349	-0.46588473	0.27709046

```
H    -2.35627845  -0.43755672  -1.38720488
C    -1.19156813   3.30898405  -5.23360384
H    -0.37972386   3.57392332  -5.87829852
H    -1.12472021   2.27061313  -4.98417659
H    -2.11923471   3.49692851  -5.73261078
C    -5.70858913   3.57738564   2.07900741
H    -5.16451435   2.67303545   1.90288964
H    -6.00928321   3.61865947   3.10505890
H    -6.57542932   3.59996249   1.45211942
H    -1.68482798   5.20216586  -2.44749369
H    -0.31917249   5.29322382  -1.02280945
H    -2.11656796   3.74540803   0.85490207
H    -0.61394447   4.52390999   1.29074610

===========================================================
Cycloparaffins-a_R1-propyl_R2-methyl_R3-methyl
0 1
C    -2.99241822   5.96405465  -1.39773797
C    -2.36063230   4.61252867  -1.73978814
C    -1.03532805   4.32575376  -1.02945623
C    -1.26247690   4.42893166   0.48960757
C    -1.81979578   5.83171353   0.83808208
C    -3.18111422   6.04164001   0.12961307
H    -2.34334273   6.75058420  -1.72173222
H    -3.93958250   6.06307350  -1.88556487
H    -1.95945841   5.90206784   1.89659264
H    -1.12821225   6.58300333   0.51843245
C    -2.72869275   3.81107365  -3.00224664
H    -2.74937936   2.76711166  -2.76854719
H    -3.69270283   4.11768471  -3.35092162
C    -1.67952618   4.07085266  -4.09922631
H    -1.93525646   3.51399749  -4.97638900
H    -1.65883962   5.11481464  -4.33292580
H    -0.71551609   3.76424166  -3.75055132
C    -0.53354398   2.91553161  -1.39151462
H    -0.38235365   2.85129204  -2.44882950
H     0.39059997   2.72530695  -0.88686371
C    -3.75406169   7.42191453   0.50132993
H    -4.69635698   7.56006367   0.01358573
H    -3.88942427   7.47822586   1.56123841
H    -3.07449071   8.18647597   0.18743648
C    -4.15697620   4.93996395   0.58304806
H    -5.10074431   5.07776338   0.09806037
H    -3.75878130   3.98153513   0.32275016
C    -4.34762071   5.01938067   2.10913706
H    -4.74581560   5.97780955   2.36943490
H    -5.02565469   4.25393045   2.42418601
H    -3.40385266   4.88158118   2.59412484
C    -1.57943727   1.87154769  -0.95815013
H    -1.73062766   1.93578719   0.09916475
```

H -2.50358119 2.06177235 -1.46280106
C -1.07765314 0.46132559 -1.32020858
H -0.15350921 0.27110087 -0.81555762
H -1.80434524 -0.26403988 -1.01910476
H -0.92646274 0.39708615 -2.37752346
H -1.96789088 3.68568786 0.79763311
H -0.33384216 4.27207678 0.99747965
H -0.30730347 5.04914798 -1.33207979
H -1.87631812 5.14933091 -2.52857399

==

Cycloparaffins-a_R1-propyl_R2-methyl_R3-butyl

0 1

C -3.09955177 5.56327198 -1.75266905
C -2.00300667 4.49176430 -1.89896714
C -0.96030690 4.23178802 -0.79568497
C -1.53105351 4.55150387 0.59888048
C -2.24801854 5.91390084 0.61465942
C -3.49422091 5.76664097 -0.27845330
H -2.70561152 6.47696542 -2.14619039
H -3.97503080 5.28132208 -2.29939624
H -2.55856058 6.13394594 1.61468749
H -1.60763067 6.69754933 0.26723356
C -1.64405659 4.06436463 -3.33430328
H -0.88600101 3.30987007 -3.30273997
H -2.51497893 3.67489316 -3.81876867
C -1.12271568 5.28338127 -4.11783563
H -0.87343578 4.98636169 -5.11511870
H -1.88073273 6.03791002 -4.14936187
H -0.25174724 5.67282753 -3.63345063
C -0.51652129 2.75712464 -0.80664989
H -0.10273886 2.51634078 -1.76358383
H 0.22281125 2.60151634 -0.04898680
C -4.37877453 7.02364248 -0.18322071
H -5.24870647 6.89460971 -0.79270481
H -4.67504230 7.17506842 0.83373768
H -3.82718232 7.87461382 -0.52451895
C -4.27778173 4.53884565 0.22167783
H -5.15532774 4.40812331 -0.37640006
H -3.66153996 3.66737613 0.14611747
C -4.68555518 4.75345127 1.69113803
H -5.30142100 5.62519913 1.76672838
H -3.80811626 4.88370192 2.28946440
C -1.73234465 1.85300503 -0.53091238
H -2.14606465 2.09369225 0.42606499
H -2.47169915 2.00872280 -1.28852869
C -1.28859939 0.37833385 -0.54214267
H -0.54920216 0.22257927 0.21541245
H -2.13337387 -0.24980929 -0.35051851
H -0.87493928 0.13762325 -1.49915294

C	-5.47006343	3.52591454	2.19049431
H	-4.85423870	2.65415854	2.11497461
H	-6.34739943	3.39581312	1.59196566
C	-5.87832732	3.74026478	3.65984368
H	-6.49409552	4.61207673	3.73541670
H	-5.00105709	3.87025864	4.25847906
C	-6.66310065	2.51273175	4.15878648
H	-6.94682311	2.66159880	5.17968348
H	-7.54037268	2.38279286	3.56010896
H	-6.04737522	1.64090587	4.08320944
H	-1.35481851	5.34244975	-1.93158243
H	-0.12056893	4.86434899	-0.99472383
H	-2.25357591	3.80996029	0.86898897
H	-0.72382639	4.54323278	1.30117845

==

Cycloparaffins-a_R1-propyl_R2-methyl_R3-propyl

0 1

C	-3.03316204	5.92502439	-1.46225779
C	-2.33604771	4.59855730	-1.77416882
C	-1.04623678	4.35122506	-0.98775013
C	-1.36670111	4.43432155	0.51564541
C	-1.99166475	5.81449458	0.83872232
C	-3.31487437	5.98379109	0.05154542
H	-2.39341133	6.73574747	-1.74216148
H	-3.95249496	5.99575460	-2.00514272
H	-2.19634407	5.87148621	1.88741467
H	-1.30855187	6.59140697	0.56547935
C	-2.60062232	3.79531107	-3.06118059
H	-2.59925031	2.74939318	-2.83545742
H	-3.55217204	4.07180853	-3.46491342
C	-1.49765358	4.09944822	-4.09198570
H	-1.68148016	3.54133812	-4.98620175
H	-1.49902995	5.14536458	-4.31771171
H	-0.54610238	3.82295602	-3.68825370
C	-0.47560361	2.96184738	-1.32776536
H	-0.25969932	2.91136461	-2.37454143
H	0.42290368	2.79904335	-0.77001225
C	-3.95602512	7.34075688	0.39675502
H	-4.87185620	7.45076623	-0.14551101
H	-4.15599889	7.38385274	1.44701584
H	-3.28569283	8.13047634	0.12861293
C	-4.27751386	4.84597896	0.43925085
H	-5.19495698	4.95556613	-0.10036418
H	-3.83183945	3.90375514	0.19739955
C	-4.56116297	4.90655250	1.95169683
H	-5.00683951	5.84877957	2.19354221
H	-3.64371753	4.79696681	2.49131564
C	-1.50888341	1.87944512	-0.96396026
H	-1.72479319	1.92991869	0.08281686

H -2.40738614 2.04225682 -1.52171530
C -0.93824414 0.49006909 -1.30398936
H -0.03973782 0.32725926 -0.74623956
H -1.65616560 -0.26199243 -1.05121975
H -0.72234456 0.43959645 -2.35076366
C -5.52380525 3.76875162 2.33941849
H -5.07813265 2.82652986 2.09757178
H -6.44125293 3.87833752 1.79980421
C -5.80744284 3.82932518 3.85186035
H -6.25312230 4.77154553 4.09370607
H -6.47628544 3.03877005 4.12125879
H -4.88999222 3.71974583 4.39146797
H -2.06320125 3.66507852 0.77651387
H -0.46505248 4.30499379 1.07707849
H -0.32682203 5.10136076 -1.24198603
H -1.82444789 5.15787630 -2.52936761

==

Cycloparaffins-a_R1-propyl_R2-methyl_R3-ethyl
0 1
C -0.62522943 1.80163987 -0.24297210
C 0.77804918 1.16996018 -0.21994194
C 1.13913520 0.28427868 0.98862480
C 0.02731942 -0.71257725 1.29242708
C -1.26844485 0.08623578 1.56454595
C -1.68404112 0.78165421 0.26063832
H -0.59910572 2.68144752 0.36543240
H -0.90385852 2.06322769 -1.24239074
H -2.04450276 -0.58789108 1.86150907
H -1.10708996 0.80366112 2.34182676
C 1.87036585 1.80857026 -1.09779242
H 2.76999549 1.23428967 -1.02184283
H 1.54379906 1.82679817 -2.11657694
C 2.13790050 3.24748048 -0.61866554
H 2.89684778 3.69119008 -1.22860063
H 1.23827087 3.82176109 -0.69461509
H 2.46446731 3.22925257 0.40011895
C 2.46320008 -0.44967826 0.70620773
H 3.23589436 0.26703272 0.52135939
H 2.72695841 -1.04809354 1.55310246
C -3.01955700 1.52299682 0.45661908
H -3.31729993 1.97192764 -0.46788634
H -3.76954779 0.82892734 0.77392236
H -2.89974667 2.28322419 1.19998967
C -1.84311900 -0.32260620 -0.80091861
H -2.14692531 0.11556640 -1.72860739
H -0.90882236 -0.82694118 -0.93371924
C -2.90963156 -1.33164226 -0.33616367
H -3.84392820 -0.82730727 -0.20336305
H -2.60582526 -1.76981486 0.59152513

C	2.29554812	-1.35389258	-0.52906117
H	1.52285383	-2.07060355	-0.34421284
H	2.03178979	-0.75547729	-1.37595590
C	3.61961302	-2.08784953	-0.81147823
H	3.88337138	-2.68626480	0.03541651
H	3.50312757	-2.71610236	-1.66974948
H	4.39230729	-1.37113853	-0.99632658
C	-3.06870941	-2.43590269	-1.39772065
H	-2.13441275	-2.94023765	-1.53052130
H	-3.80972786	-3.13698623	-1.07480652
H	-3.37251573	-1.99773007	-2.32540942
H	-0.12148808	-1.37651933	0.46663216
H	0.29405504	-1.28116478	2.15871820
H	1.24882419	0.91111949	1.84882182
H	1.13087057	1.84233874	0.53393082

==

Cycloparaffins-a_R1-propyl_R2-butyl_R3-methyl

0 1

C	-3.03746865	5.31967781	-1.79952133
C	-1.99359748	4.18833463	-1.84831325
C	-0.90906353	4.02813486	-0.76671184
C	-1.39682732	4.55564421	0.59586744
C	-2.05643377	5.94017647	0.45984319
C	-3.35020333	5.73430509	-0.35009611
H	-2.62692356	6.15400000	-2.32889996
H	-3.94955907	5.00901609	-2.26477114
H	-2.30799010	6.30477986	1.43384047
H	-1.40285772	6.64028137	-0.01725583
C	-1.72329483	3.55850168	-3.22739943
H	-0.99560545	2.78041954	-3.12759525
H	-2.63196605	3.14923268	-3.61688392
C	-1.19242651	4.63735279	-4.18952378
H	-1.92017399	5.41537034	-4.28962271
H	-0.28390244	5.04678767	-3.79995350
C	-0.52640575	2.54581772	-0.59926537
H	-0.17050412	2.16180127	-1.53244395
H	0.24256395	2.45720016	0.13945344
C	-4.17772315	7.03258652	-0.38608669
H	-5.08122149	6.86455448	-0.93413001
H	-4.41697956	7.33086352	0.61325640
H	-3.60993475	7.80436220	-0.86239673
C	-4.15659155	4.62126887	0.34449537
H	-5.06738703	4.45326204	-0.19133413
H	-3.58031572	3.71983904	0.35983385
C	-4.48156656	5.04745162	1.78825862
H	-5.05791744	5.94883973	1.77289092
H	-5.04179855	4.27408067	2.27089049
H	-3.57079601	5.21555843	2.32410891
C	-1.76266558	1.74369178	-0.15217106

H	-2.11848459	2.12750542	0.78108849
H	-2.53172061	1.83244635	-0.89080842
C	-1.37990979	0.26135912	0.01475140
H	-0.61088284	0.17254377	0.75339289
H	-2.23885107	-0.29600741	0.32534421
H	-1.02406226	-0.12239708	-0.91854054
C	-0.92165468	4.00708605	-5.56829431
H	-1.83016635	3.59755798	-5.95792149
H	-0.19390136	3.22914095	-5.46807890
C	-0.39067874	5.08585354	-6.53051539
H	-1.11849343	5.86375473	-6.63082402
H	0.51775418	5.49545012	-6.14083789
C	-0.11965763	4.45551976	-7.90923373
H	-1.02808602	4.04591541	-8.29893285
H	0.24928175	5.20507448	-8.57777819
H	0.60816422	3.67761898	-7.80891263
H	-2.13457145	3.89085174	0.99425676
H	-0.55691640	4.60258986	1.25710485
H	-0.05526877	4.58896976	-1.08518841
H	-1.31399560	4.99614845	-2.02302502

==

Cycloparaffins-a_R1-propyl_R2-butyl_R3-butyl

0 1

C	-3.04604343	6.00213230	-1.46864492
C	-2.21350938	4.74543790	-1.73402909
C	-0.98312233	4.58954070	-0.83663400
C	-1.44298988	4.61376845	0.63229762
C	-2.20935649	5.92984415	0.91587523
C	-3.46629605	6.00478667	0.01383620
H	-2.45504902	6.86923206	-1.67785236
H	-3.91568242	6.00751997	-2.09204608
H	-2.51126815	5.94727654	1.94224359
H	-1.57302340	6.76666934	0.71660077
C	-2.29232282	3.95105845	-3.05100056
H	-2.22212739	2.90422292	-2.84096858
H	-3.22391086	4.15582428	-3.53586751
C	-1.13131305	4.36890260	-3.97245917
H	-1.20157951	5.41578401	-4.18224680
H	-0.19972142	4.16403608	-3.48763888
C	-0.26870823	3.25982770	-1.14181314
H	0.04375368	3.24940580	-2.16511883
H	0.58667483	3.16054561	-0.50668535
C	-4.24905515	7.29576765	0.31767221
H	-5.11863234	7.34034703	-0.30424297
H	-4.54531811	7.29963674	1.34582107
H	-3.62708817	8.14428533	0.12252140
C	-4.35935731	4.78249470	0.29673414
H	-5.23077536	4.82640702	-0.32263254
H	-3.81516132	3.88619280	0.08368670

C -4.78141049 4.78710941 1.77777439
H -5.32566629 5.68339265 1.99070345
H -3.90995445 4.74333116 2.39708751
C -1.23522257 2.08798015 -0.88811916
H -1.54781082 2.09861366 0.13515064
H -2.09049995 2.18716241 -1.52338223
C -0.52093433 0.75809926 -1.19278317
H 0.33432413 0.65890086 -0.55746756
H -1.19253701 -0.05602556 -1.01650771
H -0.20830780 0.74738732 -2.21601134
C -1.20992518 3.57509432 -5.28975561
H -2.14153227 3.77993763 -5.77455042
H -1.13958864 2.52818307 -5.08010006
C -0.04892624 3.99333061 -6.21105813
H -0.11931399 5.04024091 -6.42063232
H 0.88265751 3.78846290 -5.72622141
C -0.12739000 3.19972657 -7.52851385
H -1.05896987 3.40461450 -8.01338583
H 0.67933772 3.49038275 -8.16854961
H -0.05702981 2.15281832 -7.31901369
C -5.67432995 3.56481937 2.06111448
H -5.13008593 2.66848366 1.84831163
H -6.54579523 3.60845352 1.44180353
C -6.09628634 3.56986401 3.54215976
H -6.64058047 4.46619398 3.75486982
H -5.22480539 3.52632477 4.16144790
C -6.98912276 2.34759395 3.82576187
H -7.28230903 2.35116709 4.85481249
H -7.86059255 2.39110049 3.20646462
H -6.44481287 1.45126971 3.61308939
H -1.68556332 5.36162120 -2.43151497
H -0.30995388 5.40212149 -1.01403517
H -2.09223543 3.78380294 0.81812882
H -0.58756746 4.54813750 1.27174303

==

Cycloparaffins-a_R1-propyl_R2-butyl_R3-propyl
0 1
C -3.08351753 5.25843823 -1.83866905
C -2.01881050 4.14630606 -1.88047676
C -0.96059155 3.98426407 -0.77333928
C -1.49346296 4.47738398 0.58519460
C -2.17363568 5.85267679 0.45724016
C -3.44174688 5.63998855 -0.39065444
H -2.67397507 6.10958804 -2.34133096
H -3.97737265 4.94101399 -2.33384469
H -2.45720590 6.19435985 1.43076168
H -1.52020126 6.57284071 0.01079212
C -1.70107103 3.54751578 -3.26330648
H -0.96263665 2.78026472 -3.15853754

H	-2.59179475	3.13020442	-3.68439814
C	-1.16416021	4.65336061	-4.19092253
H	-1.90254599	5.42062076	-4.29586390
H	-0.27342612	5.07080966	-3.76992955
C	-0.55640929	2.50587351	-0.62297879
H	-0.16909705	2.14566396	-1.55309919
H	0.19408445	2.41651728	0.13443103
C	-4.29088596	6.92430590	-0.42447892
H	-5.17656748	6.75127755	-0.99941172
H	-4.56155868	7.19927454	0.57354042
H	-3.72453391	7.71469431	-0.87102606
C	-4.24638723	4.50016257	0.26125479
H	-5.13961704	4.32685682	-0.30177243
H	-3.65490118	3.60862401	0.27511406
C	-4.61680722	4.89293135	1.70354298
H	-5.20819614	5.78453557	1.68970571
H	-3.72356385	5.06611365	2.26659312
C	-1.78982987	1.67449852	-0.22415519
H	-2.17715843	2.03462889	0.70599718
H	-2.54030388	1.76392721	-0.98157686
C	-1.38566629	0.19608304	-0.07399213
H	-0.63518194	0.10665560	0.68343355
H	-2.24265847	-0.38157464	0.20306882
H	-0.99834363	-0.16403408	-1.00414830
C	-0.84654585	4.05386139	-5.57349414
H	-1.73732204	3.63638630	-5.99435875
H	-0.10815012	3.28664386	-5.46842451
C	-0.30964523	5.15927389	-6.50157137
H	-1.04800958	5.92651990	-6.60673790
H	0.58113595	5.57680590	-6.08078551
C	0.00789438	4.55940195	-7.88398035
H	-0.88289293	4.14182885	-8.30473090
H	0.38092345	5.32738516	-8.52891857
H	0.74627743	3.79216296	-7.77877605
C	-5.42166203	3.75318605	2.35536565
H	-4.83032963	2.86154403	2.36910790
H	-6.31495246	3.58010477	1.79235083
C	-5.79195417	4.14582352	3.79772523
H	-6.38330541	5.03745331	3.78402348
H	-6.35115911	3.35388937	4.25055091
H	-4.89868017	4.31890829	4.36076024
H	-2.22958461	3.79257671	0.95134682
H	-0.67227949	4.52618933	1.26940749
H	-0.10871359	4.56558058	-1.05844564
H	-1.34921962	4.96882897	-2.02193515

===

Cycloparaffins-a_R1-propyl_R2-butyl_R3-ethyl

0 1

C	-3.05508176	5.98648475	-1.46751075

2129	C	-2.22147848	4.73185979	-1.73928330
2130	C	-1.00086536	4.56344611	-0.83097684
2131	C	-1.47636032	4.57014194	0.63317670
2132	C	-2.24412780	5.88371897	0.92476792
2133	C	-3.49119667	5.97166260	0.01030729
2134	H	-2.46068050	6.85515590	-1.65981712
2135	H	-3.91799244	6.00078972	-2.10004419
2136	H	-2.55705107	5.88892673	1.94797261
2137	H	-1.60460746	6.72201782	0.74266473
2138	C	-2.28757423	3.95347543	-3.06642831
2139	H	-2.22128132	2.90405971	-2.86820715
2140	H	-3.21369926	4.16569436	-3.55853045
2141	C	-1.11627814	4.38006062	-3.97069350
2142	H	-1.18232391	5.42951713	-4.16865769
2143	H	-0.19014158	4.16749321	-3.47877726
2144	C	-0.28454634	3.23684856	-1.14519702
2145	H	0.03864043	3.23891987	-2.16522933
2146	H	0.56400585	3.12874856	-0.50241589
2147	C	-4.27528324	7.26006646	0.32163238
2148	H	-5.13805058	7.31370671	-0.30899778
2149	H	-4.58257609	7.25172303	1.34653046
2150	H	-3.65001584	8.10995474	0.14364644
2151	C	-4.38907932	4.74739072	0.26846847
2152	H	-5.25374071	4.80044940	-0.35957936
2153	H	-3.84398849	3.85290849	0.05009693
2154	C	-4.82697483	4.73413524	1.74487325
2155	H	-5.37202514	5.62862649	1.96328230
2156	H	-3.96227735	4.68103002	2.37286840
2157	C	-1.25462788	2.06284677	-0.91644288
2158	H	-1.57789452	2.06084835	0.10355170
2159	H	-2.10313373	2.17083465	-1.55930680
2160	C	-0.53819961	0.73626615	-1.23041980
2161	H	0.31032237	0.62829717	-0.58754035
2162	H	-1.21222215	-0.07940451	-1.07142700
2163	H	-0.21491306	0.73818976	-2.25041128
2164	C	-1.18301330	3.60216351	-5.29808715
2165	H	-2.10913920	3.81496605	-5.78994438
2166	H	-1.11717522	2.55270136	-5.10017884
2167	C	-0.01170074	4.02843912	-6.20245691
2168	H	-0.07739828	5.07794384	-6.40024268
2169	H	0.91443594	3.81547343	-5.71070193
2170	C	-0.07883066	3.25068915	-7.52992040
2171	H	-1.00498039	3.46371990	-8.02161340
2172	H	0.73499190	3.54677074	-8.15836040
2173	H	-0.01317550	2.20118074	-7.33210497
2174	C	-5.72489422	3.50991242	2.00311546
2175	H	-5.17988616	2.61540621	1.78463532
2176	H	-6.02901758	3.50069257	3.02895431
2177	H	-6.58965517	3.56303783	1.37520742
2178	H	-1.68537304	5.35564099	-2.42369027

H	-0.32499813	5.37735080	-0.99115211
H	-2.12852742	3.73879564	0.80179193
H	-0.62793101	4.49550154	1.28087060

==

Cycloparaffins-a_R1-propyl_R2-propyl_R3-methyl

0 1

C	-2.88912645	6.04632108	-1.38976254
C	-2.21808798	4.71284380	-1.72787482
C	-0.94306938	4.41685268	-0.93417498
C	-1.27863098	4.46912812	0.56731189
C	-1.87648073	5.85437684	0.91873365
C	-3.18660725	6.07313973	0.12192966
H	-2.22843688	6.84929531	-1.64199589
H	-3.80045584	6.15135089	-1.94053292
H	-2.09171654	5.88945042	1.96627531
H	-1.17326651	6.62241866	0.67276193
C	-2.48579534	3.94849453	-3.03772326
H	-2.51015549	2.89744015	-2.83874808
H	-3.42634924	4.25638298	-3.44449432
C	-1.36474732	4.25374863	-4.04850101
H	-1.34038756	5.30480357	-4.24747567
H	-0.42419293	3.94585939	-3.64173008
C	-0.39955612	3.02404330	-1.30333002
H	-0.17298213	2.99516028	-2.34866727
H	0.48874136	2.82708697	-0.74027035
C	-3.80141745	7.43499291	0.49470923
H	-4.70837778	7.57917828	-0.05441172
H	-4.01229090	7.45602848	1.54351352
H	-3.11075720	8.21599452	0.25403525
C	-4.17853726	4.94777155	0.47009734
H	-5.08715803	5.09150459	-0.07639065
H	-3.75112013	4.00220736	0.20909361
C	-4.47789419	4.97644098	1.98044934
H	-4.90531124	5.92200536	2.24145258
H	-5.16709265	4.19452947	2.22235801
H	-3.56927348	4.83270790	2.52693789
C	-1.46062449	1.95627357	-0.97836597
H	-1.68719789	1.98515621	0.06697094
H	-2.34892230	2.15323015	-1.54142497
C	-0.91711176	0.56346426	-1.34752157
H	-0.02881374	0.36650854	-0.78446306
H	-1.65434750	-0.17842796	-1.12173380
H	-0.69053859	0.53458109	-2.39285884
C	-1.63245376	3.48939985	-5.35834984
H	-2.57300761	3.79728826	-5.76512112
H	-1.65681357	2.43834551	-5.15937507
C	-0.51140581	3.79465450	-6.36912709
H	-0.69740882	3.26358063	-7.27921617
H	-0.48704710	4.84570892	-6.56810258

H	0.42914858	3.48676669	-5.96235561
H	-1.99491405	3.70935986	0.80095919
H	-0.38649302	4.30546682	1.13494285
H	-0.20437306	5.15683696	-1.16144384
H	-1.68569515	5.27943186	-2.46301819

==

Cycloparaffins-a_R1-propyl_R2-propyl_R3-butyl

0 1

C	-3.05403822	5.96306285	-1.49201057
C	-2.22137339	4.70234731	-1.73718436
C	-0.98943750	4.56249229	-0.83938199
C	-1.44694267	4.61134290	0.62965450
C	-2.21343871	5.93168821	0.89205633
C	-3.47183780	5.99092079	-0.00903946
H	-2.46376300	6.82668253	-1.71704324
H	-3.92471121	5.95742703	-2.11396353
H	-2.51370560	5.96644160	1.91847291
H	-1.57777718	6.76522364	0.67753684
C	-2.30196575	3.88527604	-3.04006893
H	-2.23112425	2.84227032	-2.81198922
H	-3.23438905	4.08135180	-3.52694666
C	-1.14246775	4.28724372	-3.97046052
H	-1.21332075	5.33026428	-4.19844873
H	-0.21008659	4.09112199	-3.48354624
C	-0.27457907	3.22832460	-1.12321888
H	0.03616233	3.20082780	-2.14674308
H	0.58193292	3.14044762	-0.48794996
C	-4.25426395	7.28674967	0.27421907
H	-5.12487275	7.32075396	-0.34687470
H	-4.54878787	7.30794548	1.30267908
H	-3.63272567	8.13190332	0.06371815
C	-4.36428721	4.77345988	0.29606295
H	-5.23675445	4.80678633	-0.32249636
H	-3.82036285	3.87370658	0.09734201
C	-4.78386701	4.80313281	1.77753016
H	-5.32774771	5.70292145	1.97619485
H	-3.91136751	4.76979284	2.39601184
C	-1.23970604	2.06018023	-0.84817342
H	-1.55056370	2.08778172	0.17530549
H	-2.09615844	2.14796587	-1.48354108
C	-0.52475950	0.72597308	-1.13165991
H	0.33165001	0.63820211	-0.49624925
H	-1.19533079	-0.08563930	-0.94055673
H	-0.21384928	0.69834139	-2.15512611
C	-1.22288704	3.47037842	-5.27349563
H	-2.15525153	3.66652890	-5.76045085
H	-1.15206037	2.42733322	-5.04555127
C	-0.06330422	3.87244107	-6.20376396
H	-0.11917675	3.30492296	-7.10912985

H -0.13411871 4.91548711 -6.43169396
H 0.86906816 3.67627803 -5.71681379
C -5.67638504 3.58586101 2.08300728
H -5.13260701 2.68601084 1.88432458
H -6.54896278 3.61918525 1.46462501
C -6.09563836 3.61584612 3.56456033
H -6.63936237 4.51573703 3.76320124
H -5.22301119 3.58250278 4.18286683
C -6.98822398 2.39872941 3.87036155
H -7.27940940 2.41965525 4.89976724
H -7.86088873 2.43206379 3.25210221
H -6.44454549 1.49880920 3.67171714
H -1.69490104 5.30668479 -2.44607540
H -0.31710159 5.37237940 -1.03165034
H -2.09549288 3.78433087 0.83056065
H -0.59048952 4.55695173 1.26875231

==

Cycloparaffins-a_R1-propyl_R2-propyl_R3-propyl
0 1
C 0.68563192 1.11625975 -0.82097702
C 2.15526813 0.79990085 -1.15530362
C 0.59900672 1.47247499 0.67515054
C 2.62589484 -0.40150126 -0.31462821
H 2.24144118 0.56313031 -2.19521925
H 2.76319219 1.65159535 -0.93186676
C -0.21769620 -0.08336924 -1.16267180
C -0.86251702 1.53221699 1.15559072
C 4.09548758 -0.71782811 -0.64907978
H 2.53974593 -0.16476923 0.72528943
H 2.01796001 -1.25319222 -0.53808946
C -0.11890178 -0.66804821 -2.58396665
C -1.51178048 -0.38193142 -0.38274251
C -1.41115798 0.09514293 1.07766209
H -0.88261446 1.85937496 2.17415258
H -1.44315207 2.20592444 0.56070699
H 4.42249725 -1.55258830 -0.06501215
H 4.18160969 -0.95452933 -1.68901669
H 4.70342206 0.13386021 -0.42561576
C -1.25400661 -0.09706702 -3.45410895
H 0.82564077 -0.40559311 -3.01273591
H -0.20609310 -1.73350846 -2.53815177
H -1.71450547 -1.43254914 -0.38020960
H -2.30659741 0.13893048 -0.87452984
C -0.43824311 -0.81240282 1.85325060
C -2.81320777 0.01683538 1.70991128
C -1.15509589 -0.68203273 -4.87528587
H -1.16681661 0.96841250 -3.50003707
H -2.19856383 -0.35942882 -3.02533161
C -0.35377194 -0.34170461 3.31714647

H -0.79136080 -1.82200308 1.82303021
H 0.53207280 -0.76038298 1.40527703
H -2.76216705 0.33257743 2.73098700
H -3.48304114 0.65497840 1.17231404
H -3.16858192 -0.99144953 1.66571017
C -2.29023112 -0.11137402 -5.74560294
H -1.24219384 -1.74747874 -4.82925753
H -0.21054541 -0.41957271 -5.30405376
C 0.61885871 -1.24932955 4.09295146
H -0.00053151 0.66785574 3.34736672
H -1.32414149 -0.39361307 3.76503599
H -2.20318218 0.95407419 -5.79168818
H -3.23479979 -0.37384438 -5.31684626
H -2.22143215 -0.51788196 -6.73298629
C 0.70334266 -0.77861715 5.55683070
H 0.26557708 -2.25887461 4.06275765
H 1.58921656 -1.19746249 3.64506164
H 1.05659947 0.23093040 5.58704297
H 1.37914831 -1.40924219 6.09582341
H -0.26700603 -0.83050539 6.00474969
H 1.09176231 0.71815272 1.25226855
H 1.08251106 2.41539864 0.82354804
H 0.35081381 1.94552189 -1.40847156
H -0.76650963 0.77330296 -1.49408910

==

Cycloparaffins-a_R1-propyl_R2-propyl_R3-ethyl
0 1
C -3.05666878 5.95780828 -1.48928798
C -2.22519625 4.69726671 -1.73918982
C -1.00281881 4.54442205 -0.83048696
C -1.47505347 4.57929420 0.63426069
C -2.24058689 5.89900845 0.90271109
C -3.48952376 5.97118576 -0.01052835
H -2.46168282 6.82201471 -1.69915016
H -3.92093357 5.96128235 -2.12010019
H -2.55124786 5.92385487 1.92631605
H -1.60049159 6.73295567 0.70346248
C -2.29499819 3.89405646 -3.05126770
H -2.22947310 2.84851995 -2.83337683
H -3.22187943 4.09798718 -3.54541792
C -1.12510187 4.30219556 -3.96573339
H -1.19065376 5.34774264 -4.18355097
H -0.19822406 4.09824348 -3.47157139
C -0.28893266 3.21118537 -1.12105994
H 0.03210527 3.19347794 -2.14160202
H 0.56082539 3.11409445 -0.47811281
C -4.27143350 7.26606189 0.27836734
H -5.13554573 7.30892225 -0.35119184
H -4.57641825 7.27728616 1.30392336

H -3.64559413 8.11167836 0.08309781
C -4.38808844 4.75291783 0.27236920
H -5.25404366 4.79513126 -0.35472090
H -3.84434352 3.85395870 0.06957935
C -4.82286192 4.76764548 1.74962086
H -5.36670410 5.66653331 1.95242844
H -3.95691150 4.72551578 2.37671336
C -1.26019060 2.04318568 -0.86809622
H -1.58126562 2.06097488 0.15242979
H -2.10991975 2.14027667 -1.51109364
C -0.54640224 0.70986739 -1.15846127
H 0.30330561 0.61279075 -0.51544817
H -1.22128870 -0.10163086 -0.98268562
H -0.22529347 0.69205226 -2.17897704
C -1.19484570 3.49911571 -5.27793254
H -2.12171462 3.70310450 -5.77213285
H -1.12934694 2.45357265 -5.06013689
C -0.02486013 3.90727584 -6.19230946
H -0.07330818 3.34935760 -7.10404439
H -0.09035691 4.95283277 -6.41005558
H 0.90199376 3.70323743 -5.69812448
C -5.72122500 3.54922251 2.03241703
H -5.17734934 2.65034486 1.82959458
H -6.02333296 3.55939225 3.05882018
H -6.58716901 3.59134419 1.40530351
H -1.68983176 5.30738901 -2.43635651
H -0.32627995 5.35427148 -1.00745410
H -2.12786228 3.75211674 0.81993042
H -0.62527904 4.51580257 1.28137105

===

Cycloparaffins-a_R1-propyl_R2-ethyl_R3-methyl
0 1
C -2.95571895 5.98048618 -1.44081664
C -2.25682272 4.65298029 -1.74420824
C -0.97813169 4.40286456 -0.94069470
C -1.31861744 4.48557788 0.55831333
C -1.94542906 5.86665094 0.87387660
C -3.25764144 6.03878648 0.06910746
H -2.31083428 6.79025045 -1.71156672
H -3.86755006 6.05319329 -1.99595419
H -2.16407587 5.92334485 1.91976349
H -1.25733615 6.64252474 0.61034676
C -2.50550374 3.85101992 -3.03518359
H -2.50900770 2.80495596 -2.81015486
H -3.45105604 4.12946215 -3.45147768
C -1.38825330 4.15386090 -4.05087981
H -1.38474933 5.19992485 -4.27590854
H -0.44270100 3.87541866 -3.63458572
C -0.40544428 3.01268633 -1.27391180

H -0.17559368 2.96246424 -2.31772521
H 0.48519723 2.84793846 -0.70424887
C -3.90098912 7.39666157 0.40656710
H -4.80925264 7.50863317 -0.14788734
H -4.11499219 7.43944661 1.45407468
H -3.22572382 8.18536360 0.14798269
C -4.22739283 4.90242351 0.44308801
H -5.13731389 5.01397448 -0.10872720
H -3.78015553 3.95957856 0.20660928
C -4.53122552 4.96250237 1.95162247
H -4.97846281 5.90534734 2.18810118
H -5.20501383 4.17295148 2.21146610
H -3.62130447 4.85095141 2.50343772
C -1.44542775 1.93190414 -0.92470669
H -1.67527837 1.98212624 0.11910672
H -2.33606924 2.09665201 -1.49436963
C -0.87274035 0.54172591 -1.25792378
H 0.01790117 0.37697800 -0.68826087
H -1.59532627 -0.20920718 -1.01529426
H -0.64288976 0.49150385 -2.30173719
C -1.63693432 3.35190053 -5.34185515
H -2.58248662 3.63034278 -5.75814923
H -0.86066292 3.56231602 -6.04756617
H -1.64043829 2.30583658 -5.11682643
H -1.73413764 5.21186510 -2.49210400
H -0.25404236 5.15188349 -1.18475469
H -2.01991282 3.71740102 0.80928961
H -0.42482562 4.35429796 1.13172573

==

Cycloparaffins-a_R1-propyl_R2-ethyl_R3-butyl
0 1
C -3.04810226 5.91397829 -1.51921352
C -2.22084774 4.64561728 -1.74231437
C -0.98956351 4.51612020 -0.84206668
C -1.44691401 4.59248480 0.62584324
C -2.20789657 5.92037577 0.86519690
C -3.46592218 5.96909159 -0.03703155
H -2.45413853 6.77107690 -1.75903152
H -3.91869132 5.90127874 -2.14113491
H -2.50813203 5.97410973 1.89081131
H -1.56880076 6.74748085 0.63640660
C -2.30482743 3.80632586 -3.03072892
H -2.23865899 2.76720163 -2.78441063
H -3.23628550 3.99799726 -3.52120355
C -1.14338134 4.18671219 -3.96773660
H -1.20952185 5.22587560 -4.21412796
H -0.21194754 3.99508541 -3.47719760
C -0.28038879 3.17413568 -1.10250712
H 0.03016856 3.12739994 -2.12538845

H 0.57578928 3.09376564 -0.46580331
C -4.24335129 7.27266800 0.22377724
H -5.11380737 7.29931358 -0.39790606
H -4.53783945 7.31273057 1.25166729
H -3.61857739 8.11167257 -0.00119490
C -4.36293840 4.76046672 0.28867646
H -5.23536097 4.78670257 -0.33025863
H -3.82243758 3.85545001 0.10505486
C -4.78210594 4.81643764 1.76944504
H -5.32267407 5.72137184 1.95321073
H -3.90963733 4.79022170 2.38832125
C -1.25039957 2.01508117 -0.80702890
H -1.56109537 2.06195919 0.21580782
H -2.10650209 2.09528116 -1.44386175
C -0.54107413 0.67310421 -1.06700977
H 0.31497253 0.59291155 -0.43009540
H -1.21504521 -0.13220175 -0.86172669
H -0.23030424 0.62619633 -2.08980059
C -1.22728753 3.34726570 -5.25610417
H -2.15867366 3.53897280 -5.74667700
H -0.42025786 3.61143239 -5.90712022
H -1.16122851 2.30812148 -5.00967880
C -5.67892941 3.60760165 2.09499449
H -5.13837737 2.70267582 1.91111422
H -6.55142780 3.63389709 1.47615616
C -6.09800038 3.66330928 3.57581771
H -6.63846351 4.56827907 3.75973895
H -5.22549886 3.63691148 4.19464957
C -6.99496296 2.45453860 3.90128306
H -7.28615849 2.49324426 4.93017042
H -7.86745178 2.48095212 3.28243517
H -6.45450554 1.54955992 3.71736949
H -2.09896208 3.77185733 0.84103987
H -0.59072621 4.54560260 1.26586737
H -0.31377551 5.31964146 -1.04837055
H -1.69171628 5.23521864 -2.46153343

==
Cycloparaffins-a_R1-propyl_R2-ethyl_R3-propyl
0 1
C -3.04210033 5.91922553 -1.51835288
C -2.21981410 4.64769950 -1.74177355
C -0.99450613 4.50842638 -0.83480275
C -1.46016786 4.58098659 0.63067922
C -2.21607375 5.91162508 0.87102800
C -3.46844329 5.97037579 -0.03849782
H -2.44250187 6.77432058 -1.75115063
H -3.90907799 5.91342216 -2.14544333
H -2.52219190 5.96262478 1.89504445
H -1.57150359 6.73647496 0.64951471

C	-2.30035476	3.81405040	-3.03408658
H	-2.24065190	2.77359200	-2.79164349
H	-3.22799637	4.01224793	-3.52917937
C	-1.13167064	4.19262720	-3.96275886
H	-1.19135463	5.23309664	-4.20517606
H	-0.20405931	3.99439426	-3.46763729
C	-0.29044779	3.16405444	-1.09666735
H	0.02603668	3.12007160	-2.11785446
H	0.56145826	3.07668898	-0.45518072
C	-4.24077164	7.27677921	0.22297473
H	-5.10724616	7.31042045	-0.40389956
H	-4.54133458	7.31413581	1.24919029
H	-3.61035658	8.11347168	0.00527709
C	-4.37356107	4.76500362	0.27680032
H	-5.24208180	4.79828226	-0.34726554
H	-3.83657293	3.85800795	0.09265184
C	-4.80138224	4.81694683	1.75524300
H	-5.33847111	5.72383475	1.93955680
H	-3.93278829	4.78373015	2.37922465
C	-1.26806218	2.00871144	-0.81197095
H	-1.58457884	2.05277133	0.20920363
H	-2.11992567	2.09606168	-1.45350932
C	-0.56412084	0.66424422	-1.07361440
H	0.28772864	0.57686923	-0.43205037
H	-1.24342774	-0.13844519	-0.87580958
H	-0.24757385	0.62015568	-2.09476184
C	-1.21211506	3.35901917	-5.25511670
H	-2.13971022	3.55725959	-5.75025479
H	-0.40006824	3.62202922	-5.90033589
H	-1.15245428	2.31855662	-5.01269161
C	-5.70620348	3.61128765	2.07037466
H	-5.16913387	2.70443193	1.88578194
H	-6.57493751	3.64459304	1.44659491
C	-6.13358102	3.66281643	3.54897485
H	-6.67069853	4.56962068	3.73361863
H	-6.76214918	2.82505308	3.76796905
H	-5.26479560	3.62955160	4.17269209
H	-1.68358207	5.23759376	-2.45546515
H	-0.31356297	5.30944547	-1.03374670
H	-2.11743169	3.76264341	0.83861642
H	-0.60803366	4.52732678	1.27555242

==

Cycloparaffins-a_R1-propyl_R2-ethyl_R3-ethyl

0 1

C	-2.95445503	5.97370338	-1.42816952
C	-2.26468346	4.64655529	-1.75323478
C	-0.98072928	4.38052951	-0.96333954
C	-1.30853501	4.44682494	0.53930658
C	-1.92585876	5.82703718	0.87659242

C -3.24373154 6.01526836 0.08478073
H -2.30777915 6.78351463 -1.69446997
H -3.87041974 6.05754970 -1.97486723
H -2.13566433 5.87218402 1.92484971
H -1.23608631 6.60266279 0.61674849
C -2.52789967 3.86144195 -3.05166237
H -2.53477488 2.81277049 -2.83919840
H -3.47542424 4.14949641 -3.45678189
C -1.41749364 4.17104829 -4.07281000
H -1.41061841 5.21971968 -4.28527400
H -0.46996912 3.88299386 -3.66769046
C -0.41771671 2.99169032 -1.31794153
H -0.19665850 2.95293578 -2.36414002
H 0.47671945 2.81573915 -0.75765137
C -3.87752702 7.37210135 0.44381773
H -4.78972319 7.49518318 -0.10175446
H -4.08274494 7.40329834 1.49349022
H -3.20047733 8.16055710 0.18917826
C -4.21604273 4.87922792 0.45305124
H -5.12987683 5.00186553 -0.08987351
H -3.77545596 3.93712989 0.20158627
C -4.50723165 4.92259798 1.96464909
H -4.94781849 5.86469599 2.21611394
H -3.59339753 4.79996044 2.50757375
C -1.46018196 1.91186686 -0.97319904
H -1.68124010 1.95062114 0.07299950
H -2.35461817 2.08781821 -1.53348908
C -0.89716939 0.52302775 -1.32780164
H -0.00273317 0.34707634 -0.76751156
H -1.62147975 -0.22723920 -1.08827291
H -0.67611118 0.48427349 -2.37400017
C -1.68070967 3.38593486 -5.37123751
H -2.62823405 3.67398955 -5.77635726
H -0.90919361 3.60105070 -6.08073602
H -1.68758520 2.33726343 -5.15877344
C -5.47954280 3.78655772 2.33291999
H -5.03895587 2.84445973 2.08145524
H -5.68186244 3.81669157 3.38318607
H -6.39337693 3.90919515 1.78999525
H -1.74535143 5.21185803 -2.49863872
H -0.25493679 5.12889219 -1.20433447
H -2.01157358 3.67911131 0.78679928
H -0.41075121 4.30427836 1.10372202

==

Cycloparaffins-a_R1-ethyl_R2-methyl_R3-methyl
0 1
C -2.52470222 4.66744923 -1.77969969
C -2.97172537 6.05408170 -1.31045029
C -1.16649498 4.21402003 -1.23848099

C	-2.98298435	6.06738253	0.23036872
H	-3.95121331	6.27180254	-1.68206973
H	-2.28402108	6.78937186	-1.67281917
C	-3.10986044	3.97524193	-3.02470527
H	-2.07665287	5.19582103	-2.59515969
C	-1.21402198	4.25697578	0.29960000
C	-0.85626226	2.78290821	-1.71521424
H	-0.40494818	4.87766608	-1.59135547
C	-4.01223157	5.04404953	0.74518881
C	-3.36561344	7.47520432	0.72355507
C	-1.58141319	5.68688101	0.76888725
C	-2.16587561	4.18946805	-4.22245307
H	-3.21292954	2.92728746	-2.83478901
H	-4.06924788	4.39340175	-3.24745648
H	-1.95468262	3.57109313	0.65440532
H	-0.25665168	3.98252251	0.69077453
C	-1.95095799	1.82486439	-1.20984970
H	-0.82922999	2.76121635	-2.78465275
H	0.09285704	2.47590995	-1.32814238
C	-4.02536515	5.05942959	2.28505600
H	-3.74643258	4.06657563	0.40052132
H	-4.98403161	5.29982019	0.37764826
H	-3.37753666	7.48732355	1.79342000
H	-2.64893327	8.18512491	0.36678864
H	-4.33622308	7.73132639	0.35312501
H	-0.85486374	6.38274399	0.40446907
H	-2.06280651	5.23742252	-4.41236933
H	-1.20648817	3.77130822	-3.99970186
H	-2.57244658	3.70851882	-5.08748942
H	-1.97799026	1.84655624	-0.14041120
H	-1.73540668	0.83052045	-1.54108645
H	-2.90007729	2.13186264	-1.59692156
H	-4.74049146	4.34841250	2.64275568
H	-3.05356511	4.80365893	2.65259656
H	-4.29116414	6.03690348	2.62972350
H	-1.59628477	5.71345243	1.83845389

==

Cycloparaffins-a_R1-ethyl_R2-methyl_R3-butyl

0 1

C	-3.09862933	5.68799416	-1.60165587
C	-2.16868003	4.48270781	-1.82797322
C	-1.02829449	4.26458599	-0.81436908
C	-1.53742746	4.36898520	0.61812341
C	-2.17220549	5.76749054	0.79811099
C	-3.42232002	5.84288390	-0.08960053
H	-2.61263592	6.55601070	-1.99568206
H	-4.03351050	5.54102498	-2.10095209
H	-2.46320647	5.89569090	1.81976854
H	-1.47173791	6.53123677	0.53177041

C -2.10979045 3.87318031 -3.24098688
H -1.46914982 3.01619626 -3.23333239
H -3.09324739 3.58060689 -3.54448668
C -1.55750054 4.91815655 -4.22820518
H -1.51658337 4.49465209 -5.20997282
H -2.19814059 5.77514156 -4.23586064
H -0.57404284 5.21073230 -3.92470676
C -0.38208006 2.88869674 -1.06122279
H -0.00812636 2.84481747 -2.06278838
H 0.42429736 2.74470830 -0.37280121
C -4.14126151 7.18869375 0.11900803
H -5.03010108 7.21425335 -0.47614905
H -4.40003013 7.29855955 1.15141701
H -3.49317686 7.98834378 -0.17330182
C -4.34012827 4.68166501 0.33560375
H -5.23545829 4.70189737 -0.24995123
H -3.83377379 3.75182598 0.18099051
C -4.69810150 4.82960061 1.82609764
H -5.20445406 5.75944013 1.98071116
H -3.80277029 4.80936659 2.41165014
C -1.43465825 1.78319768 -0.85738271
H -0.98566396 0.82722326 -1.02889815
H -1.80861096 1.82707658 0.14418175
H -2.24103520 1.92718587 -1.54580224
C -5.61591277 3.66838548 2.25130575
H -5.10956217 2.73854538 2.09669069
H -6.51124521 3.68862205 1.66575535
C -5.97388175 3.81632163 3.74180041
H -6.48023134 4.74616277 3.89641425
H -5.07854961 3.79608421 4.32735298
C -6.89169605 2.65510868 4.16700835
H -7.14041433 2.75789533 5.20261173
H -7.78702754 2.67534801 3.58145657
H -6.38534850 1.72526649 4.01239241
H -1.36095764 5.09687102 -2.16751488
H -0.29996926 5.03650709 -0.95068155
H -2.26577205 3.61076368 0.81690632
H -0.71413352 4.25265241 1.29158769

==

Cycloparaffins-a_R1-ethyl_R2-methyl_R3-propyl

0 1

C -3.15187073 5.64352596 -1.62849343
C -2.19073077 4.45699108 -1.82106909
C -1.03155054 4.31081492 -0.81589141
C -1.52490900 4.45668688 0.61849721
C -2.19716620 5.84272064 0.75316976
C -3.46029950 5.84815718 -0.11916440
H -2.69611229 6.50883975 -2.06256288
H -4.08852606 5.45090183 -2.10856918

H	-2.47849498	6.00246834	1.77308877
H	-1.52239849	6.61490942	0.44769796
C	-2.13264855	3.79465686	-3.21014786
H	-1.46764201	2.95700803	-3.17835566
H	-3.11114836	3.46287356	-3.48828088
C	-1.62337042	4.81543057	-4.24468004
H	-1.58301460	4.35523731	-5.20981919
H	-2.28837696	5.65307940	-4.27647223
H	-0.64487062	5.14721387	-3.96654702
C	-0.34937761	2.94508178	-1.01840828
H	0.01270153	2.87267770	-2.02267734
H	0.46963009	2.85084925	-0.33631840
C	-4.21479812	7.18030291	0.04732762
H	-5.11162429	7.15756177	-0.53584101
H	-4.46324421	7.32306101	1.07824695
H	-3.59375540	7.98586773	-0.28474356
C	-4.33884310	4.67903563	0.36344927
H	-5.24187842	4.65116536	-0.20983116
H	-3.80806061	3.75850058	0.23781017
C	-4.68162091	4.87490865	1.85198450
H	-5.21240339	5.79544370	1.97762360
H	-3.77858559	4.90277892	2.42526492
C	-1.36708670	1.81918077	-0.75722802
H	-0.89310941	0.87026229	-0.89793779
H	-1.72916584	1.89158485	0.24704104
H	-2.18609441	1.91341331	-1.43931790
C	-5.56016451	3.70578710	2.33459816
H	-5.02938203	2.78525205	2.20895906
H	-6.46319984	3.67791684	1.76131774
C	-5.90294233	3.90166013	3.82313339
H	-6.43372481	4.82219517	3.94877249
H	-6.51335898	3.08934840	4.15845588
H	-4.99990700	3.92953040	4.39641382
H	-1.40541675	5.07993377	-2.19540089
H	-0.32749164	5.09702926	-0.99215280
H	-2.22857162	3.68659375	0.85664734
H	-0.68997934	4.38997118	1.28433614

==

Cycloparaffins-a_R1-ethyl_R2-methyl_R3-ethyl

0 1

C	-3.04001171	5.97477598	-1.42398240
C	-2.38924081	4.64068485	-1.79744242
C	-1.07907488	4.33888587	-1.06557819
C	-1.34343617	4.38718287	0.45019807
C	-1.92033448	5.77281459	0.83366759
C	-3.26592335	5.99748684	0.10011573
H	-2.38976128	6.77686805	-1.70455805
H	-3.97597211	6.08390590	-1.93090329
H	-2.08592080	5.80506349	1.89028540

H	-1.22740761	6.53984695	0.55723731
C	-2.72038127	3.88124240	-3.09559232
H	-2.73818643	2.82961844	-2.89894538
H	-3.67820739	4.19283948	-3.45666904
C	-1.64734213	4.18688537	-4.15708626
H	-1.87742025	3.65922081	-5.05904757
H	-1.62953691	5.23850933	-4.35373322
H	-0.68951605	3.87528826	-3.79600949
C	-0.55730717	2.94588962	-1.46422576
H	-0.38027517	2.91979367	-2.51915641
H	0.35597763	2.74485573	-0.94422936
C	-3.85881999	7.35969842	0.50564397
H	-4.79023283	7.50796617	0.00028591
H	-4.02002714	7.37789584	1.56327393
H	-3.17814766	8.13970494	0.23513520
C	-4.24340062	4.87354006	0.49111679
H	-5.17634910	5.02135117	-0.01153519
H	-3.83130113	3.92773281	0.20735170
C	-4.47124473	4.89811107	2.01397048
H	-4.88334424	5.84391830	2.29773570
H	-3.53829619	4.75029996	2.51662234
C	-1.60478046	1.87979049	-1.09296797
H	-1.24225355	0.91192946	-1.36995039
H	-1.78181246	1.90588641	-0.03803732
H	-2.51806527	2.08082439	-1.61296437
C	-5.44872200	3.77416428	2.40497155
H	-5.03662251	2.82835703	2.12120641
H	-5.60702928	3.79123634	3.46305819
H	-6.38167049	3.92197541	1.90231960
H	-1.89051806	5.20827509	-2.55508148
H	-0.34990048	5.07771001	-1.32508461
H	-2.04997558	3.62849743	0.71495922
H	-0.42601042	4.21941746	0.97468329

==

Cycloparaffins-a_R1-ethyl_R2-butyl_R3-methyl

0 1

C	-2.85575310	6.08307619	-1.35899251
C	-2.19062202	4.76093248	-1.74926226
C	-0.93198089	4.41536369	-0.94948368
C	-1.29270662	4.39917754	0.54698243
C	-1.88443773	5.77170440	0.95423965
C	-3.17879848	6.04061766	0.14708360
H	-2.18390612	6.89086282	-1.56148580
H	-3.75658516	6.22287847	-1.91922314
H	-2.11724503	5.75883762	1.99852773
H	-1.17048230	6.54389692	0.75703682
C	-2.44250595	4.06247154	-3.09844979
H	-2.47937551	3.00340337	-2.95040783
H	-3.37326198	4.39830154	-3.50565077

C -1.30173001 4.40493483 -4.07464175
H -1.26486118 5.46400271 -4.22268206
H -0.37097528 4.06910498 -3.66743909
C -0.39433722 3.03664613 -1.37571792
H -0.15017768 3.05548135 -2.41731731
H 0.48245803 2.80465865 -0.80799475
C -3.78805545 7.38891982 0.57426651
H -4.68420686 7.56771196 0.01762859
H -4.01664398 7.36193278 1.61921572
H -3.08663051 8.17392214 0.38276388
C -4.18626285 4.90938830 0.42445176
H -5.08412536 5.08761962 -0.12960615
H -3.76266305 3.97332952 0.12568661
C -4.51114411 4.86886324 1.92924752
H -4.93473988 5.80492193 2.22801416
H -5.21113832 4.08288123 2.12196416
H -3.61328301 4.69062969 2.48330422
C -1.47003153 1.96470819 -1.11992326
H -1.09647366 1.00676660 -1.41607143
H -1.71419100 1.94587423 -0.07832350
H -2.34682797 2.19669557 -1.68764737
C -1.55360668 3.70647845 -5.42383303
H -2.48436094 4.04230779 -5.83103574
H -1.59047510 2.64741069 -5.27579579
C -0.41282789 4.04894610 -6.40001956
H -0.37595928 5.10801477 -6.54805692
H 0.51792575 3.71311492 -5.99281496
C -0.66470075 3.35049255 -7.74921194
H -1.59545418 3.68632355 -8.15641693
H 0.12791897 3.58844029 -8.42747112
H -0.70156948 2.29142449 -7.60117370
H -1.64083912 5.35685108 -2.44748870
H -0.18311436 5.15830382 -1.12875574
H -2.01944446 3.63596215 0.73205261
H -0.41186230 4.20018773 1.12092789

==
Cycloparaffins-a_R1-ethyl_R2-butyl_R3-butyl
0 1
C -3.09917363 5.24274009 -1.81298222
C -2.07155372 4.09617156 -1.85070831
C -1.04275968 3.88174342 -0.72489786
C -1.58719906 4.37184505 0.63012986
C -2.21831316 5.77101942 0.51017312
C -3.47473553 5.61411595 -0.36675914
H -2.65101815 6.08717709 -2.29357266
H -3.99253909 4.96318644 -2.33129137
H -2.51067013 6.10721519 1.48299060
H -1.53200253 6.47544164 0.08866712
C -1.74510997 3.50831782 -3.23616220

H	-1.03505382	2.71511747	-3.12873214
H	-2.64025672	3.12777042	-3.68202480
C	-1.15226434	4.60968845	-4.13455926
H	-1.86225688	5.40295295	-4.24200580
H	-0.25707534	4.99020986	-3.68875310
C	-0.69132088	2.38848144	-0.58971695
H	-0.29690878	2.02950831	-1.51732233
H	0.03981798	2.26251987	0.18132144
C	-4.27956164	6.92672312	-0.39773990
H	-5.15815211	6.79243482	-0.99352578
H	-4.56197742	7.19553919	0.59868806
H	-3.67774029	7.70419823	-0.81993629
C	-4.33049609	4.49228368	0.25041503
H	-5.21699020	4.35767188	-0.33348781
H	-3.76945342	3.58126239	0.26226235
C	-4.71793909	4.87532506	1.69086379
H	-5.27910160	5.78628417	1.67901917
H	-3.83152574	5.01003761	2.27485406
C	-1.95981020	1.59318327	-0.22922059
H	-1.71561742	0.55567580	-0.13529468
H	-2.35426515	1.95216471	0.69837879
H	-2.69089667	1.71913858	-1.00028364
C	-0.82595700	4.02163330	-5.51996294
H	-1.72116270	3.64117152	-5.96577779
H	-0.11601619	3.22831324	-5.41249647
C	-0.23300748	5.12291943	-6.41838261
H	-0.94294870	5.91622556	-6.52585882
H	0.66219240	5.50337484	-5.97254803
C	0.09331694	4.53487759	-7.80381304
H	-0.80187803	4.15440798	-8.24965263
H	0.50530441	5.30006177	-8.42802897
H	0.80326265	3.74157891	-7.69633545
C	-5.57354702	3.75324844	2.30779523
H	-5.01225838	2.84236050	2.31973608
H	-6.45986083	3.61843203	1.72370329
C	-5.96136750	4.13617857	3.74815508
H	-6.52278790	5.04697902	3.73617531
H	-5.07508767	4.27114634	4.33225919
C	-6.81675741	3.01393625	4.36505922
H	-7.08628154	3.28003089	5.36578595
H	-7.70301103	2.87888360	3.78092253
H	-6.25529339	2.10315505	4.37709622
H	-1.37207962	4.89775425	-1.96532858
H	-0.16619808	4.43830551	-0.98332109
H	-2.35323345	3.70672964	0.97024841
H	-0.77932809	4.38260983	1.33164138

==

Cycloparaffins-a_R1-ethyl_R2-butyl_R3-propyl

0 1

2929	C	-3.08995847	5.27656995	-1.79175767
2930	C	-2.07707622	4.11676786	-1.82387977
2931	C	-1.03899587	3.90543917	-0.70603050
2932	C	-1.56275582	4.42139444	0.64750273
2933	C	-2.17788376	5.82649150	0.51423944
2934	C	-3.44545383	5.67283492	-0.34711760
2935	H	-2.63652662	6.10850260	-2.28897435
2936	H	-3.99217589	5.00097070	-2.29666303
2937	H	-2.45567201	6.17998960	1.48520103
2938	H	-1.48752898	6.51633220	0.07556225
2939	C	-1.77275761	3.50518240	-3.20405408
2940	H	-1.07161113	2.70464343	-3.09261851
2941	H	-2.67729235	3.12971882	-3.63503513
2942	C	-1.17568484	4.58596469	-4.12436788
2943	H	-1.87677342	5.38655926	-4.23596052
2944	H	-0.27115189	4.96147638	-3.69340150
2945	C	-0.70458557	2.40997336	-0.55319648
2946	H	-0.32463137	2.03299440	-1.47971305
2947	H	0.03318090	2.28585204	0.21177400
2948	C	-4.23454041	6.99469202	-0.38805849
2949	H	-5.12105119	6.86290857	-0.97254202
2950	H	-4.50292792	7.28083636	0.60743386
2951	H	-3.62791309	7.75875592	-0.82749723
2952	C	-4.30816565	4.57043331	0.29474224
2953	H	-5.20247442	4.43876429	-0.27776609
2954	H	-3.75827752	3.65273578	0.31342899
2955	C	-4.67518473	4.97835464	1.73365559
2956	H	-5.22497858	5.89610525	1.71497704
2957	H	-3.78086257	5.10992740	2.30618124
2958	C	-1.97882422	1.63571786	-0.16791802
2959	H	-1.74640126	0.59667039	-0.06175687
2960	H	-2.35879824	2.01268507	0.75858916
2961	H	-2.71659774	1.75982456	-0.93288470
2962	C	-0.87138383	3.97389093	-5.50436301
2963	H	-1.77594860	3.59842246	-5.93527594
2964	H	-0.17035579	3.17327269	-5.39265745
2965	C	-0.27416107	5.05435161	-6.42494831
2966	H	-0.97515709	5.85498334	-6.53672966
2967	H	0.63041507	5.42986698	-5.99407507
2968	C	0.03006580	4.44197127	-7.80481548
2969	H	-0.87453073	4.06645236	-8.23564600
2970	H	0.44502941	5.19259880	-8.44454175
2971	H	0.73105580	3.64134047	-7.69299532
2972	C	-5.53805683	3.87601212	2.37544317
2973	H	-4.98828721	2.95824416	2.39407588
2974	H	-6.43238764	3.74449273	1.80291996
2975	C	-5.90502787	4.28387992	3.81439537
2976	H	-6.45478522	5.20165392	3.79577443
2977	H	-6.50456588	3.51796756	4.26030179
2978	H	-5.01068504	4.41539588	4.38690101

H	-1.36897869	4.90779800	-1.95732297
H	-0.15843948	4.44729608	-0.98161137
H	-2.33321944	3.77074567	1.00515263
H	-0.74733184	4.43200136	1.34023175

==

Cycloparaffins-a_R1-ethyl_R2-butyl_R3-ethyl

0 1

C	-3.05711235	6.00720528	-1.47749112
C	-2.22028320	4.75688084	-1.75873616
C	-0.99821164	4.58567218	-0.85299015
C	-1.47197689	4.58089871	0.61167435
C	-2.24291786	5.89034255	0.91314788
C	-3.49136110	5.98117397	0.00077476
H	-2.46538458	6.87883949	-1.66462818
H	-3.92081930	6.02335469	-2.10889195
H	-2.55460038	5.88771567	1.93674043
H	-1.60584976	6.73157081	0.73602018
C	-2.28525411	3.98721438	-3.09098577
H	-2.21528952	2.93675542	-2.89981493
H	-3.21252996	4.19957297	-3.58085921
C	-1.11617120	4.42385191	-3.99326856
H	-1.18645055	5.47425611	-4.18463687
H	-0.18896034	4.21182859	-3.50316098
C	-0.27867077	3.26329593	-1.17731933
H	0.04343362	3.27346769	-2.19763009
H	0.57085351	3.15290151	-0.53619131
C	-4.27847084	7.26534771	0.32173869
H	-5.14216965	7.32093557	-0.30740908
H	-4.58442827	7.24931924	1.34693543
H	-3.65569053	8.11804888	0.14867881
C	-4.38564295	4.75279931	0.25182504
H	-5.25120765	4.80767014	-0.37484347
H	-3.83840268	3.86128277	0.02688694
C	-4.82176193	4.72861031	1.72861022
H	-5.36903325	5.62013156	1.95352554
H	-3.95619627	4.67377521	2.35526056
C	-1.24541347	2.08515431	-0.95594000
H	-0.74549396	1.16637058	-1.18133196
H	-1.56751904	2.07495909	0.06437072
H	-2.09492472	2.19554621	-1.59706661
C	-1.18028323	3.65394234	-5.32543607
H	-2.10744273	3.86585211	-5.81569708
H	-1.10996665	2.60353374	-5.13406763
C	-0.01103183	4.09086217	-6.22739413
H	-0.08140373	5.14127892	-6.41877301
H	0.91607920	3.87898458	-5.73705959
C	-0.07478945	3.32095049	-7.55959371
H	-1.00189077	3.53280668	-8.04996911
H	0.73767654	3.62454164	-8.18621027

H	-0.00441416	2.27056209	-7.36821451
C	-5.71596899	3.50022375	1.97979787
H	-5.16870637	2.60869800	1.75488957
H	-6.01894553	3.48345242	3.00587325
H	-6.58154259	3.55503374	1.35317513
H	-1.68676703	5.38687271	-2.43946621
H	-0.32478154	5.40255914	-1.00823621
H	-2.12169096	3.74663870	0.77531283
H	-0.62257624	4.50407809	1.25784231

==

Cycloparaffins-a_R1-ethyl_R2-propyl_R3-methyl

0 1

C	-2.89182495	6.06851648	-1.37341953
C	-2.24310692	4.73597540	-1.75587661
C	-0.97006209	4.39531855	-0.97706186
C	-1.29935086	4.40568847	0.52668630
C	-1.87402717	5.78840620	0.92376894
C	-3.18340278	6.05252999	0.13957543
H	-2.21947468	6.86857973	-1.60307996
H	-3.80336256	6.20501931	-1.91689552
H	-2.08491115	5.79394842	1.97276715
H	-1.15968707	6.55269808	0.69909992
C	-2.52754201	4.01738889	-3.08791644
H	-2.56774615	2.96111736	-2.92186189
H	-3.46458601	4.35256281	-3.48099026
C	-1.40547043	4.33664379	-4.09321826
H	-1.36526630	5.39291531	-4.25927282
H	-0.46842644	4.00146988	-3.70014442
C	-0.44989749	3.00645127	-1.39188292
H	-0.22757170	3.00684782	-2.43853046
H	0.43720731	2.77805544	-0.83890877
C	-3.77532805	7.41146225	0.55734044
H	-4.68187428	7.58697557	0.01672136
H	-3.98206677	7.40286886	1.60714294
H	-3.07331586	8.18873551	0.33842230
C	-4.19168572	4.93244031	0.45637915
H	-5.09988955	5.10744495	-0.08161635
H	-3.78016563	3.98896410	0.16413973
C	-4.48511542	4.91837518	1.96810043
H	-4.89663553	5.86185137	2.26033984
H	-5.18567566	4.14013106	2.18821733
H	-3.57691156	4.74337059	2.50609592
C	-1.52648347	1.94573248	-1.09618336
H	-1.16507040	0.98074028	-1.38440318
H	-1.74880924	1.94533596	-0.04953582
H	-2.41358829	2.17412828	-1.64915748
C	-1.68990552	3.61805727	-5.42525809
H	-2.62694952	3.95323121	-5.81833183
H	-1.73010962	2.56178575	-5.25920360

C -0.56783396 3.93731211 -6.43055991
H -0.76546089 3.43803446 -7.35606811
H -0.52762976 4.99358362 -6.59661451
H 0.36920995 3.60213813 -6.03748602
H -2.02667246 3.65025658 0.73935023
H -0.40786535 4.21036194 1.08525692
H -0.22061913 5.13042822 -1.18406197
H -1.70450121 5.31695920 -2.47508571

==

Cycloparaffins-a_R1-ethyl_R2-propyl_R3-butyl
0 1
C -3.05772542 5.98856916 -1.49777575
C -2.22114753 4.73193214 -1.75024258
C -0.98834900 4.59120453 -0.85377974
C -1.44541628 4.63036029 0.61570210
C -2.21607410 5.94680616 0.88579752
C -3.47504948 6.00704927 -0.01450914
H -2.47026883 6.85526544 -1.71831526
H -3.92863304 5.98356706 -2.11939244
H -2.51604435 5.97487999 1.91250370
H -1.58314210 6.78353848 0.67568929
C -2.29984823 3.92181541 -3.05758647
H -2.22552851 2.87778623 -2.83531998
H -3.23319291 4.11764105 -3.54278362
C -1.14225270 4.33255488 -3.98656955
H -1.21642881 5.37662118 -4.20865460
H -0.20887248 4.13652601 -3.50148250
C -0.26907069 3.26110921 -1.14543472
H 0.04138156 3.24051989 -2.16918250
H 0.58796755 3.17253402 -0.51095773
C -4.26148512 7.29885873 0.27582651
H -5.13238832 7.33333610 -0.34485838
H -4.55576714 7.31369515 1.30445909
H -3.64271935 8.14711166 0.06960112
C -4.36359156 4.78524123 0.28444497
H -5.23621990 4.81902933 -0.33383880
H -3.81684818 3.88826765 0.08102378
C -4.78322270 4.80589891 1.76610924
H -5.32992724 5.70293384 1.96945236
H -3.91064919 4.77207866 2.38448683
C -1.23006070 2.08816504 -0.87678871
H -0.73025796 1.16400261 -1.07941272
H -1.54053370 2.10876579 0.14698292
H -2.08709651 2.17674302 -1.51127296
C -1.22132655 3.52278853 -5.29411168
H -2.15470333 3.71887830 -5.77914685
H -1.14715700 2.47871522 -5.07209124
C -0.06385326 3.93351074 -6.22317881
H -0.11894759 3.37094083 -7.13169621

H -0.13797222 4.97760582 -6.44515024
H 0.86955953 3.73734538 -5.73824606
C -5.67195754 3.58423883 2.06511693
H -5.12524017 2.68717094 1.86196201
H -6.54446096 3.61798023 1.44666051
C -6.09183294 3.60518202 3.54665279
H -6.63858522 4.50225324 3.74975116
H -5.21935828 3.57149023 4.16514348
C -6.98054732 2.38353865 3.84570133
H -7.27228617 2.39814421 4.87507182
H -7.85302743 2.41721486 3.22720216
H -6.43379386 1.48646941 3.64264110
H -1.69699214 5.34186039 -2.45603906
H -0.31882003 5.40439538 -1.04179682
H -2.09118076 3.80014470 0.81223749
H -0.58853328 4.57516723 1.25413070

==
Cycloparaffins-a_R1-ethyl_R2-propyl_R3-propyl
0 1
C -3.08295366 5.34710373 -1.77550413
C -2.06161283 4.19600071 -1.83796503
C -1.02687202 3.95844181 -0.72225637
C -1.55999761 4.42990034 0.64379728
C -2.18450899 5.83396601 0.54995138
C -3.44730778 5.69721084 -0.32117295
H -2.63348725 6.19678751 -2.24548987
H -3.98103949 5.08041197 -2.29242115
H -2.46888866 6.15623564 1.52983079
H -1.49730226 6.54146852 0.13516717
C -1.74694569 3.62842354 -3.23459488
H -1.04032713 2.83005199 -3.14419048
H -2.64683741 3.25945868 -3.68067356
C -1.15417194 4.74086151 -4.11928896
H -1.86083274 5.53919068 -4.20973647
H -0.25432192 5.10989392 -3.67322942
C -0.68274059 2.46140952 -0.61282069
H -0.29620639 2.11499000 -1.54847996
H 0.05257330 2.31939036 0.15139848
C -4.24580004 7.01402636 -0.32586151
H -5.12891998 6.89353467 -0.91787668
H -4.52035820 7.26824245 0.67658988
H -3.64291564 7.79524166 -0.73954594
C -4.30449535 4.56970364 0.28347155
H -5.19545838 4.44886903 -0.29658404
H -3.74786182 3.65590783 0.27682168
C -4.68045908 4.93109153 1.73248308
H -5.23724405 5.84477824 1.73925665
H -3.78951525 5.05205265 2.31257354
C -1.95330053 1.66737088 -0.25667288

H	-1.71424518	0.62719265	-0.18073627
H	-2.33980297	2.01375317	0.67902843
H	-2.68862954	1.80948363	-1.02087428
C	-0.83947751	4.17313091	-5.51582170
H	-1.73930491	3.80390710	-5.96179527
H	-0.13266159	3.37493200	-5.42535333
C	-0.24702472	5.28560912	-6.40071198
H	-0.02831527	4.89107775	-7.37097974
H	-0.95390872	6.08374441	-6.49126171
H	0.65275602	5.65494470	-5.95473691
C	-5.53732400	3.80317489	2.33684720
H	-4.98045055	2.88952996	2.33002643
H	-6.42825153	3.68220380	1.75676350
C	-5.91336646	4.16428015	3.78591865
H	-6.47027372	5.07789199	3.79278728
H	-6.50864963	3.38053273	4.20578456
H	-5.02243678	4.28528089	4.36601884
H	-1.35870330	4.99567869	-1.94436137
H	-0.14900611	4.51439471	-0.97750179
H	-2.32733286	3.76346287	0.97840258
H	-0.74760025	4.42539650	1.34014597

===

Cycloparaffins-a_R1-ethyl_R2-propyl_R3-ethyl

0 1

C	-2.82781295	6.07643026	-1.32911582
C	-2.25070964	4.72730163	-1.76452030
C	-0.97364647	4.30915008	-1.03137497
C	-1.26105641	4.29302320	0.48088568
C	-1.76155333	5.68856480	0.93010953
C	-3.07850574	6.03205103	0.19062441
H	-2.12645399	6.85176234	-1.55683470
H	-3.74670190	6.26818032	-1.84270605
H	-1.94332632	5.67478742	1.98446657
H	-1.01974614	6.42616943	0.70525049
C	-2.60353640	4.05879426	-3.10623998
H	-2.68695629	3.00121147	-2.96674107
H	-3.53481120	4.44596485	-3.46362841
C	-1.49596169	4.35517895	-4.13436170
H	-1.41254149	5.41276159	-4.27386013
H	-0.56468710	3.96800801	-3.77697398
C	-0.52834723	2.91037814	-1.49693418
H	-0.33481571	2.92951248	-2.54911289
H	0.36224381	2.62768750	-0.97555689
C	-3.59674111	7.40404105	0.66038511
H	-4.50880295	7.63444690	0.15052591
H	-3.77499214	7.37594081	1.71505888
H	-2.86650106	8.15500123	0.44196255
C	-4.12743501	4.94969444	0.50661766
H	-5.04110394	5.17959475	-0.00058520

H	-3.76713615	3.99718800	0.17826365
C	-4.37992496	4.90735003	2.02518789
H	-4.74022403	5.85985632	2.35354171
H	-3.46625618	4.67744999	2.53239124
C	-1.64337372	1.89093002	-1.19853597
H	-1.33397742	0.91905600	-1.52200910
H	-1.83690481	1.87179614	-0.14635747
H	-2.53396501	2.17361998	-1.71991254
C	-1.84878890	3.68667175	-5.47608116
H	-2.78006354	4.07384263	-5.83346937
H	-1.93220911	2.62908916	-5.33658222
C	-0.74121409	3.98305613	-6.50420288
H	-0.98636046	3.51857409	-7.43643666
H	-0.65779343	5.04063895	-6.64370130
H	0.19006033	3.59588484	-6.14681499
C	-5.42885348	3.82499258	2.34118037
H	-5.06855390	2.87248666	2.01282610
H	-5.60428476	3.79557082	3.39629081
H	-6.34252267	4.05489255	1.83397730
H	-1.70615292	5.30319769	-2.48333990
H	-0.19762431	5.01556632	-1.24033849
H	-2.01575313	3.56517281	0.69434946
H	-0.36444182	4.04300947	1.00859330

==

Cycloparaffins-a_R1-ethyl_R2-ethyl_R3-methyl

0 1

C	-2.93440397	6.02492154	-1.41360179
C	-2.29091449	4.68308332	-1.77160430
C	-1.00850024	4.36056407	-1.00047676
C	-1.31925053	4.40757572	0.50651402
C	-1.88850610	5.79988190	0.87701033
C	-3.20734169	6.04578394	0.10280737
H	-2.26464838	6.81874752	-1.67083268
H	-3.85251390	6.14881241	-1.94897571
H	-2.08645985	5.83097318	1.92808011
H	-1.17671513	6.55804064	0.62511951
C	-2.59197853	3.93262316	-3.08224154
H	-2.63050943	2.88071097	-2.89015796
H	-3.53367121	4.25878697	-3.47176950
C	-1.48225345	4.22669799	-4.10870649
H	-1.44372240	5.27861021	-4.30079002
H	-0.54056079	3.90053404	-3.71917858
C	-0.49397272	2.96171602	-1.38788560
H	-0.28454697	2.93661135	-2.43689019
H	0.39979062	2.74620515	-0.84048978
C	-3.79359877	7.41482390	0.49476687
H	-4.70666871	7.57777976	-0.03875790
H	-3.98740265	7.43180041	1.54693218
H	-3.09405941	8.18610759	0.24846184

C -4.21204504 4.93435564 0.45903530
H -5.12674033 5.09686750 -0.07183419
H -3.80448725 3.98380840 0.18470601
C -4.48684951 4.95710911 1.97414744
H -4.89440735 5.90765633 2.24847680
H -5.18492257 4.18488291 2.22165646
H -3.57215417 4.79459729 2.50501684
C -1.56721267 1.90917443 -1.05335285
H -1.20971629 0.93724757 -1.32252658
H -1.77663833 1.93427915 -0.00434825
H -2.46097606 2.12468518 -1.60074861
C -1.78331758 3.47623783 -5.41934373
H -2.72501031 3.80240160 -5.80887179
H -1.01227481 3.68056259 -6.13253684
H -1.82184850 2.42432566 -5.22726013
H -1.76099682 5.24612157 -2.51124959
H -0.26140193 5.08995429 -1.23442969
H -2.04416666 3.65799201 0.74634911
H -0.42102625 4.22525727 1.05864907

==

Cycloparaffins-a_R1-ethyl_R2-ethyl_R3-butyl
0 1
C -3.17963183 6.21311243 -2.63318568
C -3.42602666 4.70665738 -2.74519066
C -2.22492521 3.83117761 -2.37880512
C -1.78046715 4.18490801 -0.94799793
C -1.44831763 5.69577710 -0.86506112
C -2.71297362 6.52571870 -1.19830870
H -2.42058408 6.50065506 -3.33035723
H -4.07925453 6.75140793 -2.84721958
H -1.12435680 5.93110477 0.12720431
H -0.66977623 5.93148578 -1.56019424
C -4.57154238 4.10501516 -3.58035320
H -4.19471044 3.79741156 -4.53340273
H -4.97999903 3.25889303 -3.06835885
C -5.67158372 5.16403407 -3.78069249
H -6.04833048 5.47161805 -2.82761660
H -5.26308995 6.01011808 -4.29270910
C -2.61011096 2.34217801 -2.45679184
H -3.41194713 2.14507817 -1.77626362
H -2.92149021 2.10600454 -3.45286197
C -2.39506025 8.02817865 -1.08360269
H -3.27282255 8.59671465 -1.30990662
H -2.07362592 8.24973940 -0.08736666
C -3.82967219 6.15829781 -0.20355029
H -4.70767431 6.72768316 -0.42672220
H -4.05101889 5.11464549 -0.28535302
C -6.81727414 4.56268661 -4.61577244
H -6.44056082 4.25510553 -5.56885753

H	-7.22578990	3.71660652	-4.10375648
C	-1.39309774	1.47677100	-2.08062811
H	-1.08170564	1.71292730	-1.08452252
H	-0.59126839	1.67391753	-2.76115996
C	-1.27654405	8.39349044	-2.07710647
H	-0.39873328	7.82509964	-1.85067638
H	-1.59791814	8.17172896	-3.07332619
C	-0.95892070	9.89602680	-1.96275700
H	-0.63762221	10.11788325	-0.96654209
H	-1.83674221	10.46437286	-2.18928527
H	-3.50620513	6.37728426	0.79260715
H	-1.66071782	0.44218735	-2.13483816
H	-7.58152083	5.29855015	-4.75492227
C	0.15966363	10.26120658	-2.95622983
H	1.03748536	9.69292464	-2.72956310
H	-0.16161010	10.03918539	-3.95240805
C	0.47722981	11.76378062	-2.84220636
H	0.79848328	11.98585843	-1.84602273
H	1.25444608	12.01742352	-3.53248893
H	-0.40059671	12.33205217	-3.06890028
H	-3.09121315	4.82629939	-3.75438188
H	-1.42350480	4.02573447	-3.06055915
H	-2.57083372	3.96217884	-0.26199294
H	-0.91472047	3.60959980	-0.69424601

==

Cycloparaffins-a_R1-ethyl_R2-ethyl_R3-propyl

0 1

C	-3.12209240	6.29375676	-2.60313631
C	-3.61654738	4.84730803	-2.73363035
C	-2.51986630	3.77580180	-2.55537034
C	-1.87652222	3.98035011	-1.17446561
C	-1.30072662	5.42297668	-1.08300641
C	-2.45002516	6.45770956	-1.22852226
H	-2.39786402	6.48713583	-3.38171426
H	-3.93259448	6.96908736	-2.72104414
H	-0.82048706	5.55506878	-0.13927138
H	-0.59049232	5.57512835	-1.88361341
C	-4.94741757	4.46621142	-3.42363103
H	-4.75664248	4.21916257	-4.44863859
H	-5.36236273	3.60871321	-2.93231284
C	-5.95761435	5.61512364	-3.32237549
H	-6.12165502	5.84968939	-2.29319194
H	-5.57778625	6.47221546	-3.82274377
C	-3.10921778	2.35987114	-2.65986883
H	-3.86718775	2.23387487	-1.89641477
H	-3.55914708	2.22242025	-3.62483529
C	-1.88847565	7.89232497	-1.09431221
H	-2.69060959	8.59756694	-1.19816073
H	-1.43459469	8.00170013	-0.13299718

C -3.49300382 6.20964169 -0.10613242
H -4.28737439 6.92070221 -0.20687322
H -3.88343864 5.22592483 -0.20504202
C -7.28602463 5.18956405 -3.96681429
H -7.11835319 4.96905818 -4.99644812
H -7.66561121 4.32598642 -3.46763904
C -1.98761209 1.31705562 -2.46304780
H -1.54069381 1.46136889 -1.50759788
H -1.25486960 1.43346940 -3.23033178
C -0.82226298 8.14645121 -2.16479755
H -0.02040494 7.44729975 -2.04528157
H -1.25688132 8.03147650 -3.13234885
C -0.26969936 9.57505453 -2.00876171
H 0.16302046 9.69199772 -1.03854014
H -1.06936611 10.27885992 -2.13441929
H -3.02920341 6.32146753 0.83924737
H -2.40536390 0.33047683 -2.51362219
H -7.99989747 5.99309963 -3.88640891
C 0.80740271 9.83954780 -3.07781742
H 1.18395766 10.85050351 -2.97882757
H 1.61365340 9.14255957 -2.94708578
H 0.36627050 9.72046540 -4.04429880
H -3.41236794 4.97740300 -3.78089484
H -1.78070864 3.90731470 -3.31941778
H -2.63586427 3.85454780 -0.42510071
H -1.09416131 3.26902179 -1.03642827

==
Cycloparaffins-a_R1-ethyl_R2-ethyl_R3-ethyl
0 1
C 0.65713723 0.94495207 -0.56775987
C -0.81176068 0.51518197 -0.41138204
C -1.24339883 -0.91776349 -0.05508335
C -0.13535342 -1.65520646 0.71967151
C 1.24447969 -1.44863950 0.06444234
C 1.59481659 0.04051098 0.25381764
H 0.89923485 0.86452884 -1.60690414
H 0.79455096 1.95465145 -0.24137886
H 1.96827951 -2.04564129 0.57883778
H 1.23632999 -1.72454562 -0.96934166
C -1.89487415 1.47562919 -0.93674511
H -1.47431100 2.11076987 -1.68815911
H -2.69963347 0.90978823 -1.35756154
C -2.42503238 2.33633665 0.22502722
H -2.84559551 1.70119601 0.97644120
H -1.62027309 2.90217760 0.64584366
C -2.51429704 -0.90647209 0.81456615
H -2.31567133 -0.38882471 1.72971042
H -3.30246826 -0.41018609 0.28789134
C 3.04315725 0.32013554 -0.18858432

H	3.27260293	1.35258573	-0.02640765
H	3.71395340	-0.28811998	0.38146630
C	1.43623504	0.35660665	1.75266234
H	1.67475430	1.38492706	1.92749286
H	0.42620894	0.16889361	2.05184641
C	-3.50814581	3.29678386	-0.30033583
H	-3.08758268	3.93192450	-1.05174984
H	-4.31290513	2.73094291	-0.72115224
C	-2.93669050	-2.35448666	1.12511009
H	-2.14851930	-2.85077269	1.65178491
H	-3.13531621	-2.87213405	0.20996584
C	3.19582271	-0.01076306	-1.68484610
H	2.96637705	-1.04321326	-1.84702277
H	2.52502653	0.59749245	-2.25489672
C	4.64416334	0.26886152	-2.12724808
H	5.31495953	-0.33939403	-1.55719750
H	4.87360906	1.30131170	-1.96507139
H	2.09755858	-0.26437579	2.32005283
H	-3.81971717	-2.34664129	1.72934708
H	-3.87650247	3.89480789	0.50686961
H	4.75023603	0.03895147	-3.16685856
H	-0.07170350	-1.26529729	1.71406557
H	-0.38710066	-2.69461729	0.75356085
H	-1.43894139	-1.42711463	-0.97553136
H	-0.61512502	0.05424101	-1.35677502

References:

[1] Lide DR, editor. CRC Handbook of Chemistry and Physics. 86ª ed. Boca Raton: CRC Press; 2005.
